# Medicated and multifunctional composite alginate-collagen-hyaluronate based scaffolds prepared using two different crosslinking approaches show potential for healing of chronic wounds

**DOI:** 10.1007/s13346-024-01745-0

**Published:** 2024-12-11

**Authors:** Meena Afzali, Nessa Esfandiaribayat, Joshua Boateng

**Affiliations:** School of Science, Faculty of Engineering and Science, University of Greenwich at Medway, Chatham Maritime, Kent, ME4 4TB UK

**Keywords:** Calcium chloride, Fibroblast growth factor, Hyaluronic acid, Poly (ethylene glycol) diglycidyl ether, Fish collagen, Sodium alginate

## Abstract

Chronic wounds present significant challenges with high morbidity and mortality. A cost-effective dressing that can absorb large exudate volumes, is hemostatic and therapeutically active is of current interest. This study compares two crosslinking approaches on composite scaffolds comprising fish collagen (FCOL), hyaluronic acid (HA) and sodium alginate (SA) by respectively targeting HA and SA. Crosslinking involved reacting HA with polyethylene glycol diglycidyl ether (PEGDE)/itaconic acid (IT) (IPC scaffolds) or SA with calcium chloride (CC scaffolds) and the crosslinked gels (with/without BSA) freeze-dried. Selected optimized formulations were loaded with basic fibroblast growth factor (b-FGF) as medicated scaffold dressings. NMR and FTIR spectroscopies (crosslinking/component interactions), SEM (morphology), texture analysis (mechanical strength/adhesion), and exudate handling were used to characterize the physico-chemical properties of the scaffolds. Protein (BSA) release profiles, hemostasis, biocompatibility and wound closure were assessed using HPLC, whole blood and methyl thiazolyl tetrazolium (MTT) and scratch assays respectively. The CC SA:FCOL:HA scaffolds showed improved mechanical strength, porosity, water vapor transmission rate, retained structural integrity after absorbing 50% exudate and promoted cell proliferation. The IPC scaffolds showed enhanced structural integrity, excellent hemostasis, retained three times more exudate than non-crosslinked scaffolds and provided acceptable pore size for cell adhesion and proliferation. The results show potential of CC and IPC SA:FCOL:HA scaffolds as medicated dressings for delivering proteins to chronic wounds. The study’s significance lies in their potential use as multifunctional, multi-targeted and therapeutic dressings to overcome challenges with chronic wounds and use as delivery platforms for other therapeutic agents for chronic wound healing.

**Graphical Abstract:**

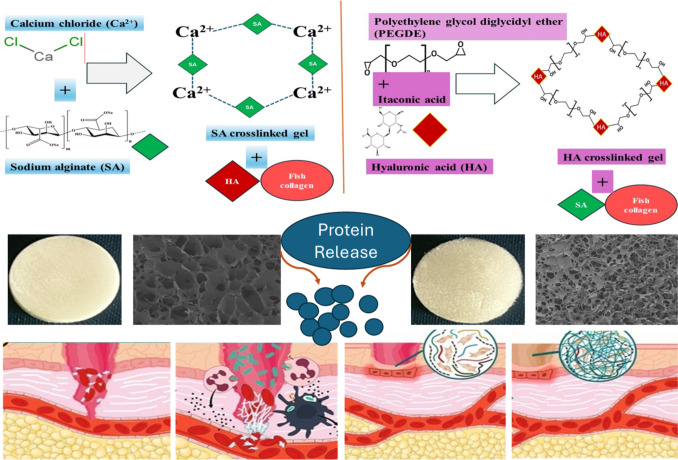

**Supplementary Information:**

The online version contains supplementary material available at 10.1007/s13346-024-01745-0.

## Introduction

The skin, a dynamic organ serving as the body's first line of defense, is crucial in maintaining physiological homeostasis and protecting against external threats. When compromised, it presents significant healthcare challenges, ranging from localized infections to life-threatening systemic complications such as infections, loss of tissue function, scar formation, morbidity, amputation and in extreme cases death [1]. The complex process of wound healing progresses through four interconnected phases: hemostasis, inflammation, re-epithelialization, angiogenesis, and extracellular matrix (ECM) remodeling, each orchestrated by a complex interplay of cellular and molecular mechanisms [2, 3].

Despite advancements in wound care, conventional treatments such as antiseptic cleansing, debridement, and traditional dressings often fall short in managing complex or chronic wounds [4]. Additionally, the prolonged duration required for wound healing, especially in chronic cases, is a major challenge, as these approaches may not fully optimize the normal healing stages. Furthermore, traditional dressings may focus on closing wounds without actively promoting optimum tissue regeneration (take no active part in wound healing) whilst also possessing poor exudate management, insufficient gas exchange and limited promotion of angiogenesis which can hinder the healing process [5]. A critical challenge in chronic wound management (such as diabetic foot ulcers) is the diminished presence of growth factors essential for tissue regeneration. While exogenous growth factor therapies have shown promise, their practical application is hindered by the need for high doses and frequent administration, highlighting the necessity for innovative delivery systems that ensure controlled release and sustained bioactivity at the wound site [6, 7].

Biopolymers have emerged as promising candidates for advanced wound dressings and drug delivery platforms, offering exceptional moisture retention, biocompatibility, and biodegradability. Among these, collagen and hyaluronic acid (HA), which are integral components of human skin are gaining attention for their potential to deliver growth factors directly to wound sites. While traditional collagen-based dressings from bovine and porcine sources have been widely used, they face limitations such as potential disease transmission (e.g. bovine spongiform encephalopathy) and religious restrictions. Fish collagen (FCOL) has emerged as a promising alternative, addressing these concerns while maintaining beneficial properties [8–10].

Numerous studies [11–13] have demonstrated the positive effects of collagen membranes and scaffolds on tissue healing through in vivo experiments and clinical trials. These studies showed that collagen accelerates skin regeneration and promotes cell attraction, proliferation, and re-epithelialization. Bohn et al. (2016) highlighted collagen's ability to reduce matrix metalloproteinase (MMP) levels by serving as a substrate for excessive proteases in chronic wounds [14]. Histological analyses from various investigators [13, 15–17] showed faster healing when using collagen from tilapia skin combined with bioactive glass nanofiber. Clinical trials also support the benefits of FCOL and alginate dressings on diabetic foot ulcers [18]. However, FCOL has poor mechanical properties due to its highly brittle nature and this can be overcome by forming composite matrices by blending it with other known biopolymers such as hyaluronic acid (HA) and sodium alginate (SA) [19]. HA is the primary component of the ECM that participates in several cell surface receptor interactions possessing immunosuppressive and antiangiogenic activity while being biocompatible, biodegradable and non-allergic. Due to its hydrophilic nature, HA absorbs large amounts of water, making it desirable for wound healing by managing wound exudate and maintaining a moist wound environment [20]. HA-based hydrogels enhance wound healing by promoting cell differentiation, migration, and angiogenesis [21, 22], however, HA's high solubility leads to poor mechanical stability and rapid degradation.

To enhance the mechanical stability, viscosity and solubility of biopolymers used in wound healing, researchers have explored the synergistic combination of FCOL, HA, and sodium alginate (SA) in composite scaffolds [19]. This approach addresses the limitations of each biopolymer while enhancing overall performance. Furthermore, researchers have developed more specific chemical and physical modifications for HA to improve its mechanical stability, viscosity, solubility, and degradation, to make it more suitable for wound healing application. Based on the functional groups present on the HA backbone, chemical modifications can be achieved by esterification of the carboxylic acid or by modification of the alcohol groups through ether bond formation [23]. This modification involves forming stable ether bonds (C–O–C) with crosslinking agents such as poly(ethylene) glycol diglycidyl ether (PEGDE), which improves HA's stability under physiological conditions [24]. These PEGDE-crosslinked HA hydrogels have shown promise in various applications, including ocular drug delivery [25]. On the other hand, SA, which is a natural polysaccharide presents unique properties for wound healing. Its ability to form hydrogels through ionic crosslinking with calcium ions makes it advantageous for creating strong, absorbent scaffolds suitable for bleeding wounds [26].

Building on these advancements, this study hypothesizes that freeze-dried composite scaffold dressings based on FCOL and HA, loaded with growth factor and mimicking the ECM, can function together to overcome the molecular hurdles encountered in chronic (non-healing) wounds. It aims to evaluate and compare the effect of two different crosslinking techniques on the two modifying polymers (HA and SA) present in composite FCOL, HA and SA scaffolds. It focuses on their ability to improve their functional performance including enhancing b-FGF encapsulation, control drug release whilst maintaining physical stability and prolonged retention at the wound site after application. Freeze-drying is a critical process in developing these advanced wound dressings. It creates a highly porous structure that enhances cell infiltration and nutrient diffusion while preserving the bioactivity of sensitive components such as growth factors. Upon rehydration, these dressings can rapidly absorb wound exudates and maintain an optimal moist environment conducive to healing. To the best of our knowledge, this is the first study using PEDGE crosslinked HA present within a composite scaffold comprising SA, FCOL and HA and comparing with corresponding calcium crosslinked SA within the composite scaffolds for potential delivery of growth factors to enhance the healing of chronic wounds.

## Materials and methods

### Materials

Fish collagen (FCOL) type I was purchased from Creative Enzymes Inc. (USA) and hyaluronic acid (HA) from Wisapple Biotech Co, Ltd (Beijing, China). Sodium alginate Pronatal^®^ LF 10/60 (SA 10/60) with G/M % ratios of 70/30 was donated by IMCD UK Limited, (Surrey, UK). Bovine serum albumin (BSA) and calcium chloride were obtained from Acros Organics (Geel, Belgium). Methyl thiazolyldiphenyl-tetrazolium bromide (MTT), trypan blue stain and sodium chloride were obtained from Fisher Scientific, (Loughborough, UK). D-mannitol (D-mann), poly (ethylene glycol) diglycidyl ether (PEGDE), itaconic acid (IT), basic fibroblast growth factor (b-FGF) and fetal bovine serum were purchased from Sigma Aldrich, (Gillingham, UK). Adult human primary epidermal keratinocytes (HEK) [PCS-200–011, ATCC], human dermal fibroblast (HDF) [PCS-200–011, ATCC], dermal cell basal medium [PCS-200–030, ATCC], keratinocytes growth kit [PCS-200–040, ATCC], keratinocytes growth kit [PCS-200–040, ATCC and Dulbecco’s Modified Eagle’s Medium (DMEM) [PCS-200–030, ATCC] were purchased from LGC Standards (Middlesex, UK).

### Preparation of composite gels

Non-crosslinked (NC) composite gels (control formulations) were prepared as previously reported [19] and the optimized formulations were selected for crosslinking. Briefly, the required amounts of SA, FCOL and HA (Table 1) were added slowly to the vortex of vigorously stirred deionized water (100 ml) at room temperature to avoid lump formation and stirring continued until a uniform gel solution was obtained. The resulting gels were left to stir gently overnight for complete hydration and left to stand until all air bubbles generated during stirring had disappeared.

**Table 1:** Composition of selected optimized blank 2% composite gels from preliminary development for crosslinking

Formulations	Polymer weight (g) ratio	Constituent amount (mg)		
		SA	FCOL	HA
SA: FCOL: HA	3:4:1	750	1000	250
SA: FCOL: HA	1:2:1	500	1000	500
SA: FCOL: HA	1:1:2	500	500	1000
SA: FCOL: HA	2:3:3	500	750	750
SA: FCOL: HA	1:2:5	250	500	1250
FCOL: HA	3:5	0	750	1250

#### Crosslinked gels

The preparation of hydrogels in this study involved two distinct crosslinking methods, each utilizing different types of water as solvents to optimize the gel formation process. For the calcium crosslinked (CC) gels, the process began with the dropwise addition of 10 ml of 2% w/v calcium chloride (CaCl_2_) solution to 2% w/v SA gels. This addition was performed using a syringe while maintaining continuous stirring. Following the initial crosslinking, FCOL and HA were gradually introduced into the crosslinked SA gel to produce the CC crosslinked composite gels. The mixture was then stirred further until a homogeneous and fully dispersed gel matrix was achieved. Throughout this process, regular distilled water was used as the solvent, as the calcium crosslinking method is robust and does not require ultra-pure water for effective gelation. In contrast, the preparation of IT-PEGDE crosslinked (IPC) gels employed twice distilled water as the solvent. This choice of higher purity water reflects the more complex and sensitive nature of the IT-PEGDE crosslinking process and helped ensure optimal gel formation and crosslinking efficiency by minimizing potential interference from impurities. For these gels, the amounts of each reagent (HA:IT:PEGDE) were carefully adjusted to achieve a 1:1:2 molar ratio. The mixture was stirred continuously for 2 h, after which the required amounts of FCOL and/or SA were added as specified in Table 1. The reaction was then allowed to proceed for 24 h under gentle stirring at room temperature, resulting in the formation of IPC composite gels.

#### Preparation of protein loaded gels

The formulations (Table 1) were employed for loading the model proteins bovine serum albumin (BSA) and basic fibroblast growth factor (b-FGF). BSA was used in preliminary experiments to help determine the final optimum formulations to use for b-FGF loading, due to the prohibitive costs of the latter. Initially, different amounts of BSA was incorporated into the optimized crosslinked gels after 24 h following the crosslinking reaction, with D-mannitol as cryoprotectant and pouring 3 g of gel into well plates resulting in a scaffold dressing containing 75—150 µg/g of BSA. Based on the appearance of the final scaffolds, formulations containing 75 µg/g of BSA were deemed optimum and taken forward. For b-FGF, the pH of each optimized gel was assessed using a pH meter to ensure an ideal pH range (7 – 9) to prevent denaturation during loading and the subsequent formulations prepared following a previously reported method [27] with slight modifications. Briefly, the b-FGF (50 µg) contained in a vial, was dissolved in 20 ml of double deionized water at room temperature to create a stock solution of 2.5 µg/ml. Subsequently, 1 ml aliquots of this stock solution were added to 100 ml of the crosslinked composite gels, resulting in a final b-FGF concentration of 0.025 µg/ml. 3 g of the final gel was poured into well plates for freeze drying with the final freeze-dried scaffold dressings containing 75 ng/g of b-FGF per sample.

#### Preparation of lyophilized scaffold dressings

3 g of the prepared gels (blank and protein loaded) were poured into each mold of twelve-well plates (22.1 mm diameter) and freeze-dried in a Virtis Advantage XL 70 freeze dryer (Biopharma Process Systems, Winchester UK), using an automated freeze-drying cycle as previously reported [19]. The samples were cooled from room temperature to 5 °C for 30 min, 5 °C to −5 °C for 60 min and −5 °C to −50 °C for 180 min. Furthermore, an annealing step was incorporated in the freezing step to enhance the pore size distribution by increasing the temperature from −55 °C to −25 °C for 2 h 30 min and cooling it back to −55 °C for 3 h. In the primary drying stage, a pressure of 50 mTorr was applied and the temperature was increased from −55 °C to −25 °C for 12 h and further increased from −25 °C to + 20 °C for 7 h. The same pressure was applied during secondary drying and the temperature was held at + 20 °C for 6 h to remove the remaining residual moisture. The lyophilized scaffold dressings were stored in a desiccator over silica gel to maintain a low moisture content that is ideal for maintaining protein stability until required for further analysis.

### Physico-chemical characterization

#### Texture analysis

A TA HD texture analyzer (Stable Microsystem Ltd., Surrey, UK) fitted with a 5 kg load cell and equipped with a Texture Exponent 32 software program was employed to characterize mechanical and adhesive properties of the gels and wafers as outlined below.

##### Gel back extrusion test

The texture analyzer, fitted with a specific back extrusion probe (Fig. S1) was used to determine the textural properties (consistency and firmness) of the gels. The extrusion pot (69 mm height, 50 mm internal diameter and 60 mm outer diameter) was filled with gel and compressed to a depth of 45 mm using a disc shaped probe (40 mm diameter) attached to the plunger at a speed of 1 mm/s. As the compression progressed, a positive force–time curve was generated by the instrument’s Exponent software program. Once the trigger force of 0.05 N was reached, the disc plunger began detachment from the gel and returned to its starting position. The maximum positive (peak) force on the graph represents gel firmness whilst the positive area indicates the sample consistency. As the probe returned to the starting position, a negative area was generated from the back extrusion representing the cohesiveness and resistance of the sample to the detachment from the disc (see Fig. S1). The experiment was performed in triplicate (*n* = 3) for each sample.

##### Mechanical strength (hardness)

Resistance to compressive deformation (hardness) representing the mechanical strength of the scaffold dressings was studied with the texture analyzer. The investigations were performed in compression mode using a 6 mm (P/6) cylindrical stainless-steel probe (Stable Microsystem Ltd.) to investigate the effect of total polymer content and composition of the polymers within the formulation and type of crosslinking. The scaffold dressings (*n* = 3) were compressed at three different positions on both sides to a depth of 2 mm, using a trigger force of 0.001 N, at a speed of 1 mm/s and a 10 mm return distance.

##### Adhesion

The same texture analyzer above was used to study the in vitro wound adhesive performance of the scaffold dressings. The dressings (*n* = 3) were attached to a 35 mm cylindrical stainless-steel adhesive probe (P/35) with the aid of double-sided adhesive tape. A simulated wound surface was achieved by allowing gelatine (6.675%) solution to set and then equilibrated with 500 µl of simulated wound fluid (SWF). The SWF contained 2% (w/w) BSA, 0.02 M CaCl_2_, 0.8 M sodium chloride in deionized water at pH 7.4. The probe attached to the scaffold dressings approached the model wound substrate in tensile mode at a pre-test speed of 0.5 mm/sec; 60 s contact time and applied force of 1 N followed by detachment at a test speed of 0.5 mm, post-test speed of 1 mm/sec, a trigger force of 0.05 N and 10 mm return distance. The adhesive profiles of the scaffold dressings were determined by the peak adhesive force (PAF) required to disengage the scaffold dressings from the gelatine surface and represents stickiness, the total work of adhesion (WOA) signified by the area under the force versus distance curve, and the cohesiveness (the distance travelled by the scaffold dressings till detached) and calculated with the help of the Texture Exponent software.

##### Gel strength of the swollen scaffolds

Finally, the texture analyzer was used to evaluate the dynamics of the gel layer formed upon hydration of the scaffolds in SWF to mimic what happens when the scaffolds are placed on an exuding wound surface. The swollen formulation was removed from the SWF and the experiment was performed in compression mode and assessed using a cylindrical steel probe (2 mm diameter) using pre-test and test speeds of 1 mm/s and a trigger force of 0.001 N. The total work of penetration representing the gel strength in response to the resistance generated during the probe displacement was obtained equation from Eq. 1.

1 $$\text{The total work done through penetation}=\text{Wt}=\int \text{FdD}$$

where W is work done at any given time t, F = force applied, d is the diameter of the probe and D is the distance travelled.

### Analytical characterization

#### Scanning electron microscopy (SEM)

The surface morphology of the lyophilized scaffold dressings was analyzed by a (Hitachi SU8000, HI-0210–0005, Hitachi High-Technologies; Germany) scanning electron microscope at accelerating voltage of 1 kV and working distances of 8500 and 11,400 mm. A thin piece of scaffold was fixed onto an aluminium stub using double-sided carbon tape and gold coated, prior to acquisition of the micrographs and images acquired at different magnifications. The pore size and wall thickness of both top and bottom sides of the scaffold dressings were measured and compared between the different formulations.

#### X-ray diffraction (XRD)

The physical form of the scaffold dressings and the starting materials were analyzed using a D8 Advance X-ray diffractometer (Burker AXS GmbH, Karlsure, Germany) with an exit slit of 0.6 mm and sample rotation of 30 rpm in transmission mode. The samples were compressed using a pair of clean compression glasses to a height of 0.5 mm and mounted onto the sample holder while powders for the starting materials were compressed and mounted using Mylar. The transmission diffractograms were acquired using the DIFFRAC plus XRD commander with 2θ range of 5°—45°, step size of 0.02, scan speed of 0.4 s. The voltage and current were set at 40 kV and 40 mA respectively with Cu Kα radiation.

#### Fourier transform infrared spectroscopy (FTIR)

To characterize the interactions between the three polymers within the composite scaffolds and between these polymers and protein drugs, a FTIR spectrophotometer (Thermo Nicolet, Thermo scientific, UK) combined with ZnSe attenuated total reflectance (ATR) crystal accessory was used. A small quantity of the scaffold dressing and starting material were placed on the ATR crystal and compressed with the pressure clamp to allow proper contact between the material and the ATR crystal. The spectra for the samples (scaffold and starting materials) were collected at a 4 cm^−1^ resolution between a wavenumber range of 400–4000 cm^−1^.

#### NMR spectroscopy

NMR spectroscopy was used to analyze the blank and b-FGF loaded IPC scaffold dressings by capturing 1H-13C CP MAS NMR spectra using a JNM-ECZ400R/S1spectrometer. The analyses involved the use of ZrO rotors spinning at 4.5 kHz and spectroscopic data collected with a 4-s relaxation delay, a 2 ms contact time, and acquisition time of 0.13 s and 2000 scans. The chemical shifts were determined in ppm, referenced to tetramethylsilane (TMS).

### Exudate handling properties

#### Swelling

The hydration and degree of swelling of the scaffold dressings were performed as previously reported [28]. Briefly, the scaffolds were accurately weighed and immersed in SWF (pH 7.4) at room temperature. The weight change was recorded every 15 min for the first hour and then every hour for 5 h. Before weighing, the hydrated samples were blotted gently with tissue paper to remove excess SWF on the surface. The swelling capacity (*Is*) was calculated (*n* = 3) using Eq. 2, where *Ws* and *Wi* are the weights of the hydrated and initial dry weight of the scaffold dressings respectively.

2 $$Is \left(\text{\%}\right)= \frac{Ws-Wi}{Wi}\times 100$$

#### Porosity

The solvent displacement method was used to investigate the porosity of lyophilized dressings as previously reported [29]. To determine the geometrical dimensions (thickness and diameter) and to calculate the total pore volume of formulations, a digital Vernier caliper was used. Samples (*n* = 3) were weighed (W_0_) and immersed in 10 ml of absolute ethanol for 3 h to allow complete saturation, with the void space in the scaffold dressings displaced by ethanol. Eventually, the samples were carefully removed from the solvent, quickly blotted with tissue paper for 15 s on each side and immediately weighed (W_t_) to avoid the loss of ethanol. Equation 3 was used to calculate the % porosity of the dressings.

3 $$Porosity\left(\%\right)=\left.\left(Wt-{W}_{0}\right)/\left(\rho eth V\right)\times 100\right)$$

ρeth: density of ethanol = 0.789 g/cm^3^.

#### Water absorption (AW) and equilibrium water content (EWC)

To investigate the maximum water uptake (AW) and water holding capacities (EWC), the formulations (*n* = 3) were incubated in 10 ml of SWF at 37 °C for 24 h. The initial weight (Wi) of the scaffold dressings before incubation was recorded whilst the swollen weight (Ws) after 24 h was recorded after removing the excess fluid by carefully blotting with tissue paper. The percentage AW and EWC were calculated using Eqs. 4 and 5 respectively.

4 $$\text{AW }\left(\text{\%}\right)=\frac{\text{Ws}-\text{Wi}}{\text{Wi}}\times 100$$

5 $$\text{EWC }\left(\text{\%}\right)= \frac{\text{Ws}-\text{Wi}}{\text{Ws}}\times 100$$

#### Water vapor transmission rate (WVTR)

To determine the moisture permeability of the formulations, the WVTR was measured based on a well-established method [28]. Briefly, the dressing was mounted onto the mouth of a cylindrical tube (16.66 mm diameter) containing 8 ml SWF which allowed an 8 mm air gap between the dressing and the SWF surface. The whole setup was weighed and then placed into a 37 °C incubator for 24 h. The experiment was performed in triplicate (*n* = 3) for each formulation and the amount of water evaporated through the dressing per m^2^ over 24 h was calculated using Eq. 6.

6 $$\text{WVTR}=\frac{\text{Wi}-\text{Wt}}{\text{A}}\times {10}^{6}/{\text{m}}^{2}{\text{day}}^{-1}$$

where Wt is the swollen weight at 24 h and Wi is the initial weight before incubation in SWF.

#### Evaporative water loss (EWL)

The formulations previously incubated for AW and EWC experiments were taken out of the SWF, drained and then dried in an oven for 24 h at 37 °C. The weight was noted at regular intervals, and the EWL (*n* = 3) was calculated with Eq. (7).

7 $$EWL (\%) = [(Wt/Wi)] \times 100$$

*W*_*i*_ is the initial weight after 24 h incubation in SWF and *W*_*t*_ is the weight after time *t*, respectively.

### In vitro* BSA dissolution studies*

The drug dissolution studies were performed using a diffusion cell developed in-house containing 20 ml SWF (without BSA) as dissolution medium. The dissolution medium was filled up to the wire mesh allowing the bottom of the test sample to just be in touch with the medium. Whole scaffold dressings (*n* = 3) were placed on the wire mesh and the complete set up placed in an ice bath to mitigate protein degradation with constant stirring (500 rpm). At different time intervals, 1 ml of the dissolution medium was withdrawn and replaced with fresh medium. The withdrawn dissolution medium was analyzed for protein using HPLC and the equation of a calibration graph was used to calculate the concentration of drug released at each time point up to 72 h.

The release kinetics of BSA from polymeric scaffold dressings were analyzed using five mathematical models: Hixson-Crowell, Higuchi, zero-order, first-order, and Korsmeyer-Peppas (Eqs. 8, 9, 10, 11 and 12 respectively). These models describe different aspects of drug release [29]:

- 1. Hixson-Crowell:
  8 $${\text{m}}_{0}^{1/3}-{\text{m}}_{\text{left}}^\frac{1}{3}= {\text{K}}_{\text{H}-\text{C}}\text{t}$$

(For systems with changing surface area and particle diameter)

- 2. Higuchi:
  9 $${\text{Q}}_{\text{t}}= {\text{K}}_{\text{H }}{\text{t}}^{0.5}$$

(Fickian diffusion-based release)

- 3. Zero-order:
  10 $${\text{Q}}_{\text{t}}-{\text{Q}}_{0}={\text{K}}_{0}\text{t}$$

(Concentration-independent release)

- 4. First-order:
  11 $$\text{ln}\left({\text{m}}_{0}-{\text{m}}_{\text{t}}\right)=\text{ln}\left({\text{m}}_{0}\right)-{\text{K}}_{1}\text{t}$$

(Concentration-dependent release)

- 5. Korsmeyer-Peppas:
  12 $$\text{log }\left(\frac{{\text{Q}}_{\text{t}}}{{\text{Q}}_{\infty }}\right)={\text{logK}}_{\text{k}-\text{p}}+\text{nlogt}$$

(For unknown or multiple release mechanisms)

Where m_0_, m_t_, m_left_ represent initial, time t, and remaining drug amounts; Q_t_, Q_0_, Q _∞_ are amounts released at time t, initially, and at infinite time respectively; K values are respective rate constants; and n is the release exponent. The best-fit model was determined by comparing the correlation coefficients (R^2^) of these equations after fitting of the dissolution data.

### Stability evaluation

Representative BSA loaded scaffold dressings were evaluated for stability under ICH conditions for drug products intended for storage in a refrigerator. Samples were stored in a refrigerator at 5 °C (for long-term study over 12 months) and in a humidity chamber with 60% ± 5% relative humidity (RH) maintained using magnesium nitrate salt solution at 25 °C (for accelerated stability studies over 6 months). Due to cost reasons, growth factor loaded scaffolds were not assessed for long term stability. The amount of BSA at time zero and monthly after storage at different conditions was determined with an Agilent 1100 series size-exclusive high performance liquid chromatography (SE-HPLC) analysis system as described above. Samples were dissolved in 20 ml of 0.01 M PBS at pH 7.4, filtered (0.45 µm membrane, Minisart Biotech) and the BSA concentration at each time point was estimated from the calibration curve.

### Biological characterization of the scaffold dressings

#### Cell viability (MTT) assay

The effect of scaffold dressings on human dermal fibroblasts (HDFs) and primary epidermal keratinocyte (PEK) cells was assessed using an MTT assay via indirect contact as previously reported [31]. Scaffold dressings were UV-sterilized overnight, then incubated in complete medium (37 °C, 5% CO_2_). The supernatant was filtered (0.2 µm) to prevent viscous gel formation. Cells (1 × 10^5^ cells/ml) were cultured in 96-well plates overnight and after 24 h, the media was replaced with 100 µl of filtered scaffold-conditioned medium. Plates were incubated for 24 h and 48 h, then treated with 10 µl MTT reagent and incubated for 4 h. The resulting formazan crystals were dissolved, and absorbance was measured at 492 nm. Experiments were conducted in triplicate (*n* = 9), with Triton-X as a positive control. Cell viability was calculated using Eq. 13. Where At, Ab and Ac are the absorbance values of tested samples, media only, and negative control (untreated cells) respectively.

13 $$\text{Cell viability }\left(\text{\%}\right)= \frac{\text{At}-\text{Ab}}{\text{Ac}-\text{Ab}}*100$$

#### In vitro* blood clotting assay*

The influence of scaffold dressings on blood clotting was assessed using whole human blood (with approval from the University of Greenwich Research and Ethics Board). Sterile samples were prewarmed in Petri dishes at 37 °C for 5 min (Promogran™ served as a control). Anti-coagulated human blood within an acid citrate dextrose tube (300 µl) was applied to the scaffold surface, and coagulation was initiated by adding 25 µl of 0.2 M CaCl_2_ and incubated for 10 min at 37 °C. Thereafter the non-trapped erythrocytes were hemolyzed with 5 ml distilled water and hemoglobin absorbance measured at 542 nm with the absorbance of 300 µl whole blood in 5 ml distilled water serving as a reference (100%). Experiments were performed in triplicate (*n* = 3) and the blood clotting index (BCI) was calculated using Eq. 14. Where Asc and Awb are the absorbance of treated scaffolds and whole blood reference respectively. A high BCI indicates decreased blood clotting, while a low BCI suggests enhanced clotting potential.

14 $$\text{Blood clotting index }\left(\text{BCI}\right)= \frac{\text{Asc of blood in contact with sample}}{\text{Awb of whole blood in water }} \times 100$$

#### In vitro* wound healing (scratch) assay*

Human PEK cells were seeded at 5 × 10^4^ cells per well in 24-well plates, and incubated until they formed a monolayer with 80–85% confluence. Wound gaps were uniformly created in the cell monolayers using a 200 µl micropipette tip, standardized to the tip's outer diameter (775 µm). Following the creation of the wound, wells were washed with Dulbecco’s phosphate-buffered saline to remove detached cells. Treatment involved application of the various formulations and controls (Triton-X-100 for positive control, untreated wells for negative control). Cell migration was tracked using a Nikon Eclipse Ti-U fluorescence microscope, capturing images from day 0 to 49 at low magnification (4 × lens). Wound closure was quantified using NIS-Elements software, based on the wound length measured initially (D0) and at subsequent time points (Dt), according to Eq. 15 [32].

15 $$\text{Wound closure }\left(\text{\%}\right)= \frac{\text{D}0-\text{Dt}}{\text{D}0}*100$$

### Statistical analysis

Statistical analysis was performed using one-way ANOVA followed by Sidak’s multiple comparisons tests where appropriate. The differences were considered significant when *p* ≤ 0.05.

## Results and discussion

### Visual evaluation of freeze-dried scaffolds

**Fig. 1:**
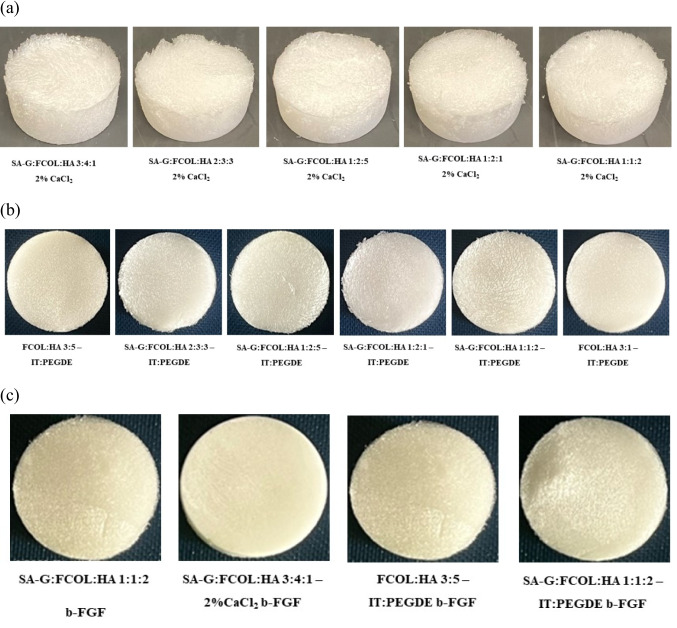
Shows representative digital photographs of (**a**) blank composite scaffolds prepared from 2% SA-G, FCOL and HA gels crosslinked with 2% CaCl_2_. (**b**) blank scaffolds crosslinked with IT-PEGDE, (**c**) selected optimized scaffolds (plain and crosslinked) loaded with b-FGF

The criteria for evaluating scaffolds were softness, no shrinkage, ease of removal from well plates and tolerability to minor external forces such as handling. Though the gels for preparing the CC scaffolds which contained higher amounts of HA, such as SA-G:FCOL:HA 1:2:5 were more viscous and difficult to pour into wells, the resulting scaffolds were elegant and easy to handle (Fig. 1a).

All the IPC scaffolds (Fig. 1b) were soft and flexible to handle, but strong and not brittle. They also showed a smooth surface appearance apart from FCOL:HA 3:5 IT-PEGDE BSA scaffolds which showed a patchy surface with rough edges. This was because the gels of this formulation were highly viscous and while pouring into the well plates bubbles were generated that upon freeze drying resulted in rough, uneven surfaces and edges. The extended gentle stirring time, particularly for the IT-PEGDE crosslinked (IPC) gels helped to reduce bubble formation. However, all prepared gels were left to stand overnight which removed all residual air bubbles without the need to use special procedures such as vacuum removal.

The smooth, flexible but strong scaffolds produced showed that the gels obtained prior to freeze-drying were homogeneous and of uniform consistency and confirmed the successful crosslinking of the composite polymer gels by IT-PEGDE. Like the CC scaffolds, none of the IPC scaffolds were eliminated at this stage and further analytical characterization tests were performed as part of the formulation development and optimization. In comparing between the two crosslinking methods, the IPC scaffolds were more elegant in appearance with smoother surfaces and edges as well as being firmer and stronger upon handling, than the corresponding CC scaffolds. This suggests that crosslinking of HA with IT-PEGDE produced stronger hydrogels, than crosslinking of the SA-G with CaCl_2_. All the b-FGF loaded formulations (Fig. 1c) were easy to handle and non-brittle apart from FCOL:HA 3:5 IT-PEGDE b-FGF scaffold, which was slightly brittle, as was the case for the corresponding BSA loaded formulation. This will suggest that the replacement of BSA with b-FGF did not significantly alter the physical properties (by handling), which is not surprising, since the dose of the growth factor was 1000 times lower than that of BSA. All other visual features were similar for all the optimized scaffolds.

### Texture analyses (TA)

#### Back extrusion

Scaffolds are converted into gels after encountering wound exudate and should have a robust hydrated structure to withstand external forces such as movement and continuous washing with exudate, while also providing intimate and sustainable contact with the wounded tissue. For designing an optimal scaffold dressing with prolonged retention time at the wound site, it is important to maintain a balance between the adhesiveness and cohesiveness of the gel and back extrusion test can help to determine these properties. The back extrusion test determined the firmness (N), the maximum positive force, consistency; the maximum positive area (N.sec) and cohesiveness; the maximum negative force (N) and the maximum negative area (N.sec) representing the viscosity and the work done to overcome the negative force of resistance to flow off the disk for all the gels tested. The results of back extrusion test on the blank and BSA loaded CC and IPC gels are presented in Table S1a&b with further exposition provided in section S1.1 of the supplementary information.

#### Hardness

Polymeric scaffold dressings with three-dimensional porous structures are crucial for tissue engineering to promote cell ingrowth while maintaining the transport of oxygen and nutrients. Mechanical strength is also considered to overcome the rapid degradation by enzymes and environmental changes, helping to prevent infections and provide a physiological environment that facilitates cell adhesion, proliferation and differentiation [33]. However, achieving an ideal balance between porosity and mechanical strength is an essential but challenging aspect when characterizing wound dressings. If the scaffold dressings are too hard, it could cause some damage to newly formed tissue, prolonging the inflammation stage and increasing the wound healing time. Conversely, if they are too soft, they easily fall apart during handling or disintegrate rapidly in the presence of wound exudate, and therefore require frequent dressing changes, with associated pain and inconvenience [34]. Ideally, for adequate adhesion to the wound, the wound dressing should withstand the applied force [35, 36] and possess appropriate mechanical properties to accommodate different types of wounds.

From the hardness profiles of blank CC scaffold dressings shown in Fig. S3i, it was evident that the peak resistance force was within the accepted range (2N- 4N). Although the hardness (resistance to compression) values for some scaffold dressings were above 4N, these scaffold dressings were not deemed brittle when handled manually. The hardness of the top of the scaffold dressings were marginally higher than the bottom, however, the differences were statistically not significant (*p* > 0.05). This means the bottom of the scaffold dressings was softer than the top of the dressing and could be the ideal side to be in direct contact with newly formed tissue on a healing wound. Furthermore, formulations with higher concentrations of SA-G showed increased resistance to compression, which could be explained by the interaction of Ca^2+^ ions with the guluronic moieties of the SA-G with the divalent cations forming ionic bridges between the polymer chains [37].

The hardness profiles of the IPC scaffolds are shown in Fig. S3ii. It was evident that the crosslinking with IT:PEGDE (confirmed by gel permeation chromatography-Fig. S2) produced rigid scaffolds that were within the acceptable range [38]. Although the SA-G:FCOL: HA 1:2:5 and SA-G:FCOL: HA 1:2:5 IPC scaffolds exhibited hardness values above 4 N, these scaffolds were also not brittle and were easy to handle. Studies have shown that soft 3D matrices stimulate faster cell migration than stiff ones. It is hypothesized that cells sense their way to softer environments, which encourages cell migration [39].

**Fig. 2:**
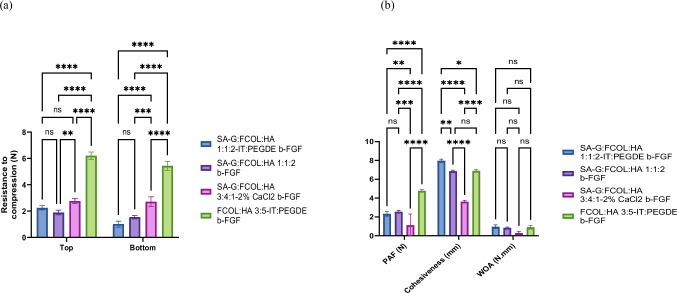
(**a**) Hardness (resistance to compression) and (**b**) adhesive (PAF, WOA and cohesiveness) profiles of optimized scaffolds loaded with b-FGF *(n* = *3* ± *SD)* showing the comparisons between different selected optimized formulations

Figure 2a shows the average resistance to compressive deformation (hardness) of the growth factor (b-FGF) loaded scaffolds. The hardness of the scaffolds was within the expected range with the exception of FCOL:HA 3:5-IT:PEGDE b-FGF which had a high value of (6.2 N) and SA-G:FCOL:HA 3:4:1–2% CaCl_2_ b-FGF, SA-G:FCOL:HA 1:1:2-IT:PEGDE b-FGF and SA-G:FCOL:HA 1:1:2 b-FGF which had low values of 2.7N, 2.2N and 1.9N respectively. From the results achieved it was evident that crosslinking enhanced the mechanical properties of the scaffolds. Furthermore, scaffolds with higher concentrations of HA showed higher hardness values due to the increase in crosslinking. The FCOL:HA 3:5-IT:PEGDE b-FGF scaffolds were brittle as seen in the digital images presented in Fig. 1c and this is confirmed by the high hardness values of these scaffolds. These composite scaffolds appeared to show a denser more porous structure compared to the other b-FGF loaded scaffolds hence a higher force was required to reach the target depth of penetration. In contrast to the BSA loaded scaffolds the hardness of the scaffolds decreased after loading with b-FGF except for IPC FCOL:HA 3:5 scaffold which increased significantly. This indicates possible interaction between the b-FGF, and the scaffold components as evidenced by the sharper peaks observed in the FTIR spectrum. However, it should also be noted that this outlier scaffold formulation contained no SA-G and therefore higher amounts of FCOL which is naturally brittle than the SA-G and HA and this may also be a determining factor in its mechanical behavior.

#### Adhesion

The removal of an adhesive dressing from a wound surface presents the risk of damaging the stratum corneum and regenerating epithelium. The pain associated with removing the wound dressing is also a challenging issue for patients and medical personnel as this results in non-adherence with consequential increases in cost and patient morbidity. Appropriate adhesive performance of dressings is important not only for easy application and retention but also in preventing microbial penetration and allowing exchange of nutrients and oxygen. It is worth mentioning that the adhesive profile is influenced by the physiochemical properties of the scaffold dressings such as the pore size distribution, hardness, and the subsequent hydration capacity [31].

Figure S4i shows the adhesive profiles of the CC scaffold dressings. Scaffold dressings with higher concentrations of HA (SA-G:FCOL:HA 1:2:5) exhibited higher PAF (2.79 ± 0.1 N) compared to (SA-G:FCOL:HA 3:4:1) (0.81 ± 0.1 N). The results confirmed an increasing trend in PAF that was proportional to the concentration of HA and a similar trend was observed in WOA and cohesiveness. The differences in the adhesion parameters studied could be attributed to the hydrophilic nature of HA which promotes the interaction between the scaffold matrix and the simulated wound environment due to the faster initial hydration. In contrast the lower adhesive profile of scaffold dressings with higher concentration of SA-G could be explained by the resistance and slower interpenetration of water and ion exchange due to the formation of crosslinks between SA-G and Ca^2+^ ions. Incorporation of BSA also decreased the adhesion of the scaffold dressings marginally, and this could be due to stabilized structure of the scaffold dressings and decreased porosity, resulting in less efficient and slower penetration of wound fluid into the scaffold dressings cavities [40]. However, the differences between the adhesive profiles of the blank and the BSA loaded scaffold dressings were statistically not significant (*p* > 0.05).

The adhesive profiles (Fig. S4ii) of the IPC scaffold dressings revealed marked changes in the adhesion behavior compared to the NC and CC scaffold dressings. As was the case with the back extrusion results, the PAF generally increased with increasing concentrations of HA with a PAF value of 3.85 ± 0.135 N for FCOL:HA 3:5-IT:PEGDE, > FCOL:HA1:3-IT:PEGDE (3.50 ± 0.15 N > SA-G:FCOL:HA 2:3:3-IT:PEGDE (2.93 ± 0.53 N) > (SA-G:FCOL:HA1:1:2-IT:PEGDE) (2.43 ± 0.13 N) > SA-G:FCOL:HA 1:2:1-IT:PEGDE (2.34 ± 0.62 N). However, SA-G:FCOL:HA 1:2:5-IT:PEGDE scaffold dressings with a PAF of 2.53 ± 0.22 N did not follow this trend. This could be due to the high crosslinking density of (SA-G:FCOL:HA 1:2:5-IT:PEGDE) scaffold dressings that resulted in higher polymer chain entanglement, making them more resistant to fluid penetration and ionic bond formation with the gelatine substrate. Interestingly, the WOA representing the amount of energy required to detach the scaffold dressings from the gelatine substrate followed a similar trend to PAF based on HA concentration.

In contrast to the CC scaffolds where insignificant decrease occurred, loading BSA caused a significant (*p* < 0.05) decrease in the adhesiveness of the IPC scaffolds. It is evident that by adding IT-PEGDE, the ionic groups increased hence these scaffold dressings had many -COO- that could cause increased reaction between the carboxylic group and the protein. The isoelectric point of BSA is between pH 4.5 and 4.8 where net charge of the molecule is zero, hence the amount of negatively charged groups on the BSA molecule -COO- (carboxylic) increases at higher pH whilst the positively charged -NH^2+^ groups increase at lower pH. The decreased adhesion and cohesion of BSA loaded scaffold dressings could be caused by the electrostatic repulsion between the -COO- ions present from IT-PEGDE and the -COO- ions on the BSA molecule as the pH of the media used to equilibrate gelatine substrate had a pH of 7.4 [40, 41].

Figure 2b shows the adhesive profiles (PAF, WOA and cohesiveness) for the b-FGF loaded scaffolds. All the b-FGF loaded scaffolds showed high cohesiveness values above 6.5 mm except for the CC SA-G:FCOL:HA 3:4:1 scaffold which showed a significant decrease (*p* < 0.05) to 3.6 mm. The PAF data also showed the CC SA-G:FCOL:HA 3:4:1 scaffold with the lowest PAF values. The WOA values were, on the other hand similar for the first three formulations, however, once again, the CC SA-G:FCOL:HA 3:4:1 scaffold showed the lowest WOA values. Therefore, it will appear that the lower the HA content, the less adhesive the formulations were, which is interesting as HA is a known sticky (adhesive polymer) that helps maintain skin integrity, flexibility and hydration, due to its humectant properties.

Comparing between the different formulations, the PAF of the FCOL:HA 3:5-IT:PEGDE scaffolds increased from 2.70 N (BSA loaded) to 4.76 N (b-FGF loaded), while SA-G:FCOL:HA 1:1:2-IT:PEGDE decreased from 2.97 N (BSA loaded) to 2.31 N (b-FGF loaded). In addition, the PAF of the SA-G:FCOL:HA 1:1:2 CC scaffold decreased from 1.89 N (BSA loaded) to 1.10 N (b-FGF loaded). Finally, the PAF of the NC SA-G:FCOL:HA 1:1:2 decreased from 2.61 N (BSA loaded) to 2.54 N (b-FGF loaded). Therefore, as observed in the hardness results, the FCOL:HA 3:5 scaffolds possessed the best adhesive profiles so far.

### Scanning *electron* microscopy (SEM)

**Fig. 3:**
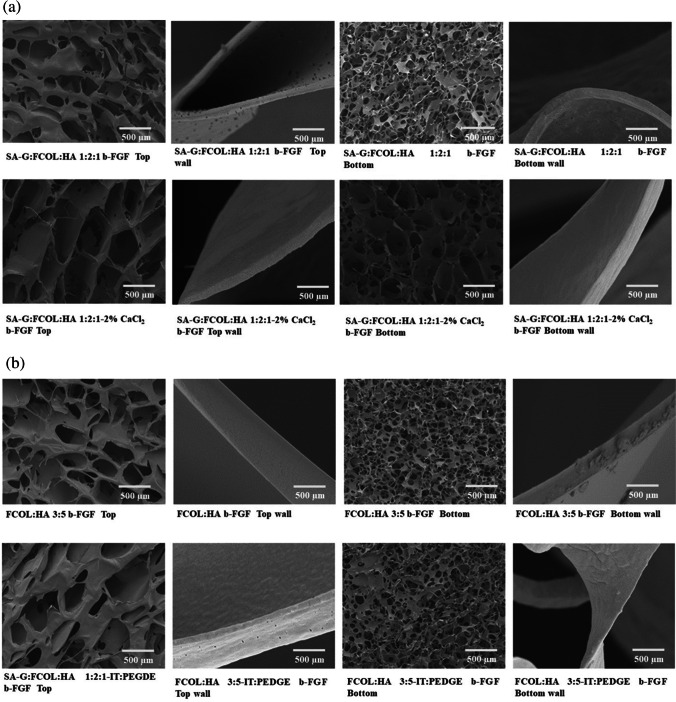
Shows representative optimized NC, CC and IPC scaffolds (SA-G:FCOL:HA 1:1:2) loaded with b-FGF

The comparison of NC, CC and IPC scaffold dressings by SEM revealed distinct morphological differences with implications for their use in biomedical applications as shown in Fig. 3 and Fig. S5 (supplementary data). The CC scaffolds were characterized by an irregular, interconnected porous structure (Fig. S5a&b) with pore and wall thickness influenced by the polymer concentration, particularly SA and HA. Higher SA concentrations resulted in sheet-like structures, while higher HA levels produced thicker walls. Further, these scaffolds exhibited varying porosity between the top and bottom parts. In contrast, the IPC scaffolds, (Fig. S5c&d), displayed a porous structure with an open, interconnected network of pores. The pore sizes and distributions varied between the top and bottom, affected by the freeze-drying conditions and polymer concentration. Higher HA concentrations led to less porous surfaces, yet the overall pore size remained unchanged. BSA loading resulted in decreased porosity, indicating different interactions with the polymeric matrix compared to the CC scaffolds, however, the loading of b-FGF did not cause any major changes in the pore distribution or pore sizes given the low doses (Fig. 3).

The significance of scaffold pore size extends beyond cell growth considerations, particularly for dressings designed primarily for drug delivery. In this context, the pore characteristics play a pivotal role in determining drug loading capacity, matrix hydration and swelling, release kinetics, and overall delivery efficiency. The interplay between macroporosity and microporosity in these scaffolds offers intriguing possibilities for achieving different drug delivery and release profiles. Macropores, typically larger than 50 μm, contribute significantly to the scaffold's drug loading capacity and influence the initial burst release upon hydration. They also enhance fluid absorption, which can be beneficial in managing wound exudates. Conversely, micropores, smaller than 50 μm, are crucial for sustained drug release and provide an increased surface area for drug adsorption, potentially allowing for more controlled and prolonged delivery [42, 43]. For the CC scaffolds, SA-G:FCOL:HA 3:4:1 formulation stands out due to its unique sheet-like structures and thin, staggered flake-like structures on the underside (bottom), offering a balance between pore distribution and pore wall network structure that could be beneficial for specific wound healing applications. Interestingly the IPC scaffolds produced both macro pores (top of the scaffold ranging from 100–300 µm) and micro pores (bottom of the scaffold ranging from 50–150 µm) (Fig. S5c&d). This gives the scaffolds, especially the SA-G:FCOL:HA 1:1:2 formulation, the potential to be applied to the wound surface on either side depending on the wound requirement. This dual pore size distribution could provide advantages in wound care, catering to both nutrient exchange and cell attachment needs.

### X-ray diffraction (XRD)

Pawar and co-workers noted that the physical form (crystalline or amorphous) of polymeric dressings affects many properties such as water uptake, biodegradability, and bioadhesion [44], with amorphous structures having a less ordered molecular lattice exhibiting improved characteristics such as exudate absorption and prolonged retention of the dressing at the wound site. The XRD analysis of the raw materials and the scaffold dressings (Fig. S6) showed differences between the CC and IPC crosslinked formulations that are essential in understanding their implications in wound healing applications. In the case of raw materials, both HA and FCOL displayed a typical amorphous pattern, while SA showed semi-crystalline nature confirmed by the presence of peaks at 13.9° and 21.6° and a halo peak at 39.5° 2θ. The disappearance of these peaks in the CC and IPC scaffolds confirmed the reduction in crystallinity indicating that crosslinking induced changes to the physical structures of the polymers from semi-crystalline to amorphous. These structural changes have direct implications for the scaffolds' functionality in wound healing, particularly in terms of water uptake, biodegradability, bioadhesion, and bioavailability. The XRD diffractograms of selected b-FGF loaded scaffolds are shown in Fig. S6d, with no sharp peaks present in any of the formulations, similar to the BSA loaded scaffolds. This suggests that all the growth factor loaded formulations were amorphous in nature and that the loaded b-FGF was molecularly dispersed within the scaffolds, which is expected to aid in rapid hydration and swelling in the presence of SWF. However, it is also possible that the b-FGF was not detectable by the XRD due to the very low amounts present.

### Fourier transform infrared spectroscopy (FTIR)

FTIR spectroscopy allows the recognition of the skeleton of the crosslinked polymeric network and confirms the region of crosslinking in the chemical structure through changes in peak position (s) and intensity as well as appearance / disappearance of specific functional group peaks. The FTIR spectra for SA-G:FCOL:HA scaffold dressings, showed several key changes post crosslinking when compared to the pure starting materials (Fig. S7a-e). Figures S7f&g show that for the CC scaffolds, the shift in the amide I bond from 1600 cm^−1^ to 1606 cm^−1^ is indicative of alterations in the secondary structure of FCOL, suggesting an increase in α-helix content. This alteration, caused by the crosslinking interrupting the interactions between SA-G and FCOL, could have significant implications for the mechanical and biological properties of the scaffolds. Similarly, the shift in the amide II band from 1400 cm^−1^ to 1556 cm^−1^ suggests enhanced hydrogen bonding between the amine groups of FCOL and carboxylic groups of SA-G or HA, potentially impacting the scaffold's stability [45]. The changes observed in the SA-G component further underscore the impact of crosslinking. The shift in the C = O peak from 1409 cm^-1^ to 1395 cm^−1^ after CaCl_2_ treatment suggests ionic bonding between Ca^2+^ and the COOH groups of SA, a modification that may contribute to the scaffold's enhanced structural integrity.

Figure S7h-i shows the characteristic peaks for the effect of crosslinking on HA both alone and within the composite SA-G:FCOL:HA-IT:PEGDE scaffolds. The FTIR spectrum of the pure PEGDE (Fig. S7a) showed characteristic peaks at 3066 cm^−1^ and 2869 cm^−1^ (attributed to CH_2_ and CH stretching respectively) and 1459 cm^−1^ due to scissoring of CH_2_ and 1349 cm^−1^ (bending of CH). In addition, a strong absorption band at 1095 cm^−1^ was due to C-O and C–C stretching while the bands at 913 cm^−1^ and 840 cm^−1^ were assigned to the stretching vibration of the epoxide in PEGDE. The FTIR peaks in the 1000—3300 cm^−1^ region for the NC HA have previously been discussed [19], however, a band present at 598 cm^−1^ is related to carbohydrate skeletal vibration. When HA was crosslinked with PEGDE in the presence of IT, notable changes in the spectra of the NC and crosslinked HA were observed. In particular, the disappearance of the band at 598 cm^−1^ and the shift in the symmetric C-O band of the carboxylic acid from 1573 cm^−1^ to 1552 cm^−1^ are noteworthy. These changes reflect a substantial alteration in HA's structure after crosslinking with IT-PEGDE, which could significantly affect the scaffold's properties [46].

Figure S7e shows the FTIR spectra for pure b-FGF. The broad absorption band near 3200 cm^−1^ can be attributed to the O–H stretching of hydroxyl groups, typically found in the protein backbone, and possibly N–H stretching vibrations from amide groups (peptide linkages). The minor peaks in the 2944 cm^−1^ region correspond to C-H stretching vibrations, likely from the aliphatic chains present in the protein's amino acid side chains. The region between 1634 cm^−1^ and 1539 cm^−1^ typically represents the amide I and II bands, crucial for protein secondary structure identification. The amide I band around 1634 cm^−1^ is primarily due to C = O stretching vibrations of the peptide bond while the amide II band around 1539 cm^−1^ is attributed to N–H bending and C-N stretching vibrations. The region between 1388 and 1037 cm^−1^ showed several peaks that are due to C-N stretching and C-O stretching vibrations.

Figure S7j confirms the presence of b-FGF by the appearance of a peak at 1610 cm^−1^ which represents the α helix or secondary structure and the amide II functional group was observed at 1544 cm^−1^ from the NH bending and CN stretching vibration representing the ordered β-sheets. In addition to this, marked changes between the different scaffolds were observed in the 3265 cm^−1^ regions with the intensity of the OH group being different between the various scaffold formulations. However, the sensitivity of ATR-FTIR (used in this study) is around 3,500 ng/g according to Chan and Kazarian [47] which is far higher than the 75 ng/g for b-FGF present in the scaffolds which is a limitation of the current study. Therefore, the FTIR results described above cannot be conclusive and will require the more sensitive imaging approaches as suggested by [47]. The intensity of the OH peak was related to the degree of crosslinking as explained above, however, loading b-FGF did not show similar effects observed for the BSA loaded scaffolds. Scaffolds with higher content of HA showed higher amounts of OH groups hence more intense peaks such as in FCOL:HA 3:5-IT:PEGDE b-FGF scaffold. This could also indicate the disruption of the crosslink between HA and PEGDE upon loading b-FGF and the stronger affinity of b-FGF for the ECM components (FCOL and HA). Furthermore, the peaks at 2916 cm^−1^ and 2850 cm^−1^ assigned respectively to -CH_2_ and CH_3_ stretches of the long-chain aliphatic carbon were sharper for FCOL:HA 3:5-IT:PEGDE b-FGF scaffold compared to the other composite scaffolds.

### Nuclear magnetic resonance spectroscopy (NMR)

NMR was used to characterize the IPC crosslinked scaffolds and Fig. S8 shows representative ^13^C NMR spectra for the NC and IPC HA formulations. The spectra offer insight into the chemical environments of the samples, particularly those of HA following crosslinking with PEGDE and the effect of the other two polymers (SA and COL). The NC scaffolds showed unique signatures of HA, such as the pyranose ring carbons at 103.445 ppm, the glycosidic linkages at 85.196 ppm, the secondary alcohol carbons at 62.561 ppm, and the methyl groups at 24.174 ppm. Besides these distinct HA peaks, the spectra did not show any additional peaks that would indicate significant chemical interaction between HA and SA or COL. The glycosidic bonds, present in the spectra for both the NC scaffolds, undergo a slight shift to a more deshielded environment in the spectrum of the IPC scaffolds. For instance, the shift from 103.445 ppm to 105.554 ppm for the pyranose ring carbons and from 85.196 ppm to 88.947 ppm for the carbon involved in glycosidic linkage, suggest a change in the chemical environment, due to the crosslinking by PEGDE and indicates the successful integration of the PEG backbone into the polymer network. Further, the peak at 24.174 ppm, representing the methyl group or carbons adjacent to the amine groups of HA, shifted to a higher position at 28.206 ppm and indicates a change in the chemical environment of the methyl group, possibly due to the formation of new chemical bonds during the crosslinking process. In the spectrum for the IPC samples, additional new peaks (which are not present in the NC sample) and shifts observed at 105.554 ppm attributed to acetals or hemiacetals, suggest that PEGDE has reacted with hydroxyl groups in HA forming acetal or hemiacetal linkages. Studies conducted by Wende and colleagues [48] also utilized NMR to characterize the crosslinking of HA. They provided valuable insights into the characterization of crosslinked HA hydrogels, particularly those utilizing PEGDE and 1,4-butanediol diglycidyl ether (BDDE) as crosslinking agents. Their findings were deemed highly relevant to the current work, as they demonstrated the effectiveness of NMR spectroscopy in analyzing the chemical structure and crosslinking parameters of HA-based hydrogels. Overall, the NMR results complement the observations from GPC analyses and confirm the successful crosslinking of HA by the PEGDE in the presence of IT.

### Exudate handling properties

#### Swelling

Swelling of scaffold dressings in aqueous media is a desirable characteristic in biomedical applications including facilitating correct cell attachment, proliferation, and tissue ingrowth and affects nutrient and metabolite exchange. The swelling profiles of the CC scaffolds dressings (Fig. S9 (i)) revealed that when the concentration of HA increased such as in (SA-G:FCOL:HA 1:2:5), (SA-G:FCOL:HA 1:1:2) and (SA-G:FCOL:HA 2:3:3) the water uptake increased. This is because the enhanced osmotic pressure accelerated the penetration of the SWF into the matrix resulting in a higher degree of polymer swelling and formation of micro cavities. This is because higher concentrations of HA produced more pores within the scaffold matrix resulting in rapid hydration and subsequent swelling. Comparatively scaffold dressings with higher concentrations of SA-G and FCOL such as in (SA-G:FCOL:HA 3:4:1) and (SA-G:FCOL:HA 1:2:1) showed slower swelling kinetics. However, these scaffold dressings had a firm mechanical structure even after 24 h hydration in SWF as shown in the digital images (Fig. 1).

Excessive hydration and swelling of polymers lead to weaker mechanical strength of the swollen scaffold dressings; therefore, the water uptake studies alone were insufficient for selecting scaffold dressings with optimum characteristics. As a result, the gel strength of the hydrated scaffold dressings was measured using the texture analyzer. The results indicated that higher concentrations of HA favored swelling and erosion while lower concentrations of HA resulted in stronger gels. Figure S9i also shows the total work required for probe penetration calculated using the applied force and displacement values obtained at different time intervals after hydration of the scaffold dressings in SWF. The force required for the probe to penetrate the swollen gel matrix decreased with time as swelling increased, however the gel strength was reduced. The SA-G:FCOL:HA 1:2:5, SA-G:FCOL:HA 1:1:2 and SA-G:FCOL:HA 2:3:4 scaffold dressings and Promogran ™ exhibited lower values for work of penetration due to the hydrophilic nature of HA promoting higher water retention and subsequently weakening of the gel structure as seen in the digital images (Fig. S10a). In contrast, for the SA-G:FCOL:HA 3:4:1 and SA-G:FCOL:HA 1:2:1 scaffold dressings, the values for work of penetration were greater and showed slower swelling and higher gel strength which could be attributed to the crosslinking efficacy of Ca^2+.^ According to Palvi co-workers, the polyelectrolyte conformations are controlled by the fraction of ionized groups, and addition of counter ions such as Ca^2+^ results in less electrostatic interactions with media thus promotes conformational changes of the polymer [49].

The crosslinking resulted in improved structural integrity of the scaffold dressings containing higher quantities of SA-G. The results also revealed that incorporation of BSA disrupted the crosslinking causing the scaffold dressings to lose their structural integrity when swollen. This is due to the higher affinity of BSA for Ca^2+^ ions than SA-G via the Ca^2+^ ions binding to the carboxylic groups of BSA [50]. Conclusively the FCOL based scaffold dressings crosslinked with CaCl_2_ exhibited better swelling and water retention than the commercially available Promogran™.

The swelling profiles of the IPC scaffolds are presented in Fig. S9ii. Addition of IT-PEGDE resulted in increased water uptake compared to the NC reported in our previous study [19] and the CC scaffolds described above. This unexpected behavior can be attributed to the unique properties of the PEGDE crosslinker and its interaction with HA. PEGDE, being a hydrophilic polymer with a mixture of oligomers, created a more open and flexible network structure within the HA matrix. Despite the increased water uptake, these scaffolds maintained their structural integrity even after 8 h hydration (Fig. S9ii inset), therefore the study was extended to 72 h. The swelling of the scaffolds increased linearly until 1 h and then stayed constant thereafter up to 8 h. From the swelling profiles, it was evident that the point of maximum swelling differed among the different scaffolds tested. The % swelling index was high for formulations with higher HA concentration such as (SA-G:FCOL:HA 1:2:5-IT:PEGDE), (FCOL:HA 1:3-IT:PEGDE) and (FCOL:HA 3:5-IT:PEGDE) which confirms that the pore morphology affects the swelling behavior.

The faster water uptake can be explained by the higher porosity of these scaffolds compared to (SA-G:FCOL:HA 1:1:2-IT:PEGDE) and (SA-G:FCOL:HA 2:3:3-IT:PEGDE) and (SA-G:FCOL:HA 1:2:1-IT:PEGDE). The (SA-G:FCOL:HA 2:3:3-IT:PEGDE) and (SA-G:FCOL:HA 1:2:1-IT:PEGDE) scaffolds showed low water uptake within the first 8 h and although the water uptake drastically increased between 48–72 h, these scaffolds were mechanically less stable and started eroding on the edges as depicted by the gel strength measurements (Table S1) and the digital images shown in Fig. S10b. The lower water retention could be attributed to the lower concentration of HA hence the lower degree of crosslinking of these scaffolds. The (SA-G:FCOL:HA 1:2:5-IT:PEGDE), (FCOL:HA 3:1-IT:PEGDE) and (FCOL:HA 3:5-IT:PEGDE) formulations swelled rapidly and exhibited the highest % swelling values. Relating to the SEM results above, these scaffolds showed highly porous structures and further confirmed by the porosity data below confirmed their high porosity.

**Fig. 4:**
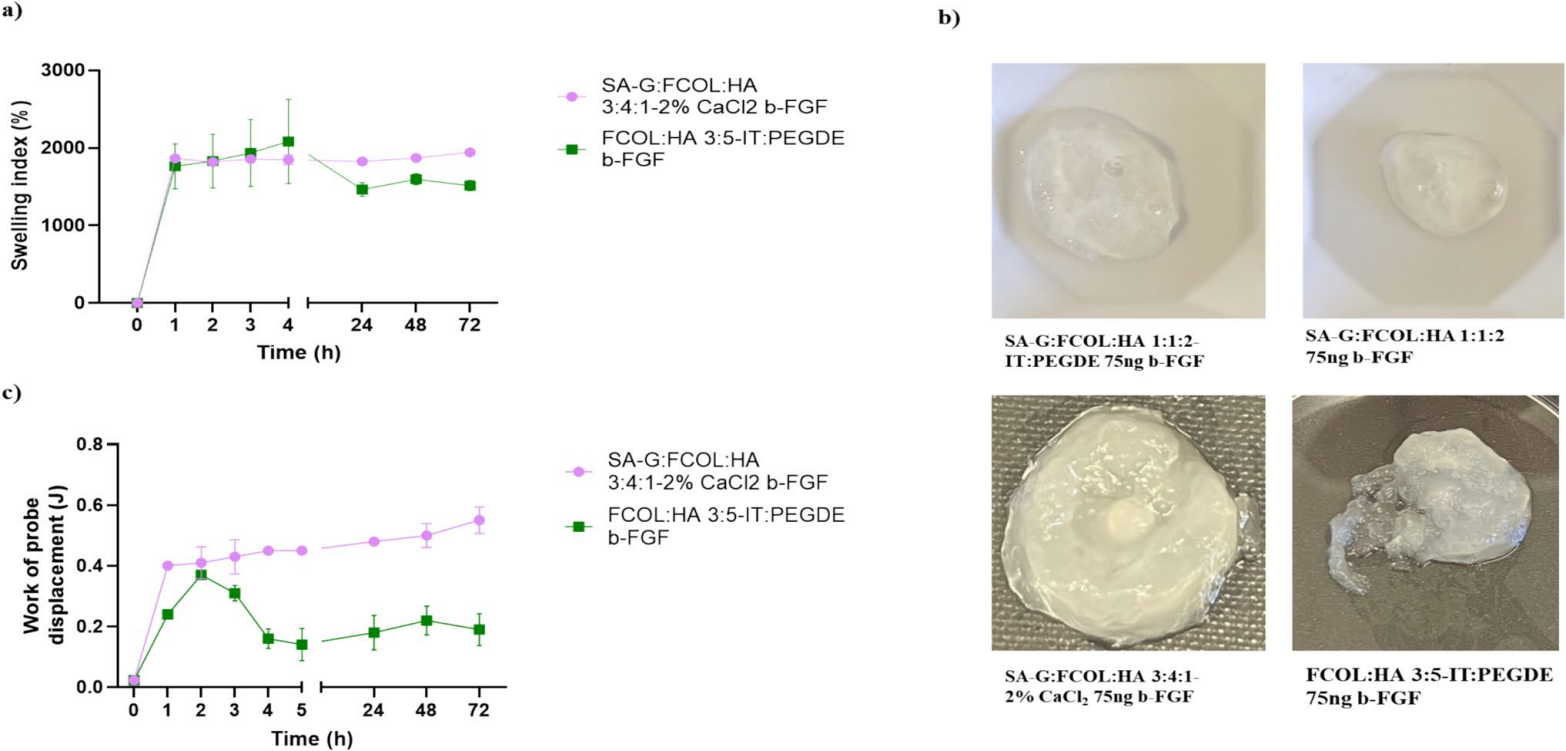
(**a**) Swelling profiles for growth factor loaded scaffolds; (**b**); Images of scaffolds after 1 h (top two images) and 72 h (bottom two images) showing disintegration of the former within 1 h and the maintaining of swollen structure for the bottom two formulations even after 72 h; (**c**) shows the total work of probe displacement by the swollen scaffolds during the swelling experiment at different time intervals

The swelling profiles and probe displacement into the swollen gels for the b-FGF loaded scaffolds are shown in Fig. 4a and c respectively. Only two of the four selected optimized scaffolds were able to swell and maintain their structural integrity over 72 h. These were the FCOL:HA 3:5-IT:PEGDE and SA-G:FCOL:HA 3:4:1 2% CaCl_2_ scaffolds with the former showing the highest swelling index at 5 h, but decreasing significantly afterwards, whilst the latter maintained a consistent swelling index up to 72 h. The SA-G:FCOL:HA 1:1:2 formulations disintegrated after 1 h as evidenced by the digital images shown in Fig. 4b (top images). These results suggest that the CaCl_2_ crosslinking produced scaffolds with better hydrogel properties able to hold onto fluid over a longer period. Further, the disintegration of the NC scaffolds suggests that crosslinking of some form is required for the growth factor loaded formulations to maintain their structure in the presence of exudate.

#### Porosity

For optimum wound healing and regeneration porous structures help provide homogenously distributed cell seeding density and effective oxygen and nutrient supply. The size, orientation and interconnectivity of the scaffolds have considerable impact on the regeneration process [51, 52]. Pore interconnectivity increases the overall surface area for cell–matrix interactions and cell attachment and facilitates cell in-growth into the scaffold dressings and has a positive impact on the deposition and exchange of the ECM elements. The pore alignment and size in this study were controlled by means of adjusting the freezing kinetics and incorporating an annealing step into the freezing phase of the freeze-drying cycle [42]. Studies have shown that scaffold dressings with a porosity of 60% to 90% are suitable for wound healing applications, thus providing sufficient surface area for nutrient exchange, cell activity and production of new ECM [53]. Therefore, the scaffold dressings developed had acceptable porosity ranging from 64 ± 7 to 81 ± 7. Maintaining the balance between mechanical stability and porosity is important.

The crosslinking of scaffold dressings with Ca^2+^ had a positive impact on the mechanical stability of scaffold dressings and did not reduce the porosity. Although the mean porosity of the blank and BSA-loaded formulations was marginally different (Table S2a), the difference in the porosity upon loading BSA was not statistically significant (*p* > 0.05). The IPC scaffolds exhibited acceptable porosity within the range of 60 to 90% (Table S2b). In contrast to the CC scaffolds, the IPC scaffolds showed higher porosity which will provide sufficient surface area for nutrient exchange and cell adhesion. According to Xu and Liu, formation of new blood vessels takes several days after application of 3D scaffolds. Hence, designing a robust, reproducible and biodegradable scaffold that closely mimics human tissue and can function and be retained at the wound site under load bearing conditions is challenging [54]. Thus, the IPC scaffolds developed in this study with their highly porous architecture can retain copious quantities of water and maintain their mechanical structure making them potential carriers for delivery of macromolecules and cells.

The growth factor loaded scaffolds exhibited acceptable porosity ranging from 70 to 90% (Table 2) and these were higher than the corresponding BSA loaded formulations though not all showed significant changes. However, the SA-G:FCOL:HA 1:1:2 b-FGF scaffold showed significantly lower porosity (70%) than the corresponding BSA loaded formulation which had a porosity value of 91%. Interestingly, the two formulations that disintegrated within an hour during the swelling studies showed the lowest porosity and exhibited thinner walls and therefore generally weaker.

**Table 2:** Shows the exudate handling properties of the optimized b-FGF loaded scaffolds (*n* = 2 ± SD)

Sample name	Porosity (%) ± SD	WVTR (g/m^2^day^−1^) ± SD	AW (%) ± SD	EWC (%) ± SD
SA-G:FCOL:HA 3:4:1–2% CaCl_2_ b-FGF	90 ± 1	2898 ± 698	416 ± 57	95 ± 1
SA-G:FCOL:HA 1:1:2 b-FGF	70 ± 10	4254 ± 50	921 ± 44	92 ± 2
SA-G:FCOL:HA 1:1:2-IT:PEDGE b-FGF	82 ± 3	4181 ± 206	1190 ± 16	95 ± 1
FCOL:HA 3:5-IT:PEDGE b-FGF	87 ± 1	1685 ± 26	1378 ± 99	93 ± 0

#### Water absorption (AW) and equilibrium water content (EWC)

A wound dressing must not only absorb wound exudate, but also retain the excess fluid together with its protease solutes, while concurrently preventing desiccation. This is especially important in the case of chronic wounds where the skin barrier properties become compromised and where there is increased leakage across the injured skin. Therefore, hydration properties represent an important aspect in the design and development of wound dressings and scaffold dressings.

The AW and EWC of the CC scaffold dressings (Table S2a) ranged from 412 ± 157 to 1046 ± 250% and 79 ± 6 to 91 ± 2% respectively. The results showed higher AW and EWC for scaffold dressings containing higher quantities of HA such as (SA-G:FCOL:HA 1:1:2), (SA-G:FCOL:HA 2:3:3), and (SA-G:FCOL:HA 1:2:5) compared to NC scaffold dressings [19] that showed higher AW for scaffold dressings with higher concentrations of FCOL. On the other hand, SA-G:FCOL:HA 1:1:2 and SA-G:FCOL:HA 3:4:1, scaffold dressings showed better water absorption capacity. The reduced water absorption of FCOL could be due to the electrostatic interaction between the Ca^2+^ and the negatively charged ions in FCOL as reported by Rhee et al. [55]. In addition to the ionic strength many other factors such as degree of crosslinking and the pore size and interconnectivity can influence the water sorption–desorption kinetics [55]. The water content in human epidermis is around ~ 70%, therefore, to create the osmotic balance and maintain a moist wound environment for optimum cell function, a wound dressing or scaffold dressings should have an EWC value closer to that of the human epidermis. Furthermore, studies conducted by Shahabuddin and co highlighted that hydrogels with EWC of 85–90% were able to stimulate the proliferation and migration of fibroblasts and keratinocytes compared to hydrogels with higher EWC (90–95%) which had no influence on the biological activity of the cells [56].

HA is a biocompatible, biodegradable and hydrophilic polymer that can absorb large quantities of water up to 1000 times its solid volume, due to its high molecular weight. The high water retention capacity is attributed to the abundance of carboxylic and hydroxyl groups on its backbone. However, due to its highly soluble nature, HA is mechanically unstable and shows rapid disintegration. The results also showed that HA was unable to absorb and retain water as reported by the literature. To overcome these drawbacks; enhance the mechanical stability and improve the water absorption capacity of HA, PEGDE was used to crosslink HA within the composite formulations in the presence of IT. The AW and EWC of the IPC scaffolds are shown in Table S2b. The AW ranged from 416 ± to 1378 ± 99 while the EWC ranged from 79 ± 2 to 93 ± 0% and these values were marginally higher than the CC formulations. However, as evidenced by the digital images in Fig. S10a & b, these scaffolds were more stable after absorbing similar volumes of SWF but the FCOL:HA 3:5-IT:PEGDE and FCOL:HA 3:1-IT:PEGDE scaffolds exhibited the lowest AW. The AW and EWC of the scaffolds increased significantly upon loading the BSA which can be attributed to the electrostatic interaction between BSA as previously discussed above [57].

The AW and EWC properties of the b-FGF loaded scaffolds are presented in Table 2. The results showed high AW for the IPC scaffolds followed by the corresponding NC and CC scaffolds respectively. The high AW of the IPC scaffolds (SA-G:FCOL:HA 1:1:2 b-FGF and FCOL:HA 3:5 b-FGF) was attributed to the abundance of COOH and OH groups of HA. The crosslinking further improved the retention of large volumes of exudate within the polymeric matrix. In contrast to the corresponding BSA loaded scaffolds, the b-FGF loaded scaffolds exhibited significantly (*p* < 0.05) lower AW values which could be attributed to the lower concentrations of growth factor loaded compared to BSA.

#### Water vapor transmission rate (WVTR)

To achieve a satisfactory environment for wound healing, a dressing must have a suitable WVTR and coupled with the AW controls the fluid balance [58]. Several factors control the WVTR of scaffold dressings, with material properties and porosity playing important roles. The WVTR for normal skin is 204 g/m^2^, however, injury disrupts this normal value, with the WVTR of injured skin ranging from 279 g/m^2^day^−1^ for a first degree wound to 5138 g/m^2^day^−1^ for a granulating wound. Furthermore, studies have highlighted that a WVTR of 2500 g/m^2^day^−1^ per day would provide adequate level of moisture content without risking wound dehydration for 24 h [59].

Álvarez-Suárez and colleagues reported that the amount of exudate produced by a burn wound is ~ 5000 g/m^2^day^−1^ while exudate production for chronic ulcers ranges from 4000 to 12,000 g/m^2^day^−1^. Subsequently it is reasonable to say that the CC scaffold dressings with WVTR ranging from 2478 ± 57 to 4009 ± 2708 g/m^2^day^−1^ can maintain a favorable moist healing environment for burns and mild exuding chronic wounds. It also implies that these scaffold dressings can prevent maceration due to exudate collection. In contrast, the commercial COL based Promogran™ typically prescribed for chronic wounds exhibited a WVTR of 49,459 ± 207 g/m^2^day^−1^ that might cause excessive dehydration of the wounds, especially low to moderate exuding wounds [60].

The WVTR of the IPC scaffolds presented in Table S2b was in the mid-range (2368 ± 59 to 2195 ± 28 g/m^2^day^−1^) of acceptable moisture loss from the injured skin. Although there was a reduction in the pore volume in comparison to the CC scaffolds, the WVTR values of the IPC scaffolds were deemed suitable for mild exuding wounds [61]. Despite the high AW, these scaffolds were unable to retain the exudate as was evident by the higher WVTR values 4254 ± 50 g/m^2^day^−1^ for SA-G:FCOL:HA 1:1:2 scaffold and 4181 ± 206 g/m^2^day^−1^ for the corresponding IPC scaffolds. Alginate based scaffolds are known as highly absorbent and considered suitable for highly exuding wounds [61]. The composite SA-G:FCOL:HA 3:4:1 CC scaffold dressing had significantly lower AW compared to those containing higher concentrations of HA, however, these scaffolds were still able to retain the absorbed moisture and maintain their structural integrity, with a WVTR of 2898 ± 698 g/m^2^day^−1^.

Based on the WVTR criteria outlined above, the results achieved in this study suggest that the porous CC SA-G:FCOL:HA 3:4:1 scaffolds loaded with b-FGF, had the optimum water uptake and retention ability and therefore potential to maintain a moist wound environment without dehydrating the wound (Table 2). On the other hand, the FCOL:HA 3:5-IT:PEGDE b-FGF with a WVTR value of 1685 ± 25.27 g/m^2^day^−1^ which was lower than the expected ideal values for WVTR, was not deemed ideal as this may result in exudate accumulation. This implies that this scaffold will not be suitable for applying to highly exuding wounds and may only work under mild to low exudate conditions. On the other hand, the NC and IPC SA-G:FCOL:HA 1:1:2 scaffolds with WVTR values of 4254 ± 50 g/m^2^day^−1^ and 4181 ± 206 g/m^2^day^−1^ respectively, were relatively very high. According to Revi and co, such high WVTR values might cause the wound bed to become desiccated and subsequently lead to loss of integrity and increases the possibility of tissue necrosis [62].

#### Evaporative water loss (EWL)

Since wound covering is an important first line protection for maintaining the balance in body fluids, the quantification of EWL through a wound dressing is an effective method for estimating body’s water loss. Xu and co-workers reported that EWL from wounds is twenty times greater than that of normal skin [63]. If a wound is exposed to air, it dehydrates, and cells present in such a dry or low moisture microenvironment will lose vitality and die.

The EWL through the CC scaffold dressings is shown in Fig. S11a and showed about 20% water loss within the first hour which was similar to the commercial dressing Promogran™ tested under similar conditions.

**Fig. 5:**
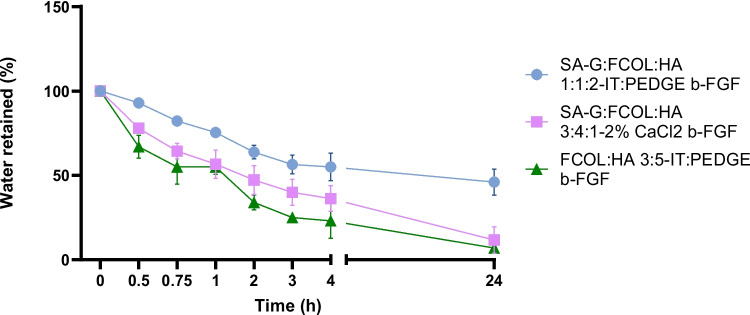
EWL profiles b-FGF loaded crosslinked scaffolds

From the EWL profiles of the IPC formulations (Fig. S11b), it was evident that the scaffold dressings retained about 70% water that gradually decreased to 50% water loss after 5 h hydration. In contrast to the NC scaffold dressings the water loss from these scaffold dressings was slower (owing to their hydrogel-like properties). On the other hand, the CC scaffold dressings showed better water retention than the IPC scaffold dressings. After 24 h incubation the scaffold dressings with higher degree of crosslinking such as FCOL:HA 3:5 retained higher amounts of exudate followed by SA-G FCOL:HA 1:1:2 with a water retention of ~ 30 and 24% respectively. However, the BSA loaded scaffold dressings were unable to retain any fluid within the scaffold dressings matrix due to the interruption to the crosslink by the loaded protein as discussed earlier. However, the main target protein for this research is growth factors and the EWL profiles of b-FGF loaded scaffolds is presented in Fig. 5.

No readings were possible for SA-G:FCOL:HA 1:1:2-IT:PEDGE b-FGF scaffolds as they disintegrated very rapidly. The EWL profile of the scaffolds showed 45%, 64% and 22% exudate loss for SA-G:FCOL:HA 1:1:2 b-FGF, 3:4:1–2% CaCl_2_ b-FGF and FCOL:HA 3:5-IT:PEGDE b-FGF respectively during the first four hours of the study. After 24 h incubation, the scaffolds retained 7% to 45% of water, with the NC scaffolds retaining the highest amounts of moisture and the FCOL:HA 3:5-IT:PEGDE b-FGF retaining the lowest amount of water at 7%. Compared to the corresponding BSA loaded scaffolds the EWL values for b-FGF loaded scaffolds increased and therefore exhibited better water retention ability after 24 h. Interestingly, the SA-G:FCOL:HA 3:4:1–2% CaCl_2_ b-FGF scaffold which showed the optimum WVTR values also had good EWL performance. According to Revi and co, epithelial cells need a moist environment to migrate and re-epithelialized faster, hence the SA-G:FCOL:HA 3:4:1–2% CaCl_2_ b-FGF scaffold with optimum hydration, flexural rigidity, indicates its potential suitability as a scaffold for faster and better healing of highly exuding wounds [62].

### In vitro drug dissolution and kinetic release mechanism

The ability to release loaded drug from medicated scaffold dressings in a controlled way is important for achieving optimum therapeutic activity and ultimate wound healing. The release of BSA from the CC scaffold dressings showed a biphasic profile in SWF pH 7.4 (Fig. S12a) with the BSA released rapidly in the first 60 min followed by a slower rate of release up to 72 h. The initial phase of drug release, also known as burst release, indicates the release of the free form of the protein that was not entangled within the polymeric matrix. All the scaffold dressings released at different rates for the initial 60 min of the study. Scaffold dressings with highest concentration of HA (SA-G:FCOL:HA1:2:5–2% CaCl_2_) exhibited a lower burst release followed by SA-G:FCOL:HA 3:4:1–2% CaCl_2_ and SA-G:FCOL:HA1:1:2–2% CaCl_2_. In contrast, SA-G:FCOL:HA 1:1:2–2% CaCl_2_ scaffold dressings showed the highest burst release of BSA from the formulation matrix. The fast initial release of the protein could be helpful in initiating hemostasis by activating the relevant growth factors and cytokines in the coagulation cascade, while the slow and gradual release in the subsequent hours can ensure that proliferation and angiogenesis can be stimulated and sustained. Such biphasic drug release profiles are ideal for targeting local inflammation and pain associated with chronic wounds as the first 12—48 h are critical in a wound healing process.

The release profiles of the BSA loaded IPC scaffold dressings (Fig. S12b), show that four of the six formulations tested showed an initial almost linear release in the first 8 h followed by a more sustained release up to 72 h. Scaffold dressings with lower HA content released BSA at a faster rate and higher percentage cumulative release compared to those with higher HA content. This is confirmed by the two formulations (FCOL:HA 1:3-IT:PEGDE and SA-G:FCOL:HA-IT:PEGDE) which did not release the loaded BSA over the 72-h testing period. This suggests that the BSA might be interacting with the HA for these crosslinked scaffold dressings as discussed in the sections above. This is further supported by the fact that the release profiles do not follow the same pattern as the swelling profiles for the respective formulations, which is typically expected for swellable matrix systems and confirms the complex nature [64, 65] of the composite formulations together with the crosslinking by IT-PEGDE and will need further investigations to elucidate the molecular mechanisms involved. Due to costs, it was not possible to perform in vitro release studies for the growth factor loaded scaffolds, however, the release of the larger BSA from the scaffolds has established the proof of principle for potential use of the scaffolds to deliver biologically active protein-based drugs for wound healing applications.

The release mechanism is a way to assess the kinetics of the drug dissolution and its behavior in the biological system to supply and maintain the desired drug concentration [66]. Therefore, to evaluate the mechanism of the BSA release from the scaffold dressings, the release profiles were fitted to various kinetic models and the results are summarized in Table S3 of supplementary information. For the CC scaffolds, the values recorded for the correlation coefficient (R^2^) were higher for the Higuchi and the first-order models which indicates that the release of BSA involved a series of events when the scaffold dressings came into contact with the dissolution medium. The sequence involved water absorption leading to the relaxation of the polymer chains allowing them to swell and form a thick gel around the scaffold dressings. Therefore, the BSA was released from the CC scaffolds via diffusion through the swollen gel layer and subsequently via erosion of the swollen polymer layer. The only exception was SA-G:FCOL:HA 1:1:2–2% CaCl_2_ BSA where the R^2^ value from fitting to the first order fitting was very low (< 0.1).

In the case of the IPC scaffolds, the best fit was the Korsmeyer-Peppas equation for all the formulations with R^2^ values ranging from 0.83 – 0.97 implying drug release involving swelling and subsequent erosion of the matrix. The n values ranged from 0.48 – 0.86 which is indicative of drug release via anomalous (non-Fickian) diffusion.

### Stability evaluation and HPLC determination of BSA content

Figure S13 shows the BSA contents of optimized scaffolds estimated using HPLC. The exposure of protein to the varying conditions had a marked effect on the chemical stability of the model protein.

The scaffolds stored in refrigerator showed very minimal protein degradation or fragmentation throughout the evaluation period, except for the NC SA-G:FCOL:HA 2:3:3 scaffold. The assayed BSA content ranged between 93—100% as specified by the British Pharmacopeia [67]. Storage at 25 °C and relative humidity of 60% showed significant reduction in the protein content ranging from 87–52% after twelve months of storage. The stability studies did show the impact of crosslinking compared to the NC scaffold (SA-G:FCOL:HA 2:3:3). A reduction in the BSA content in the NC scaffolds shows that these scaffolds were not efficient for protein loading. Furthermore, when comparing the protein content for the IPC and CC scaffolds, the presence of salt had an impact on the denaturation of the protein. Each protein has its own unique structure and, if the temperature or the pH of its environment changes the disulfide interactions within the protein may be disrupted causing protein to lose its three-dimensional structure and get denatured. The CC scaffolds showed marked reduction in protein content with SA-G:FCOL:HA 1:2:5–2% CaCl_2_ scaffold showing 50% reduction in protein content whilst SA-G:FCOL:HA 1:1:2–2% CaCl_2_ showed 30% reduction in protein content when stored under ICH conditions. The higher amounts of unreacted CaCl_2_ in the CC SA-G:FCOL:HA 1:2:5 scaffold compared to CC SA-G:FCOL:HA 1:1:2 scaffold accounted for the differences observed in the protein denaturation.

### MTT assay

The cell viability and proliferation of the HEK and HDF cells in contact with CC scaffold dressings after 24 h and 48 h were studied using MTT assay and the findings are presented in Fig. S14a and b. The results showed cell viability above 80% for all treatments which further increased marginally when BSA was loaded into the scaffold dressings. The difference in the viability of the different treatments was not significant. According to Xue and coworkers, fibroblasts are 100 times less sensitive to calcium compared to keratinocytes while in another study Ko et al., found that cell–cell adhesion in fibroblasts does require calcium [68, 69]. The results in this study did not show significant difference (*p* > 0.05) between treatment of HDF and HEK primary cells. The cell viability of both HDF and HEK cells were significantly different when treated with CC SA-G:FCOL:HA scaffold dressings compared to the NC and the IPC scaffold dressings. Calcium deficiency in chronic wounds have been reported by several studies, which suggests that the residual of CaCl_2_ might further enhance chronic wound healing [26]. However, further tests need to be performed to assess the concentration that might inhibit the proliferation of HEK.

PEGDE is a water-soluble additive that is used to crosslink polymers bearing amine, hydroxyl or carboxyl groups. Due to its low toxicity compared to other crosslinkers such as glutaraldehyde, it has been used in various pharmaceutical applications. Additionally, IT is a bio-based dicarboxylic acid that is often used as a comonomer to facilitate the internal crosslinking. During crosslinking some of the crosslinkers may be unreacted and this could exhibit toxicity at high concentrations and therefore their removal from the formulation is necessary. As previously reported, the epoxide groups can bind to proteins and nucleic acid that can be carcinogenic or mutagenic [70–74]. The MTT results for IPC scaffolds are shown in Figs. S15a and b showed a decreased % cell viability below 70% in the case of HDF for most of the scaffold dressings. The scaffold dressings with higher HA concentration exhibited higher % cell viability compared to those with lower concentrations of HA. This could be due to the higher degree of crosslinking that consumed most of the crosslinker, therefore very little or no unbound PEGDE, resulting in better cell viability. This does confirm the efficiency of the gel washing which removed the unbound crosslinker from the gel. In a study conducted by [75] it was highlighted that the unbound crosslinker at concentrations of 500, 1000 and 5000 µg was safe in mice. Previous studies of the unbound crosslinker confirmed the efficiency of hydrogel washing for removal of the unbound crosslinker. However, further cytotoxicity studies need to be performed to assess the skin toxicity of PEGDE [76]. Furthermore, PEK cells showed better tolerability to the residual, unbound crosslinker when cells were treated with blank scaffold dressings compared to the HDF cells, but the % cell viability was below the acceptable range.

**Fig. 6:**
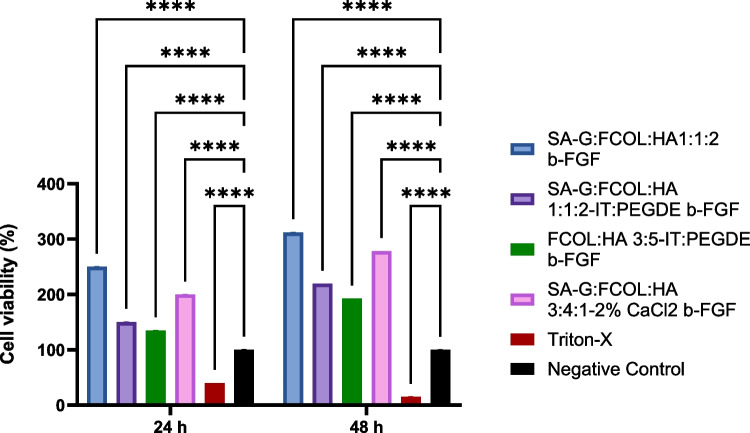
Shows the results for the MTT assay obtained by analyzing the viability of HDF treated with the optimized scaffolds loaded with 75 ng b-FGF. The data are shown as the mean of the cell viability ± standard deviation (*n* = 6). The statistical analysis showed a significant difference *p* < 0.05 between the control and the treated cells after 48 h.

Figure 6 shows the cytotoxicity profiles of the b-FGF loaded scaffolds. The results confirmed that the presence of b-FGF promoted the proliferation and survival of HDF cells, and all samples showed a significant increase (*p* < 0.05) in cell viability after 24 h and 48 h treatment compared to the untreated cells. However, this claim is limited by the relatively high concentrations of the growth factor and the limited cell lines used in this study and further work is therefore required.

### Whole blood clotting assay

Skin injuries with uncontrolled bleeding and bacterial infection are major challenges in clinical wound management. Designing a multifunctional mechanically stable, biodegradable scaffold dressing that can absorb large volumes of exudate and blood and provide hemostatic effect is of current research and clinical interest. Crosslinking is one of the modification methods employed to enhance mechanical stability of polymeric scaffolds with the material becoming stiffer and preserving the 3-dimensional structure following hydration in the presence of high amounts of exudate.

Calcium plays an important role in wound healing and during the hemostasis phase it aids in blood clotting by facilitating the formation of the platelet plug. In addition to the benefits of enhancing mechanical stability, it was hypothesized that incorporation of calcium aided in the formation of a stronger platelet plug. The results of blood clotting assay are summarized in Figs. S16 and S17.The scaffold dressings with higher concentration of SA-G showed lower BCI values such as in SA-G:FCOL:HA 3:4:1 and SA-G:FCOL:HA 2:3:3 scaffold dressings, indicating the higher crosslinking density of these formulations compared to those containing higher concentrations of FCOL and HA.

The in vitro study of the prepared IPC scaffold dressings dressing indicated a low blood clotting index, high platelet adhesion capacity and accelerated clotting time (Fig. S17a). From the results it was evident that the scaffold dressings containing higher quantities of HA exhibited a higher degree of crosslinking with IT-PEGDE which in turn delayed the movement of fluid and decreased the molecular mobility of the polymer chains. The differences between the BCI of control and the scaffold dressings was significant (*p* < 0.05). On the other hand, scaffold dressings with lower crosslinking degree were able to absorb higher quantities of blood in a shorter time, subsequently the blood clotting was initiated faster resulting in the formation of denser clots. The FCOL:HA 3:5-IT:PEGDE BSA and SA-G:FCOL:HA 1:2:5-IT:PEGDE BSA scaffold dressings, however, showed better hemostatic effects. This could be attributed to the faster hydration and absorption of blood due to surface morphology of the scaffold dressings that resulted in lower BCI.

**Fig. 7:**
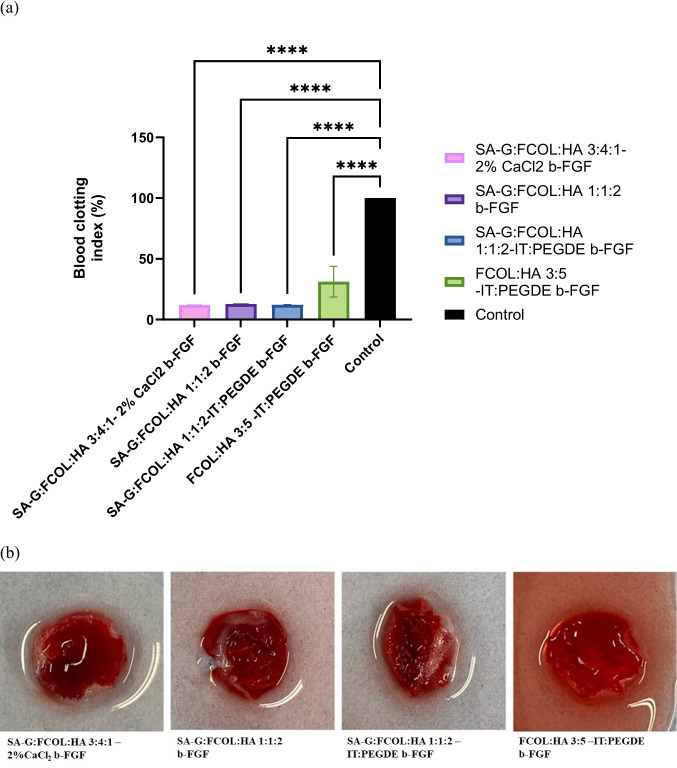
(**a**) The blood clotting index (BCI) of the b-FGF loaded scaffolds after being in contact with whole human blood, (**b**) the digital images of the scaffolds after 10 min incubation. The experiment was performed as (*n* = 3). One way ANOVA statistical analysis showed a significant difference between the control and the scaffolds *p* < 0.05

The BCI of the b-FGF loaded scaffolds is presented in Fig. 7a. The results indicate the highest BCI for the FCOL:HA 3:5-IT:PEGDE b-FGF and the lowest for the SA-G:FCOL:HA 3:4:1 2% CaCl_2_ b-FGF. This suggests that this scaffold was able to initiate a blood clotting cascade rapidly with a significant decrease of BCI after 10 min incubation compared to the control and other scaffolds and this was further evidenced by the digital images shown in Fig. 7b). The low BCI values of the SA-G:FCOL:HA 3:4:1 2% CaCl_2_ b-FGF scaffold are attributed to the high FCOL content which acts as a natural anticoagulant and plays an important role in hemostasis and thrombogenesis. Washing the scaffold dressing with deionized water did not disrupt the crosslinks between the fibrin clots compared to the FCOL:HA 3:5-IT:PEGDE b-FGF formulations that were unable to maintain the mechanical strength and resulted in lysis of the clot [77] and might therefore not be ideal as a rapid hemostatic wound dressing.

The results achieved in this study confirmed that in designing a hemostatic wound scaffold dressing many factors should be considered. Furthermore, CaCl_2_ was an efficient crosslinker that not only enhanced the mechanical stability of the scaffold dressings but provided good hemostatic effects, however, further investigation of the optimum concentration of CaCl_2_ at a molecular level will be required.

### Scratch assay

**Fig. 8:**
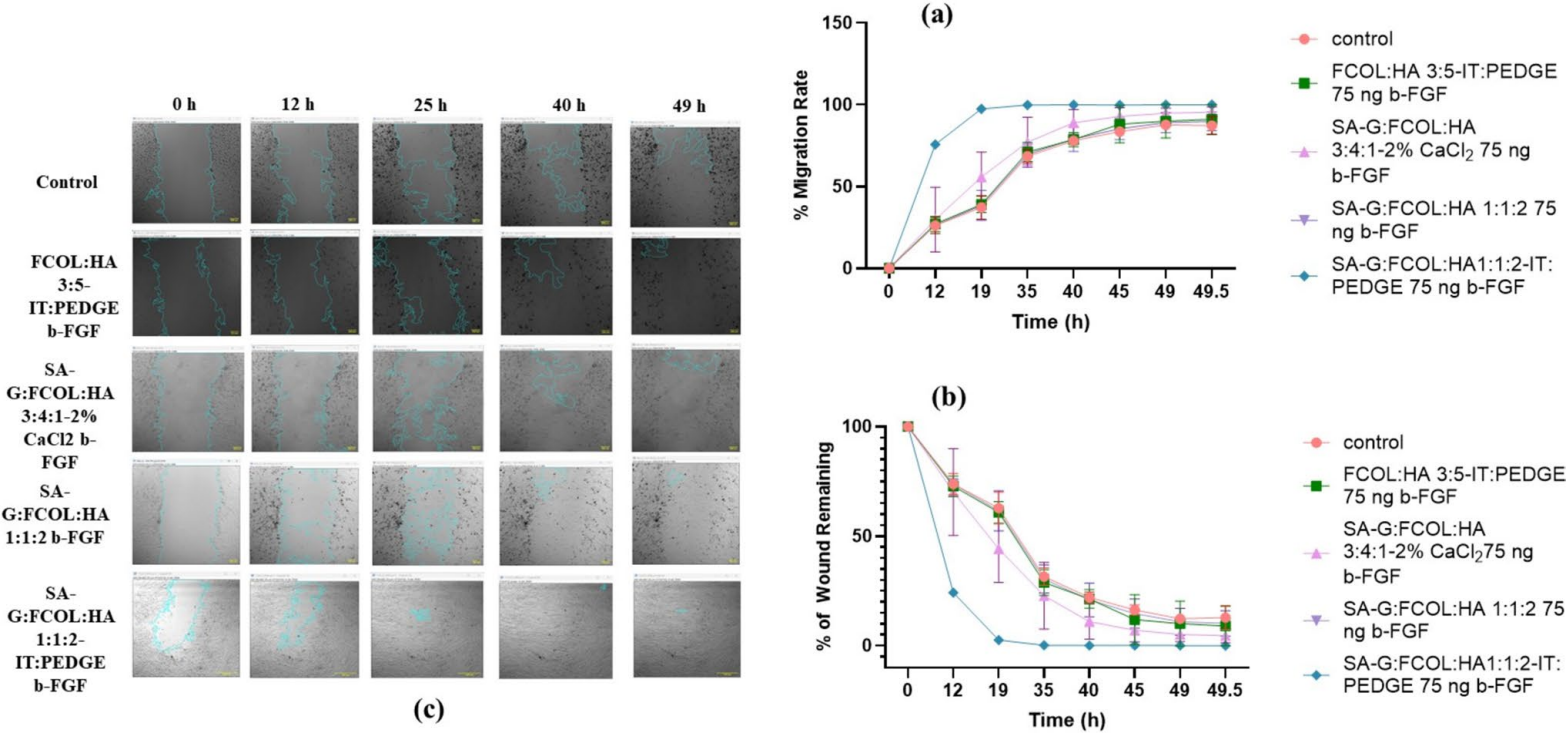
The figure depicts (**a**) the percentage of cell migration rate and (**b**) the percentage of wound remaining over time for different treatment groups in scratch assay. The data points represent the mean ± standard deviation of the mean for each treatment at various time points (*n* = 3); (**c**) representative microscopic images showing the migration of human dermal fibroblast cells treated with b-FGF loaded scaffolds over time. Each row represents a different treatment condition, and each column shows the progression of wound closure at specific time points. The highlighted area (blue boundary) shows the wound gap

The present study evaluated the efficacy of various FCOL-HA based formulations for promoting cell migration and wound healing using an in vitro scratch assay. This assay involves creating a controlled wound-like gap in a cell monolayer and observing the migration of cells to close the gap, and is a widely accepted method to study cell migration and wound healing in a controlled environment. The findings demonstrated that the SA-G:FCOL:HA:1:1:2-IT:PEDGE b-FGF formulation significantly enhanced both cell migration (Fig. 8a) and wound closure (Fig. 8b) rates compared to other treatments and the control. This formulation achieved near-complete migration by 19 h and sustained this high rate throughout the study. Such rapid and sustained migration suggests that the combination of SA-G:FCOL:HA with IT:PEDGE and b-FGF creates a highly conducive environment for cell movement. In contrast, while showing improved migration rates compared to the control, other treatments did not reach the same level of efficacy as the IPC SA-G:FCOL:HA:1:1:2 formulation. The control group, lacking active components, exhibited the slowest migration rates, highlighting the necessity of these bioactive ingredients for enhanced cellular activity and the superior performance of the IPC SA-G:FCOL:HA 1:1:2 formulation. The wound closure data further supports the superior performance of the IPC SA-G:FCOL:HA 1:1:2 b-FGF scaffold dressing which facilitated almost complete wound closure by 35 h, significantly (*p* < 0.05) faster than other formulations. The active components, including SA, FCOL, and HA, are known to promote cell migration and wound healing, thereby accelerating the overall wound-healing process. The rapid wound closure indicates that this formulation not only promotes cell migration but also accelerates the overall wound-healing process.

The statistically significant differences observed in the early time points (Fig. S18) highlight the rapid action of this formulation and making it a promising candidate for clinical applications aimed at improving wound healing. When integrated into SA hydrogels, natural polymers such as HA and FCOL can enhance cellular infiltration and tissue regeneration. These polymers also promote angiogenesis, re-epithelialization, and provide structural support for cell migration, creating a favorable environment for cell attachment, proliferation, and migration during wound healing. However, it is important to note that these polymers have a limitation, which is the fact that their relatively low mechanical strength can restrict their effectiveness [78]. Our study shows that by crosslinking scaffolds with agents such as IT and PEGDE the physical, mechanical and fluid handling properties of the hydrogel scaffolds can be significantly improved. This process ensures their structural integrity and controlled degradation rates, making them suitable for tissue regeneration.

Fibroblast growth factor (b-FGF) is an important cytokine that accelerates skin regeneration and is recognized as a potent mitogen and chemo-attractant for many cells in vivo and is a crucial growth factor for fibroblasts and capillary endothelial cells in vitro. b-FGF not only promotes skin regeneration by enhancing the migration and proliferation of fibroblasts but also plays a vital role in the migration and proliferation of endothelial cells, thereby promoting angiogenesis. This process is essential for granulation tissue formation, re-epithelialization, and remodeling, vital components of the healing process [79, 80]. The findings in this study demonstrated and confirmed the effectiveness of b-FGF and offer promising potential for therapeutic use in wound care by enhancing skin regeneration and improving patient outcomes.

## Conclusions

Both CaCl_2_ and PEDGE were successfully able to respectively crosslink SA and HA present in composite SA-G:FCOL:HA gels which produced elegant scaffold dressings after freeze-drying. Both optimized CC and IPC scaffold dressings demonstrated better functional properties compared to the corresponding NC scaffolds and the commercially available biological dressing (Promogran™). The lyophilized scaffolds exhibited interconnected pores that aided their hydration and swelling over 24 h and this was affected by the concentration of the polymers and the crosslinking density. They exhibited ideal water retention capacity while maintaining their structural and mechanical integrity when in contact with SWF compared to the NC scaffold dressings. Furthermore, the crosslinked scaffold dressings achieved WVTR that was in the acceptable range and can maintain a favorable moist healing environment for chronic wounds. However, the IPC scaffold dressings showed better swelling and WVTR than the CC scaffolds and improved the swelling index three times compared to the NC scaffolds. The crosslinked scaffold dressings further showed desirable characteristics such as hardness and adhesion confirming their ease of handling and remaining on the wound surface while preventing damage to newly formed tissue. The BSA release from the CC and IPC scaffold dressings showed a biphasic profile, with an initial burst release of the free protein followed by a slower sustained release up to 48 h which is expected to avoid the need for daily dressing changes. The MTT assay confirmed the safety and biocompatibility of the scaffold dressing with cell viability above 70%. However, the effect of residual PEGDE on cell viability was evident but this was easily resolved by gel washing prior to freeze-drying. In the case of the b-FGF loaded composite scaffolds no cytotoxic effect against HDF cells was evident, including IPC scaffolds and therefore it is possible that the growth factor may have overshadowed the cytotoxic effects of any residual PEGDE. In vitro coagulation assay confirmed the excellent hemostatic effects for all composites containing SA-G confirming the importance of calcium ions in the clotting cascade. Therefore, these biomaterial-based bioactive scaffold dressings may find application as superabsorbent medicated dressings for application in chronic wounds. The significance of this study lies in the potential of the CC and IPC scaffolds as multifunctional, multi-targeted and therapeutic dressings to overcome challenges with existing products to treat chronic wounds. Further, they could be applied as delivery platforms for other active ingredients (e.g. antimicrobials) relevant to chronic wound healing and tissue regeneration. However, the study is limited by the absence of in vitro drug release data for the b-FGF loaded scaffolds and future studies will need to confirm the release of the loaded growth factor. In addition, in vivo wound healing testing in mice will be required in the future to confirm their ability to enhance chronic wound healing.

## Supplementary Information

Below is the link to the electronic supplementary material.

Supplementary file1 (PDF 3.41 MB)

## Data Availability

The datasets generated during the current study (beyond supplementary information) are available from the corresponding author on reasonable request.
