# Supplementary material for: Medicated and multifunctional composite alginate-collagen-hyaluronate based scaffolds prepared using two different crosslinking approaches show potential for healing of chronic wounds
Source: Drug Deliv Transl Res. 2024 Dec 11;15(7):2483–508. doi: 10.1007/s13346-024-01745-0 (PMC12137399; doi:10.1007/s13346-024-01745-0)
Supplement: Supplementary file 1 — Supplementary file1 (PDF 3.41 MB) [file 13346_2024_1745_MOESM1_ESM.pdf]

SUPPLEMENTARY

Medicated and Multifunctional Composite Alginate-Collagen-Hyaluronate Based Scaffolds Prepared Using Two Different Crosslinking Approaches Show Potential for Healing of Chronic Wounds

Meena Afzali, Nessa Esfandiaribayat, Joshua S. Boateng*

School of Science, Faculty of Engineering and Science, University of Greenwich at Medway, Chatham Maritime, Kent, UK, ME4 4TB

**Correspondence: j.s.boateng@gre.ac.uk, joshboat40@gmail.com*

Supplementary notes **3**
1. Textural Properties of Scaffolds 3
1.1 Analysis of textural parameters, including firmness, consistency, cohesiveness, and viscosity index (see Table S1).......... 3
2. GPC Analysis of Crosslinked Scaffolds 4
2.1 Gel permeation chromatography (GPC) 4
2.2 Gel permeation chromatography (GPC) 4
Supplementary Figures **6**
Figure S1. 6
Figure S2. 6
Figure S3i.………………………………………………………………………… 7
Figure S3ii… 8
Figure S4(i) 9
Figure S4(ii) 10
Figure S5. .... **Error! Bookmark not defined.**
Figure S6 .... **Error! Bookmark not defined.**
Figure S7 22
Figure S8 .... **Error! Bookmark not defined.**
Figure S9i .... **Error! Bookmark not defined.**
Figure S9ii .... **Error! Bookmark not defined.**
Figure S10a .... **Error! Bookmark not defined.**
Figure S10b .... **Error! Bookmark not defined.**
Figure 11a 30
Figure S11b 31
Figure S12 32
Figure S13 33
Figure S14a 34
Figure S14b.. 34
Figure S15a .... **Error! Bookmark not defined.**5
Figure S15b .... **Error! Bookmark not defined.**
Figure S16 .... **Error! Bookmark not defined.**
Figure S17 .... **Error! Bookmark not defined.**
Figure S18a .... **Error! Bookmark not defined.**
Figure S18b .... **Error! Bookmark not defined.**
Supplementary Tables **40**
Table S1 40
Table S2a 42
Table S2b 43
Table S3 44

Supplementary References……………………………………………………………………..45

Supplementary notes

1. Textural Properties of Scaffolds

1.1 Analysis of textural parameters, including firmness, consistency, cohesiveness, and viscosity index (see Table S1)

The influence of HA concentration on the textural parameters of the CC gels including firmness, cohesiveness and consistency were found to be significant ($p < 0.05$). As the concentration of HA increased, the gels became firmer and more consistent (thicker), hence the adhesiveness and cohesiveness of these gels increased (see later on adhesion). Accordingly, the increase of the textural parameters can be explained by the multiple negatively charged HA subunits allowing them to attract and retain more water (Dovedytis et al. 2020). This further confirms the successful crosslinking by the $CaCl_2$ resulting in strong hydrogel matrices with high water holding capacity as well as ability to remain on the wound long enough while maintaining their physical integrity over a more prolonged period. In contrast the textural parameters of the IPC gels decreased significantly ($p < 0.05$). This is because the crosslinking of SA-G by $CaCl_2$ produced more viscous gels in comparison to IPC HA gels, which further explains the flaky edges of the CC scaffold dressings above.

For the IPC gels, the highest firmness was observed for formulations with highest HA content SA-G:FCOL:HA 1:2:5-IT:PEGDE indicating robust structure while the SA-G:FCOL:HA 1:2:1-IT:PEGDE has the lowest firmness at 0.38 N, indicating a softer material. FCOL:HA 1:3-IT:PEGDE and SA-G:FCOL:HA 1:2:5-IT:PEGDE samples show the highest value at 5.34 N.sec, demonstrating high resistance to flow under stress, in contrast to the SA-G:FCOL:HA 1:2:1-IT:PEGDE with the lowest consistency of 1.06 N.sec, which is less viscous. The cohesiveness ranges from -0.40 N in SA-G:FCOL:HA 1:2:1-IT:PEGDE (less damaging, indicating higher cohesiveness) to -1.95 N in FCOL:HA 3:5-IT:PEGDE (more negative, suggesting lower cohesiveness). Lastly, the viscosity index is highest -0.26 N.sec in SA-G:FCOL:HA 1:2:1-IT:PEGDE, showing lower resistance to flow, and lowest -1.71 N.sec in SA-G:FCOL:HA 1:2:5-IT:PEGDE, indicating higher resistance to flow. Furthermore, the consistency of the IT-PEGDE crosslinked gels decreased upon loading the BSA. This could be due to interruption of the non-covalent weaker crosslinks thus disrupting the polymeric matrix. According to Lenormand and Vincent (2010), HA is able to form complexes with various proteins such as BSA through electrostatic interaction and this was reflected in the decreased

water retention capacity of the resulting scaffold dressings upon BSA loading compared to scaffold dressings obtained from the blank gels. This further indicates that the pH of the gel may be above the isoelectric point of BSA, increasing the charge on the protein, initiating electrostatic repulsion and stabilizing the BSA molecule and preventing it from aggregation (Lenormand and Vincent 2011)(Li et al., 2016).

2. GPC Analysis of Crosslinked Scaffolds

GPC chromatogram showing molecular weight distribution of crosslinked and non-crosslinked gels (Figure S2).

2.1 Gel permeation chromatography (GPC)

To confirm the crosslinking of HA within the formulations, the molecular size of plain and IPC HA gels was monitored using an Agilent 1100 series gel permeation chromatography (GPC) analysis system equipped with an isocratic pump and a refractive index detector. About 2 mg/ml solutions were prepared by dissolving the samples in 0.1M $NaNO_3$ buffer pH 7.4 and eluted through an ultra-hydrogel 500 Column, (500 Å, 7.8 mm x 300 mm10 µm) at a flow rate of 1 ml/min at 35˚C using the same buffer as mobile phase. Collected GPC data was analyzed based on the increase in molecular size using the Agilent ChemStation software. It is important to note that the gel samples were prepared 24 h before the experiment and stored at 4˚C overnight to allow complete hydration of the samples.

2.2 Gel permeation chromatography (GPC)

The relationship between the different molecular weights of HA and their physiological function is one of the key features of this biopolymer. High molecular weight HA grades greater than 1000 kDa are known for their anti-angiogenic, immunosuppressive and anti-inflammatory effects while the lower molecular weight grades possess pro-inflammatory, proangiogenic and immunostimulatory effects [K. Valachová and L. Šoltés, 2021]. In this study HA was crosslinked with IT:PEGDE and the crosslinking effects on the molecular weight of HA were studied using GPC on both NC and IPC HA gels. The estimation of molecular weight of HA-based formulations with no HA calibration standards has been attempted previously [Shanmuga Doss et al., 2017], however, huge variations were observed, and it has been reported that these estimations often led to wrong estimation of the molecular size.

In GPC, separation is based on the size and not chemistry and because the polymer is dissolved in the solvent, the polymer chains assume a coil conformation in solution and behave like spheres (beads) during separation. Furthermore, the size of the spheres depends on the molecular weight which means higher molecular weight polymers will form larger spheres.

The larger beads that do not fit in the pores of the gel stationary phase get carried by the mobile phase and are eluted first while the smaller spheres or lower molecular weight polymers take longer to elute from the column. The chromatographic results in (Figure S2) indicate distinct molecular weight fractions, each represented by the observed peaks. The first peak eluted between 3 and 6 minutes and characterized by a sharp rise and fall in intensity. This peak represents hyaluronic acid's higher molecular weight fraction, which elutes earlier due to its limited penetration into the pores of the gel matrix used in SEC. The sharpness of this peak suggests a uniform molecular size distribution, indicating a successful crosslinking process where a significant proportion of the hyaluronic acid molecules attained similar, higher molecular weights. The first peak was observed eluting between 3 and 6 minutes and characterized by a sharp rise and fall in intensity. Quantitative analysis of the areas under the peaks would further aid in understanding the proportion of each molecular weight fraction, providing insights essential for optimizing the crosslinking process to achieve the desired product specifications. The composite scaffolds were also evaluated with GPC however it was hard to separate the overlapping peaks.

Supplementary Figures

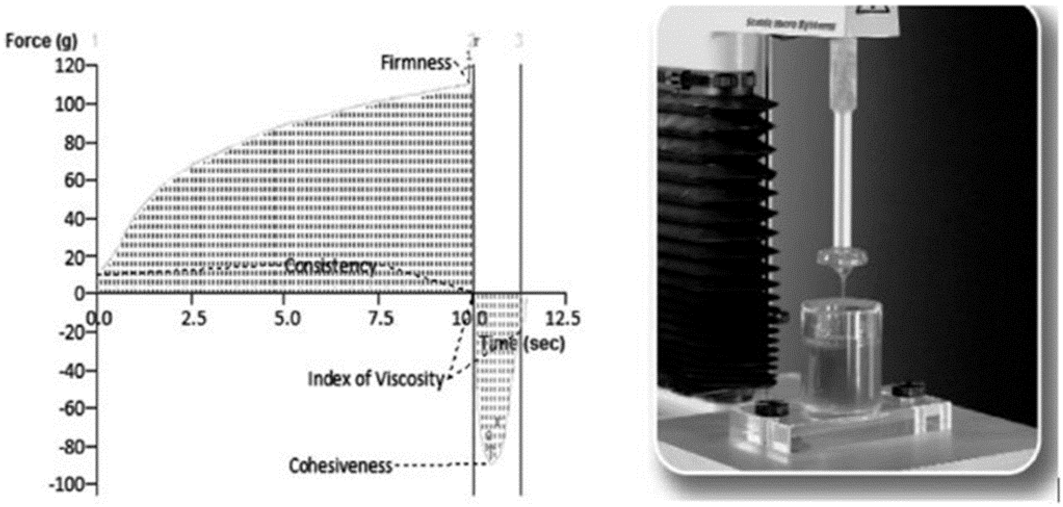

Figure S1. A typical experimental setup for back extrusion test and a time-force curve displaying the textural properties. Where the maximum positive force is the sample firmness (g) the positive area is the consistency (gs), the maximum negative force is the cohesiveness (g) and the maximum negative peak, and the index of viscosity is shown by the negative area (gs) (Tuğcu-Demiröz 2017)

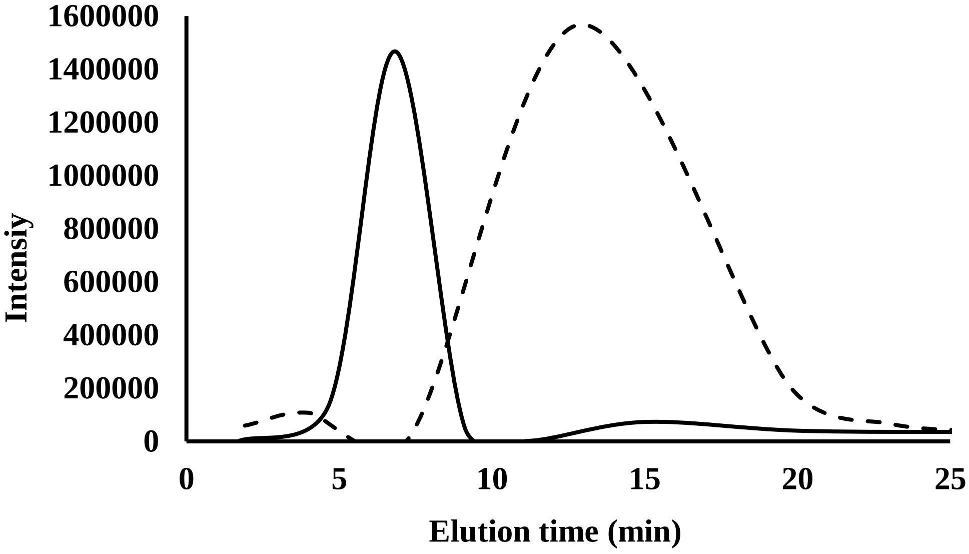

Figure S2. Representative GPC chromatogram for crosslinked (solid line) and non-crosslinked gels (broken line), clearly showing shifts in elution time after crosslinking compared to the native HA.

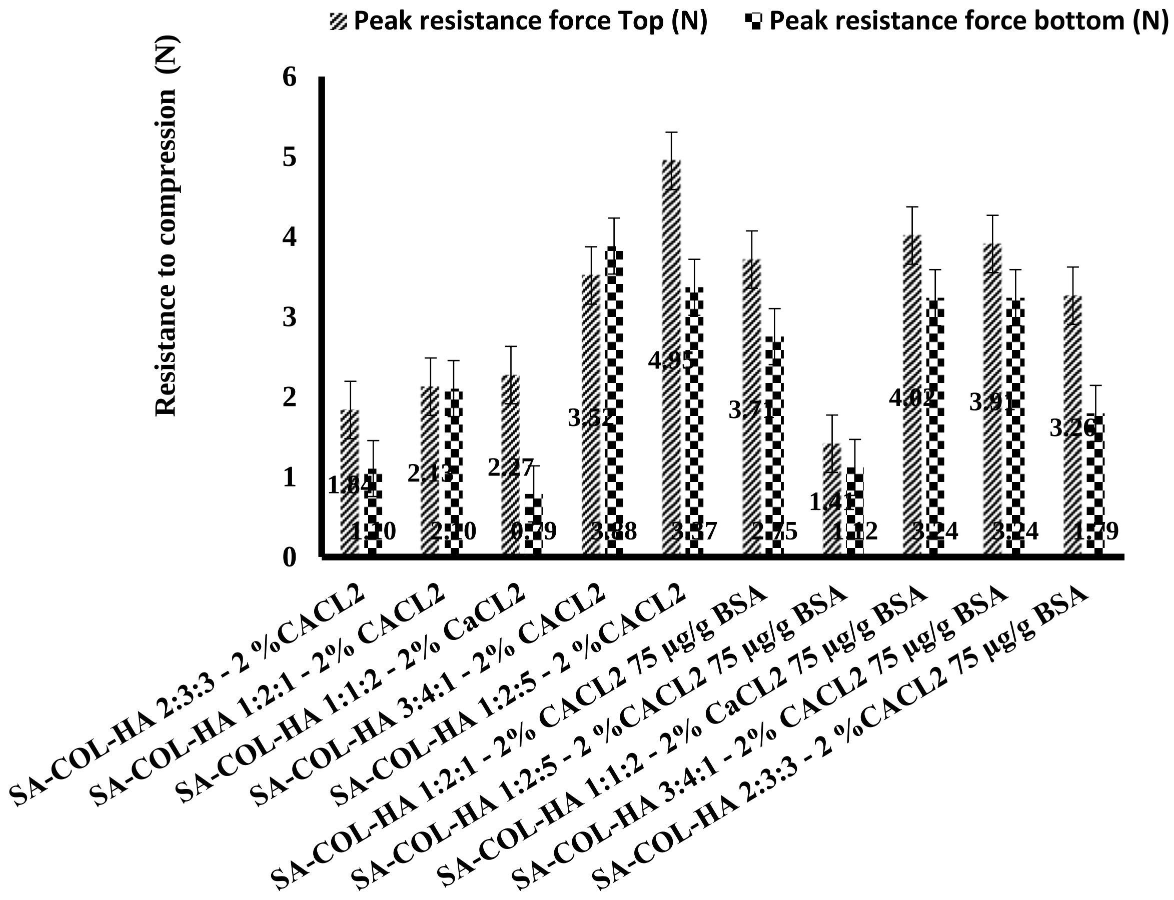

Figure S3i Hardness profiles of a) Top and the bottom of the scaffold dressings for composite SA-G:FCOL:HA, b) The top and bottom of BSA loaded formulations of the scaffold dressings for composite SA-G:FCOL:HA, Data are shown as mean ± standard deviation (n = 3 ± SD). Statistical differences are shown with ns denotes $p > 0.05$

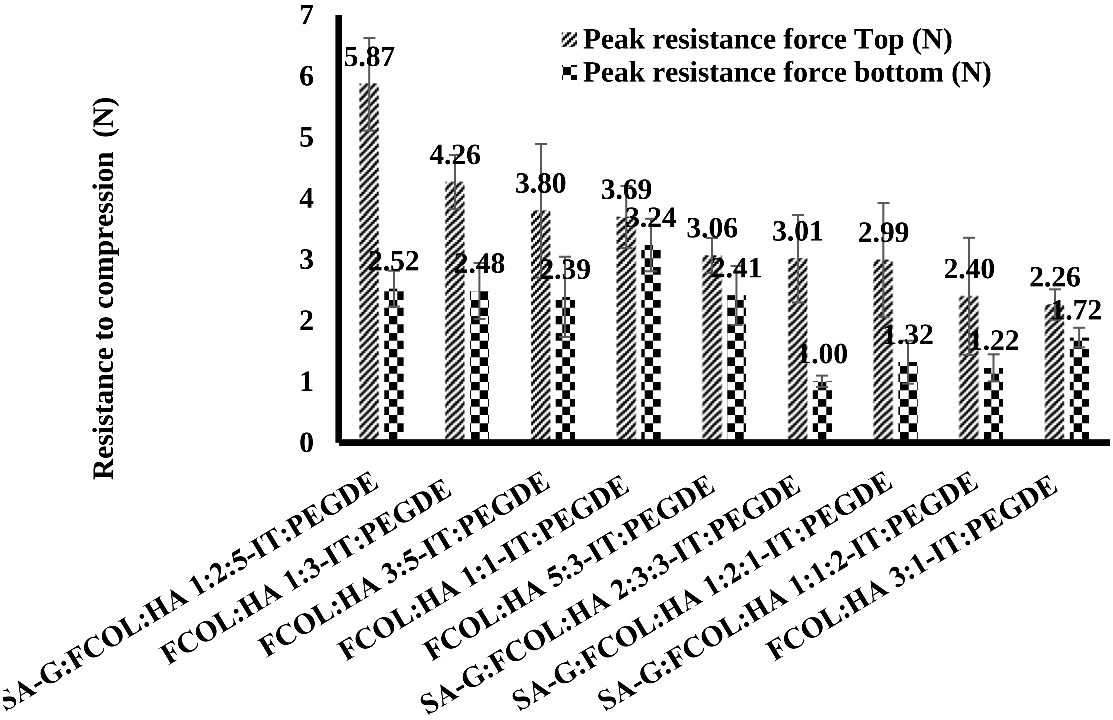

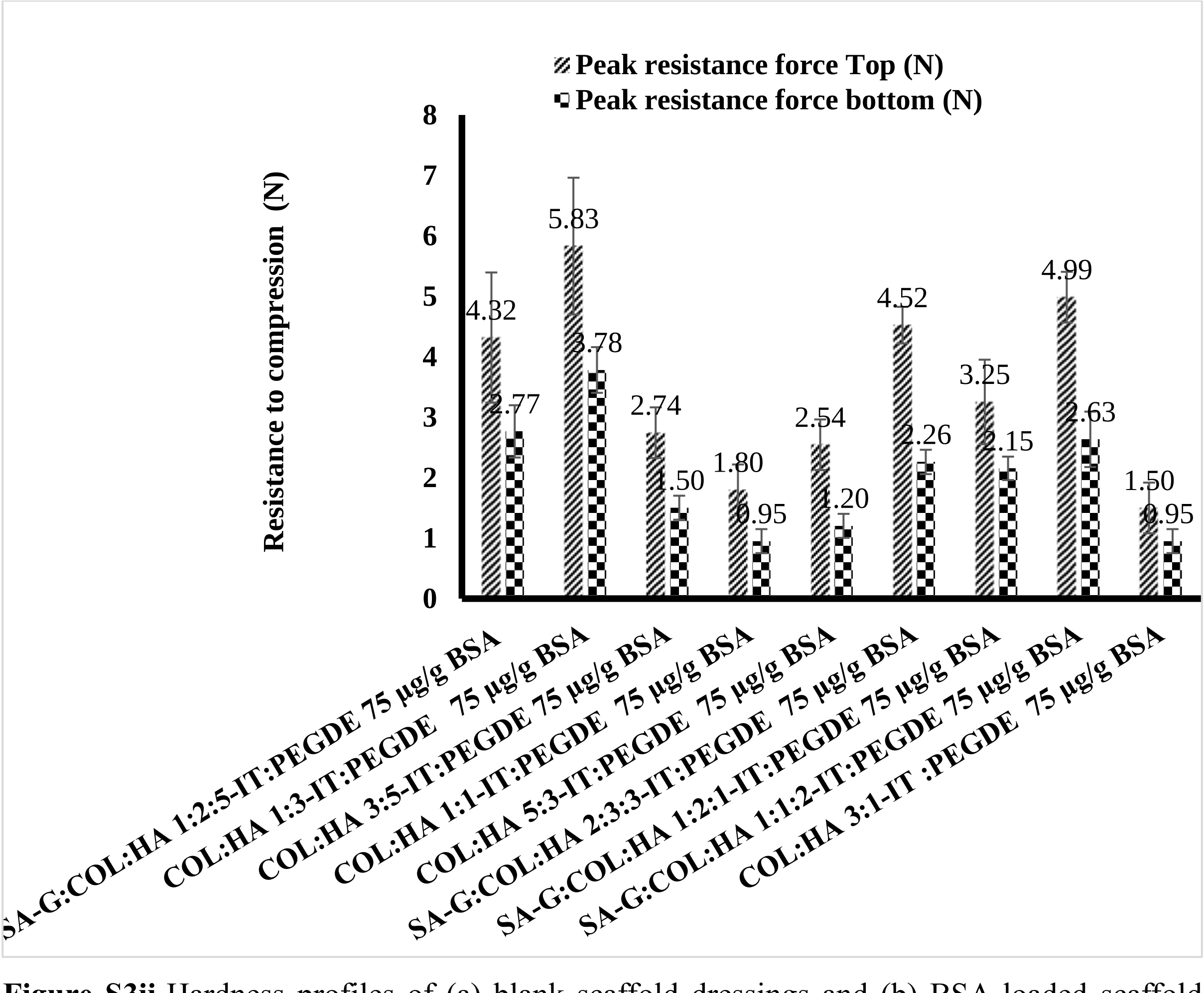

Figure S3ii Hardness profiles of (a) blank scaffold dressings and (b) BSA loaded scaffold dressings obtained from 2% COL:HA gels crosslinked with IT-PEGDE (n = 3 ± SD)

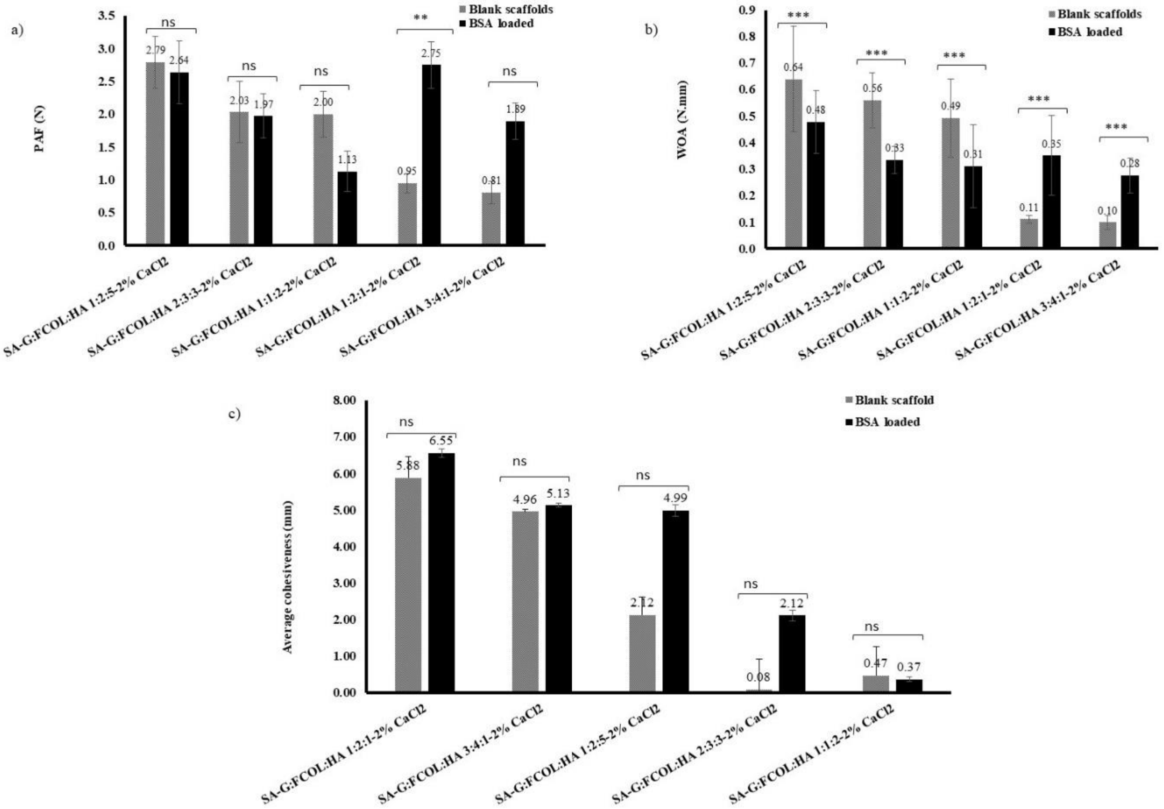

Figure S4i Adhesive profiles of blank and BSA loaded 2% SA-G:FCOL:HA scaffold dressings crosslinked with 2% $CaCl_2$ a) peak force of adhesion, b) work of adhesion, c) cohesiveness. Data are shown as mean ± standard deviation ($n = 3$). Statistical differences are shown with ns, **, or *** denoting $p > 0.05$, $p < 0.01$, or $p < 0.001$, respectively

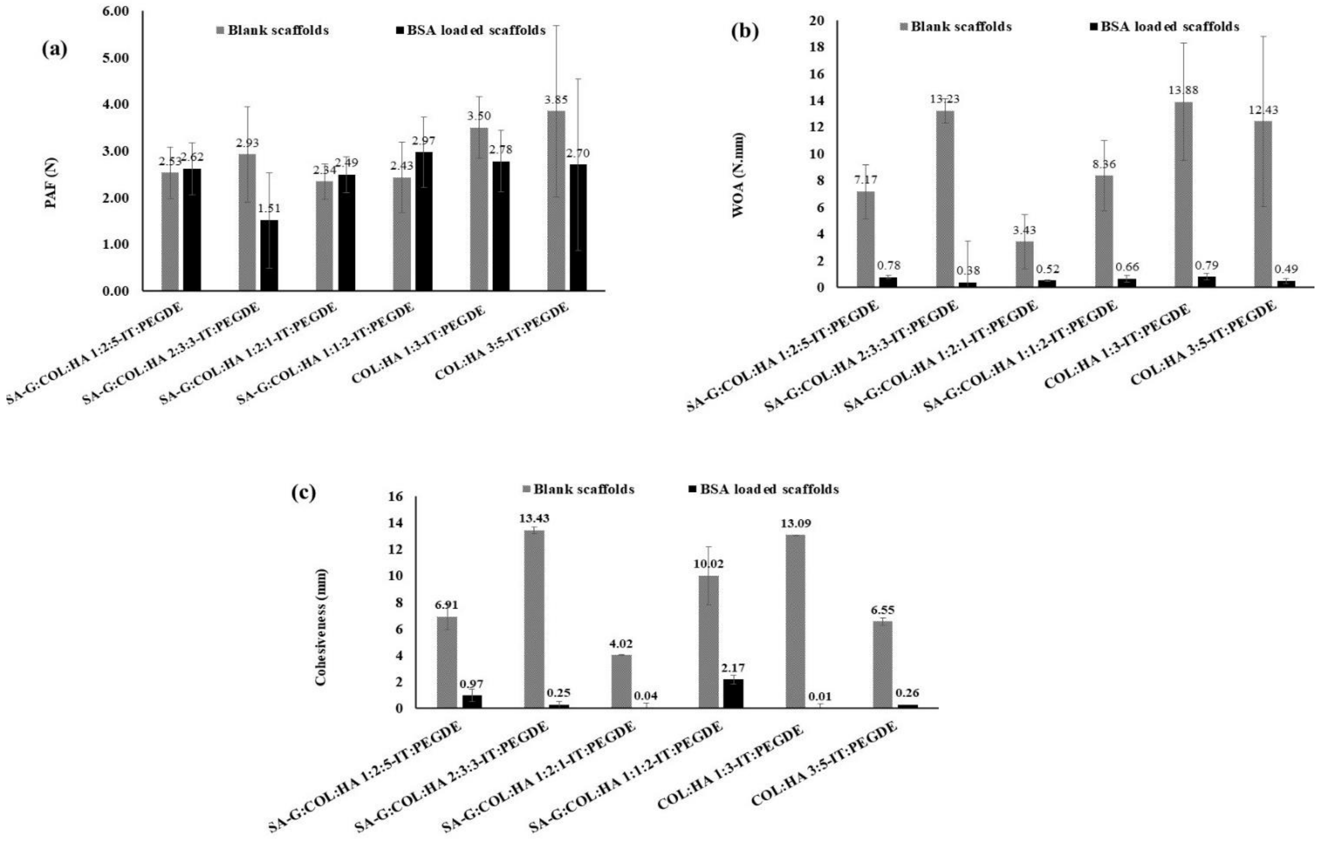

Figure S4ii Adhesive profiles for IPC scaffold dressings showing the (a) PAF, (b) WOA and (c) cohesiveness comparisons between the blank and BSA loaded formulations. The difference in the cohesiveness of the SA-G:FCOL:HA crosslinked with IT-PEGDE were statistically different $p > 0.05$.

a)

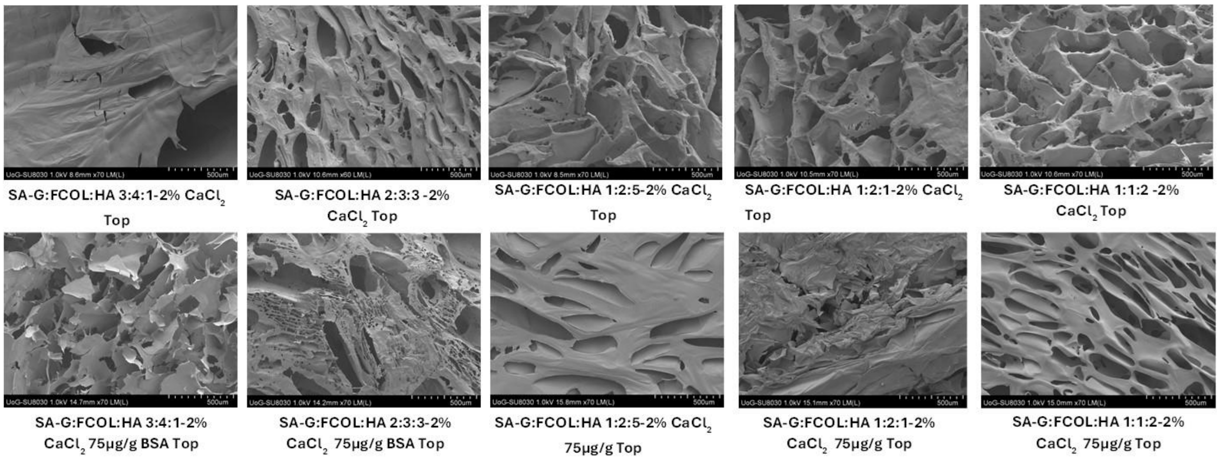
SA-G:FCOL:HA 3:4:1-2% $CaCl_2$ Top
SA-G:FCOL:HA 2:3:3 -2% $CaCl_2$ Top
SA-G:FCOL:HA 1:2:5-2% $CaCl_2$ Top
SA-G:FCOL:HA 1:2:1-2% $CaCl_2$ Top
SA-G:FCOL:HA 1:1:2 -2% $CaCl_2$ Top
SA-G:FCOL:HA 3:4:1-2% $CaCl_2$ 75µg/g BSA Top
SA-G:FCOL:HA 2:3:3-2% $CaCl_2$ 75µg/g BSA Top
SA-G:FCOL:HA 1:2:5-2% $CaCl_2$ 75µg/g Top
SA-G:FCOL:HA 1:2:1-2% $CaCl_2$ 75µg/g Top
SA-G:FCOL:HA 1:1:2-2% $CaCl_2$ 75µg/g Top

b)

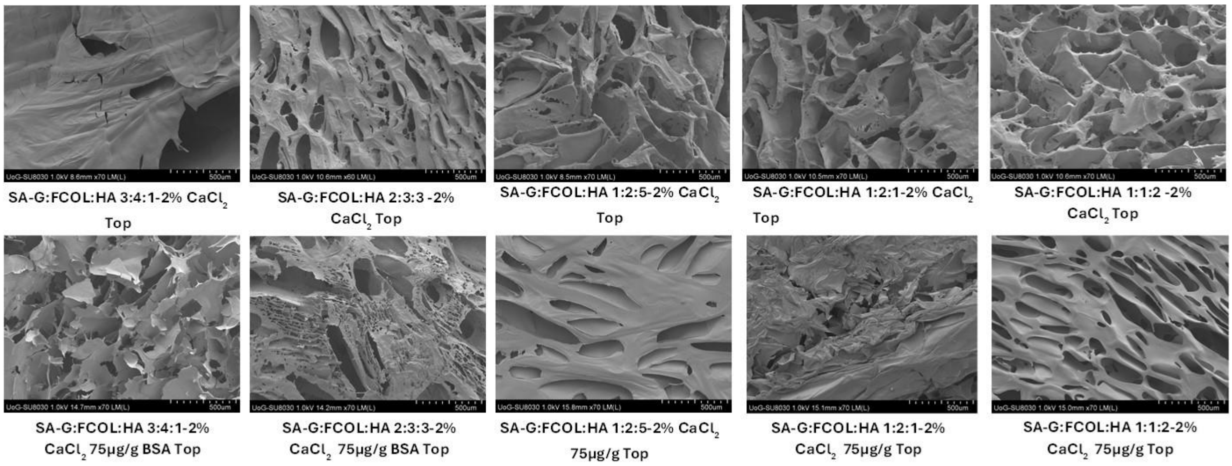
SA-G:FCOL:HA 3:4:1-2% $CaCl_2$ Top
SA-G:FCOL:HA 2:3:3 -2% $CaCl_2$ Top
SA-G:FCOL:HA 1:2:5-2% $CaCl_2$ Top
SA-G:FCOL:HA 1:2:1-2% $CaCl_2$ Top
SA-G:FCOL:HA 1:1:2 -2% $CaCl_2$ Top
SA-G:FCOL:HA 3:4:1-2% $CaCl_2$ 75µg/g BSA Top
SA-G:FCOL:HA 2:3:3-2% $CaCl_2$ 75µg/g BSA Top
SA-G:FCOL:HA 1:2:5-2% $CaCl_2$ 75µg/g Top
SA-G:FCOL:HA 1:2:1-2% $CaCl_2$ 75µg/g Top
SA-G:FCOL:HA 1:1:2-2% $CaCl_2$ 75µg/g Top

c)

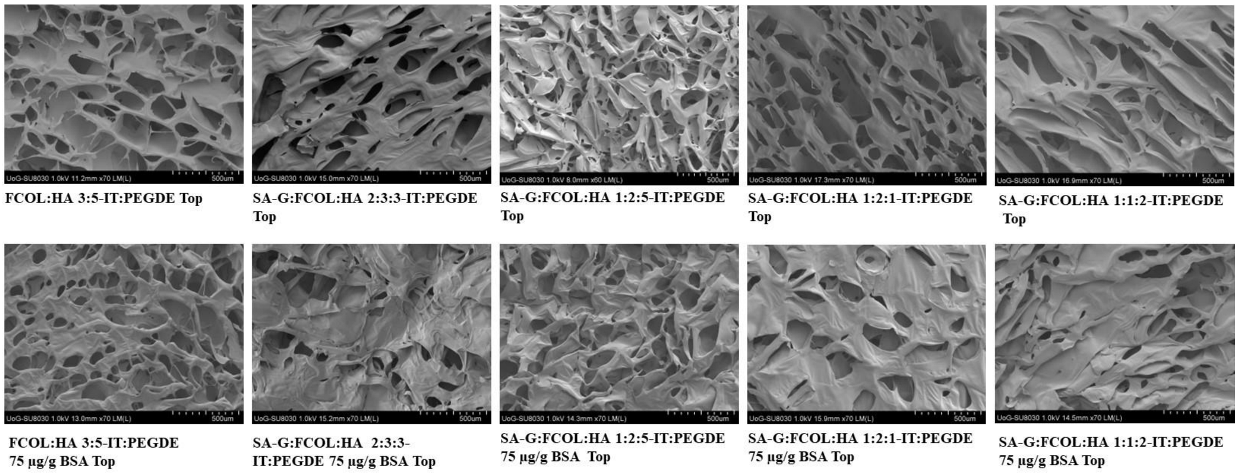

d)

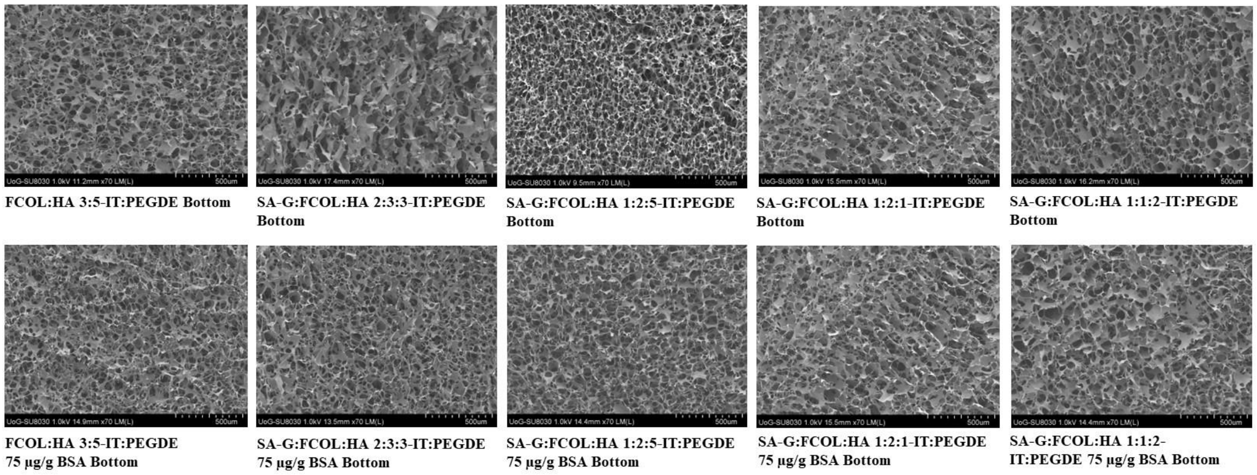

Figure S5. SEM images optimized blank and BSA loaded CC and IPC scaffolds

a)

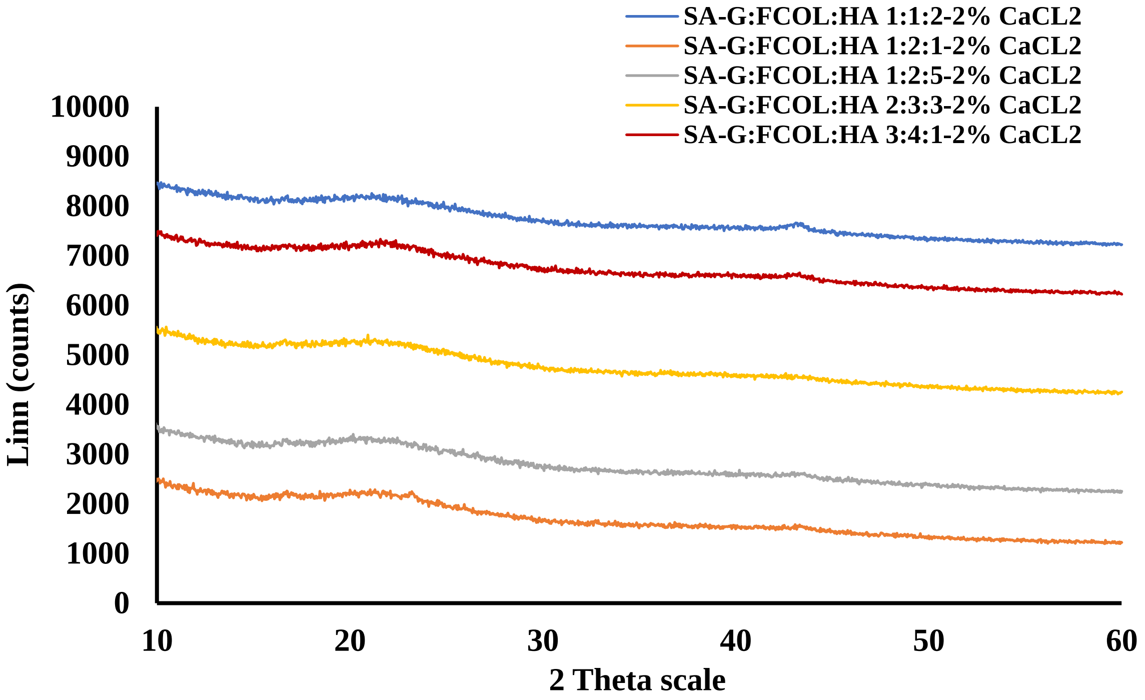
SA-G:FCOL:HA 1:1:2-2% CaCL2
SA-G:FCOL:HA 1:2:1-2% CaCL2
SA-G:FCOL:HA 1:2:5-2% CaCL2
SA-G:FCOL:HA 2:3:3-2% CaCL2
SA-G:FCOL:HA 3:4:1-2% CaCL2
Linn (counts)
0
1000
2000
3000
4000
5000
6000
7000
8000
9000
10000
10
20
30
40
50
60
2 Theta scale

b)

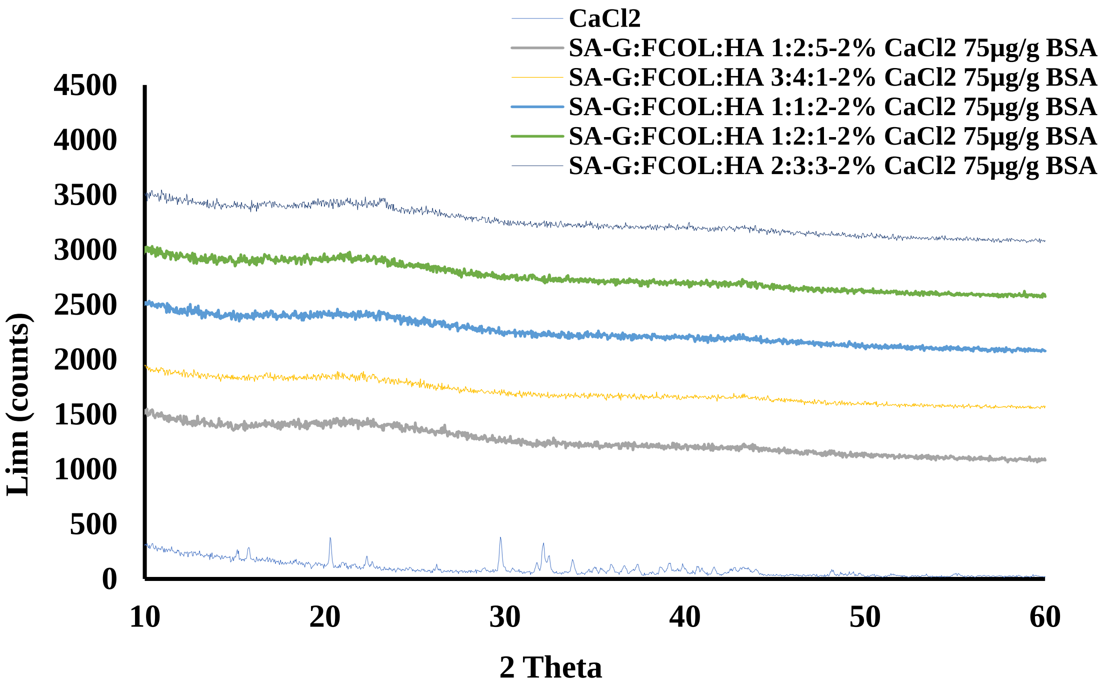
CaCl2
SA-G:FCOL:HA 1:2:5-2% CaCl2 75μg/g BSA
SA-G:FCOL:HA 3:4:1-2% CaCl2 75μg/g BSA
SA-G:FCOL:HA 1:1:2-2% CaCl2 75μg/g BSA
SA-G:FCOL:HA 1:2:1-2% CaCl2 75μg/g BSA
SA-G:FCOL:HA 2:3:3-2% CaCl2 75μg/g BSA
Linn (counts)
0
500
1000
1500
2000
2500
3000
3500
4000
4500
10
20
30
40
50
60
2 Theta

c)

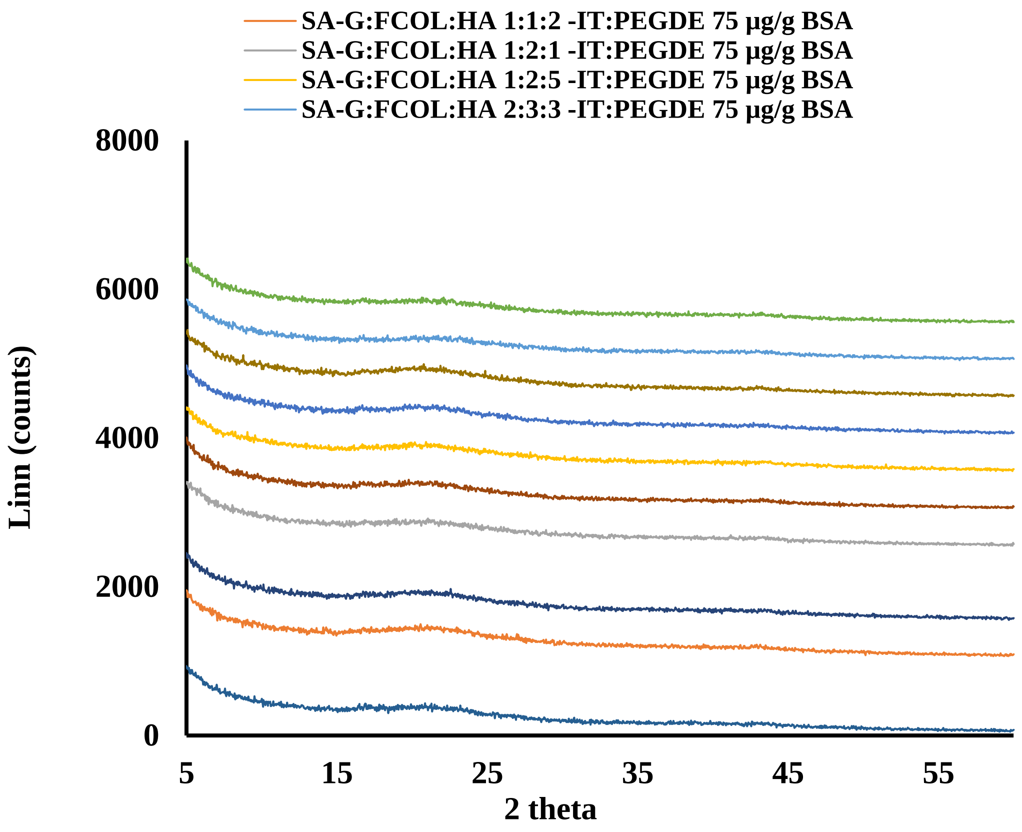

d)

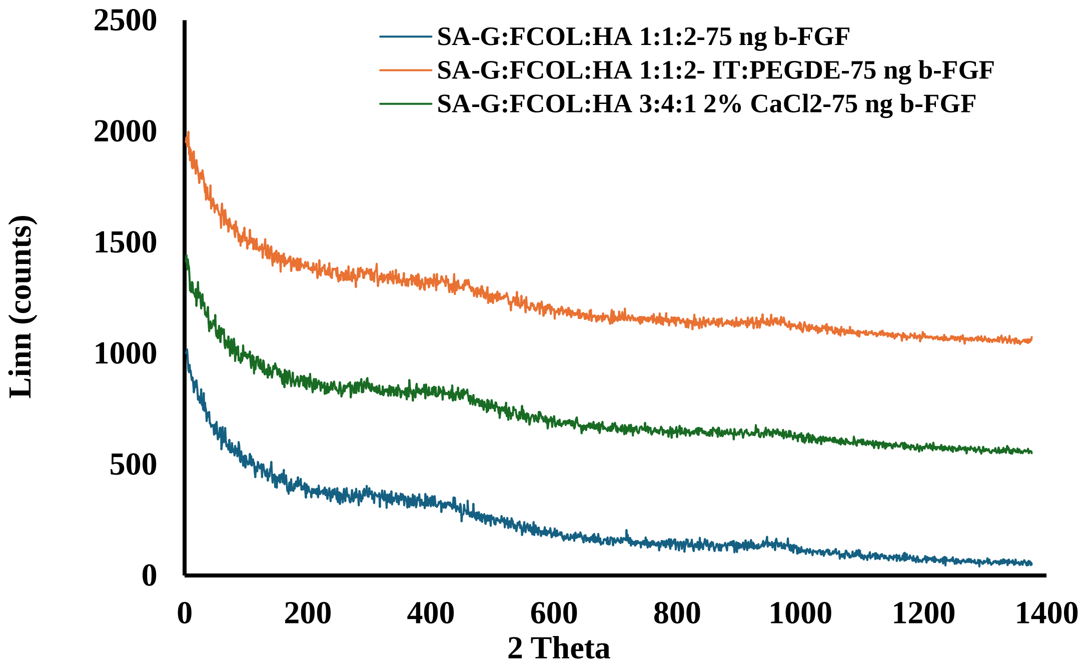

Figure S6 XRD diffractograms for blank, BSA and b-FGF loaded CC and IPC SA-G:FCOL:HA scaffolds.

a)

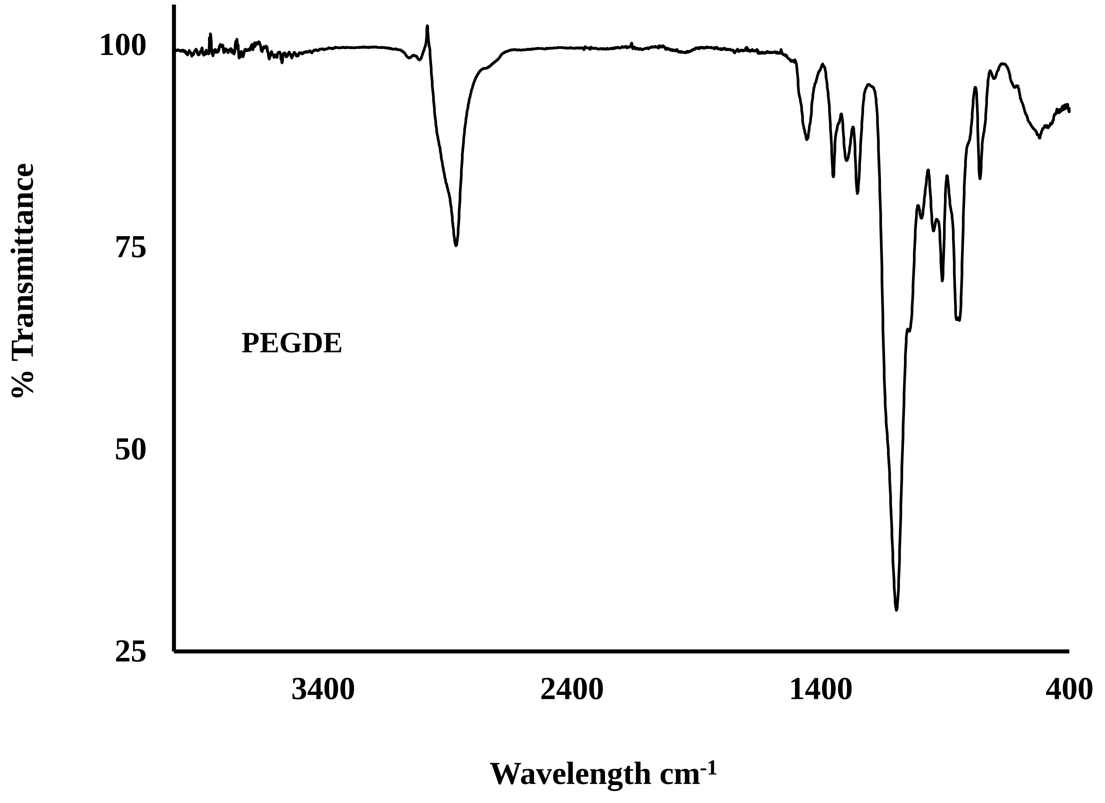

100
75
50
25
% Transmittance
PEGDE
3400
2400
1400
400
Wavelength cm-1

b)

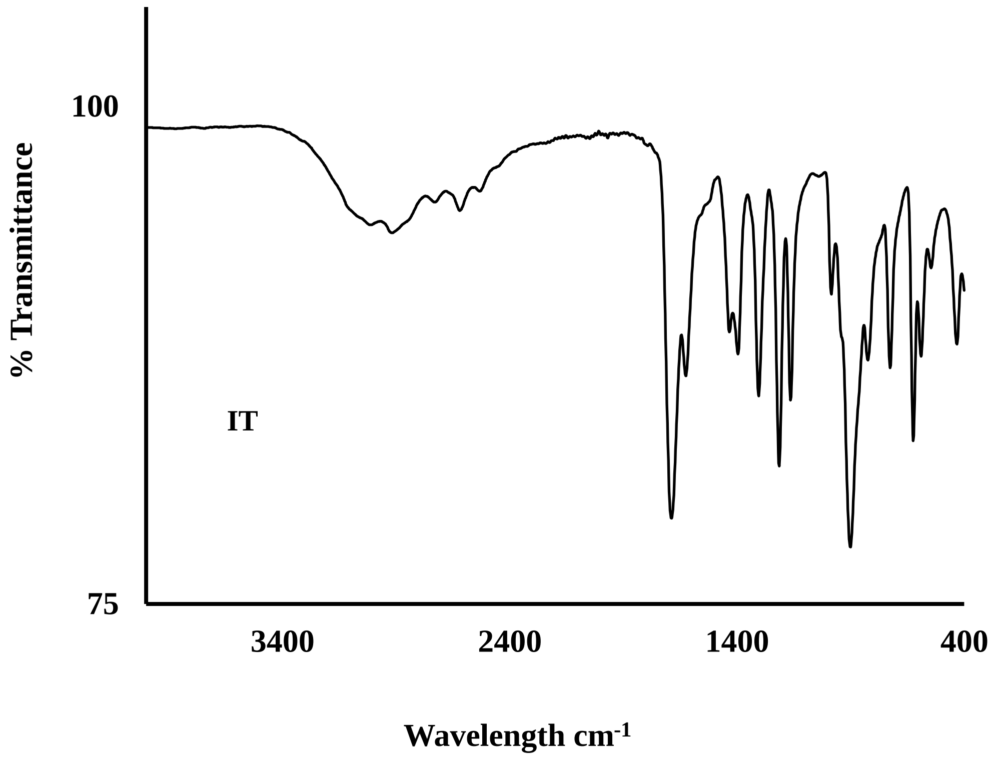

100
75
% Transmittance
IT
3400
2400
1400
400
Wavelength cm-1

c)

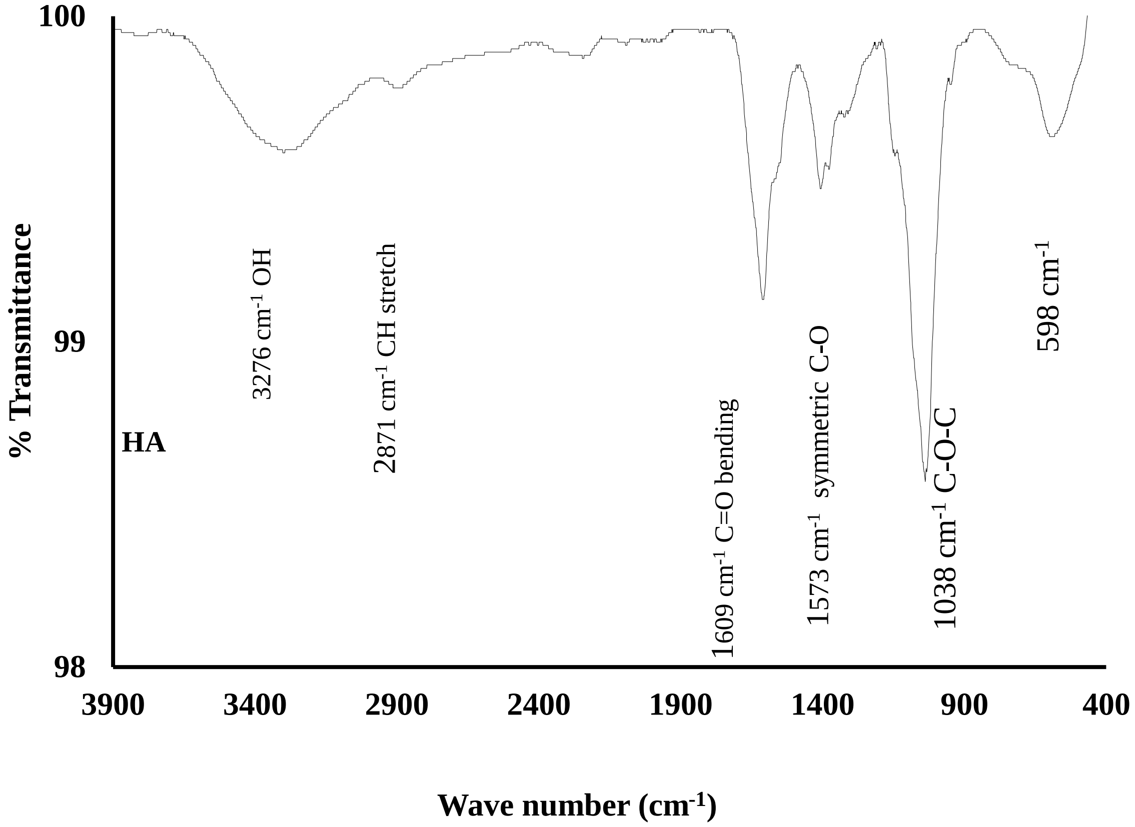
100
99
98
% Transmittance
HA
3276 cm-1 OH
2871 cm-1 CH stretch
1609 cm-1 C=O bending
1573 cm-1 symmetric C-O
1038 cm-1 C-O-C
598 cm-1
3900
3400
2900
2400
1900
1400
900
400
Wave number (cm-1)

d)

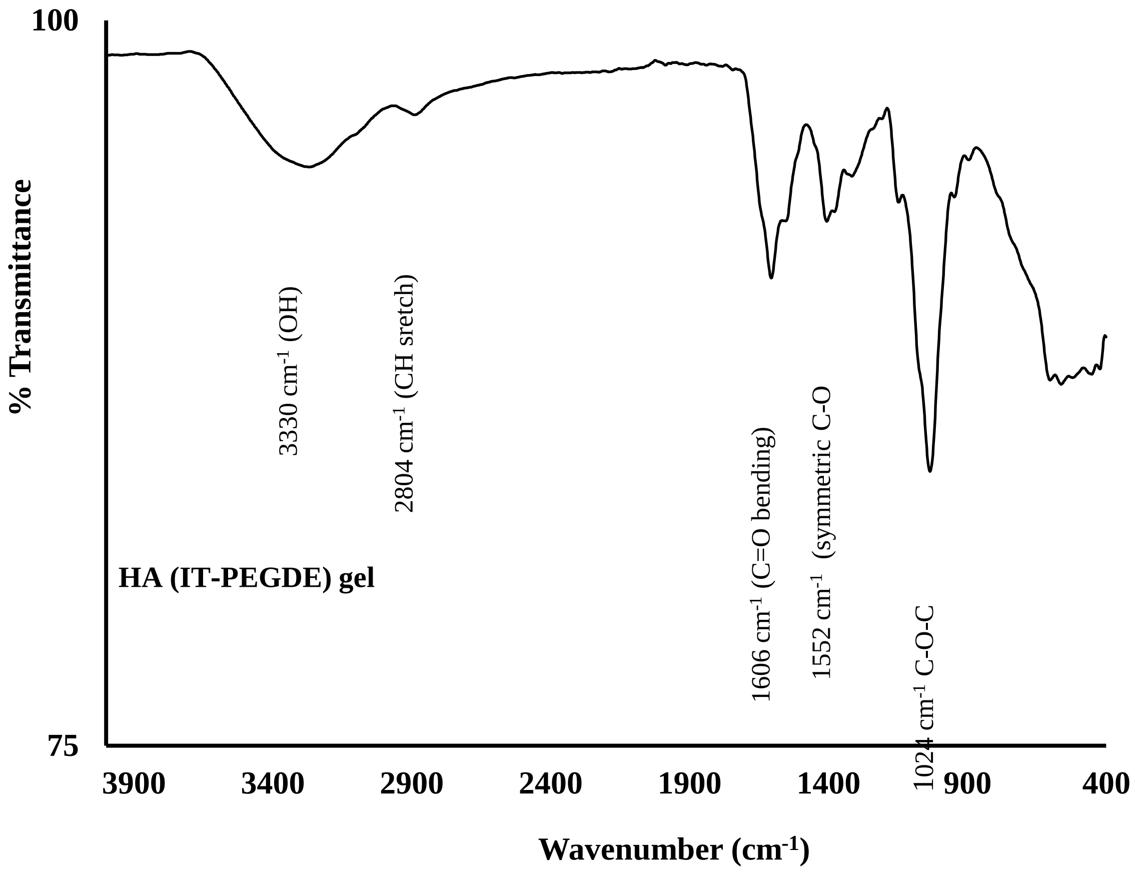
100
75
% Transmittance
HA (IT-PEGDE) gel
3330 cm-1 (OH)
2804 cm-1 (CH sretch)
1606 cm-1 (C=O bending)
1552 cm-1 (symmetric C-O
1024 cm-1 C-O-C
3900
3400
2900
2400
1900
1400
900
400
Wavenumber (cm-1)

e)

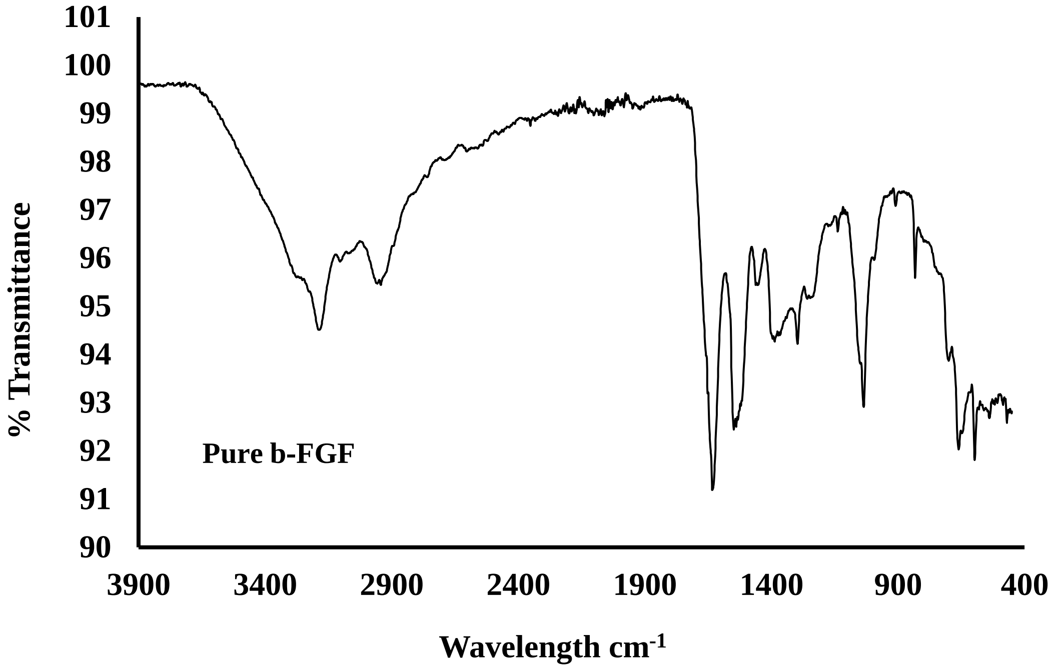

101
100
99
98
97
96
95
94
93
92
91
90
% Transmittance
Pure b-FGF
3900
3400
2900
2400
1900
1400
900
400
Wavelength cm-1

f)

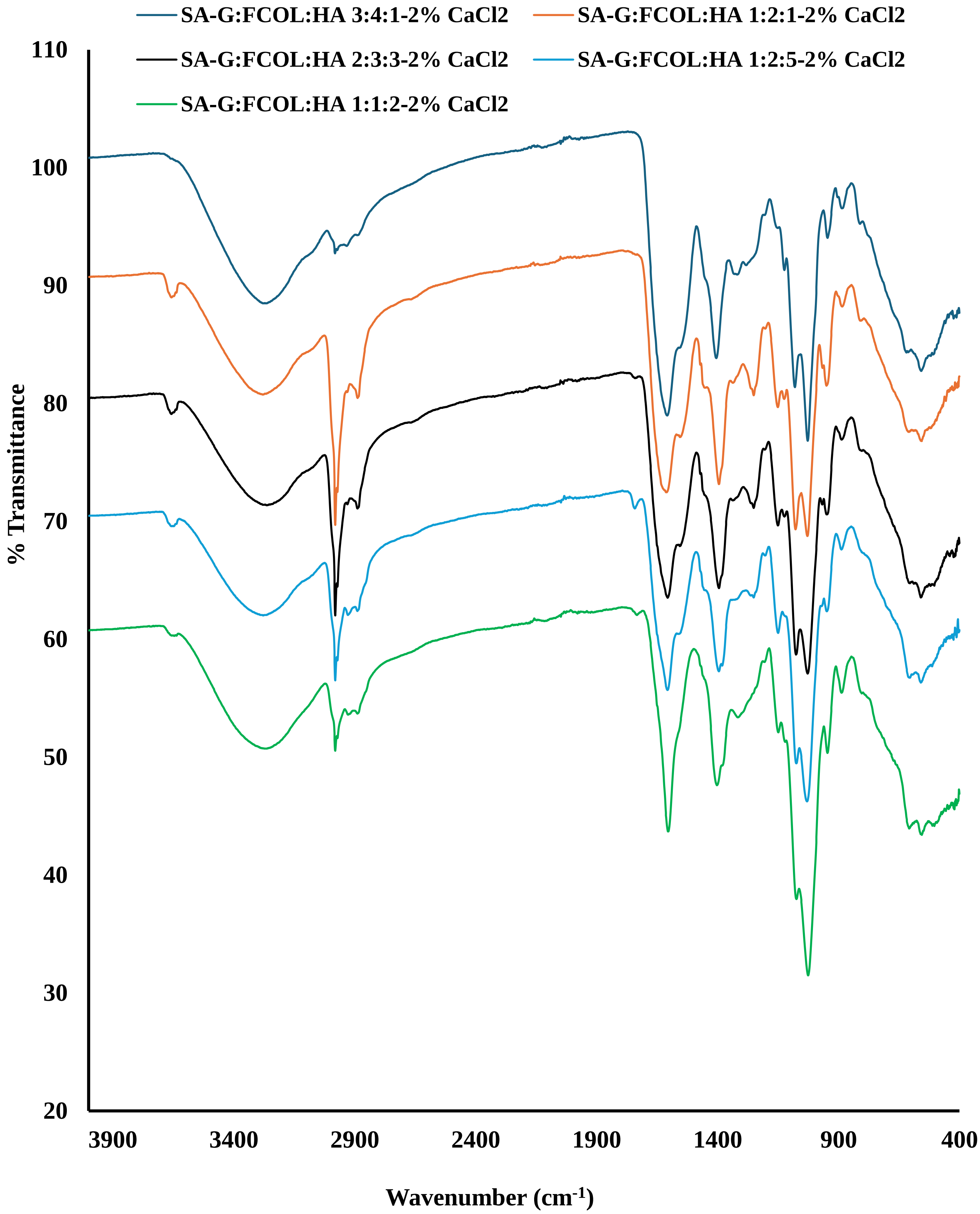

SA-G:FCOL:HA 3:4:1-2% CaCl2
SA-G:FCOL:HA 1:2:1-2% CaCl2
SA-G:FCOL:HA 2:3:3-2% CaCl2
SA-G:FCOL:HA 1:2:5-2% CaCl2
SA-G:FCOL:HA 1:1:2-2% CaCl2
110
100
90
80
70
60
50
40
30
20
% Transmittance
3900
3400
2900
2400
1900
1400
900
400
Wavenumber (cm-1)

g)

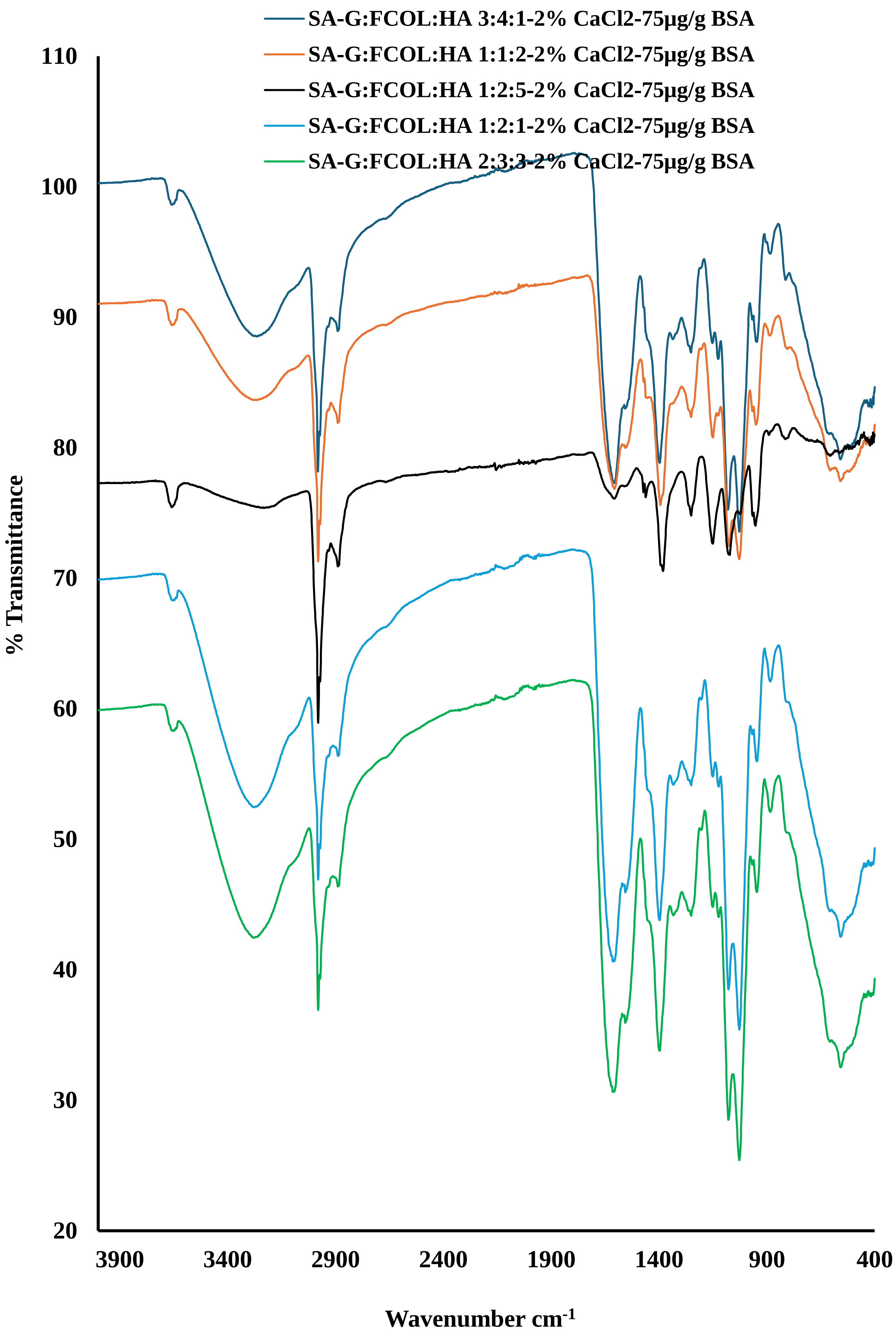
SA-G:FCOL:HA 3:4:1-2% CaCl2-75µg/g BSA
SA-G:FCOL:HA 1:1:2-2% CaCl2-75µg/g BSA
SA-G:FCOL:HA 1:2:5-2% CaCl2-75µg/g BSA
SA-G:FCOL:HA 1:2:1-2% CaCl2-75µg/g BSA
SA-G:FCOL:HA 2:3:3-2% CaCl2-75µg/g BSA
110
100
90
80
70
60
50
40
30
20
% Transmittance
3900
3400
2900
2400
1900
1400
900
400
Wavenumber cm-1

h)

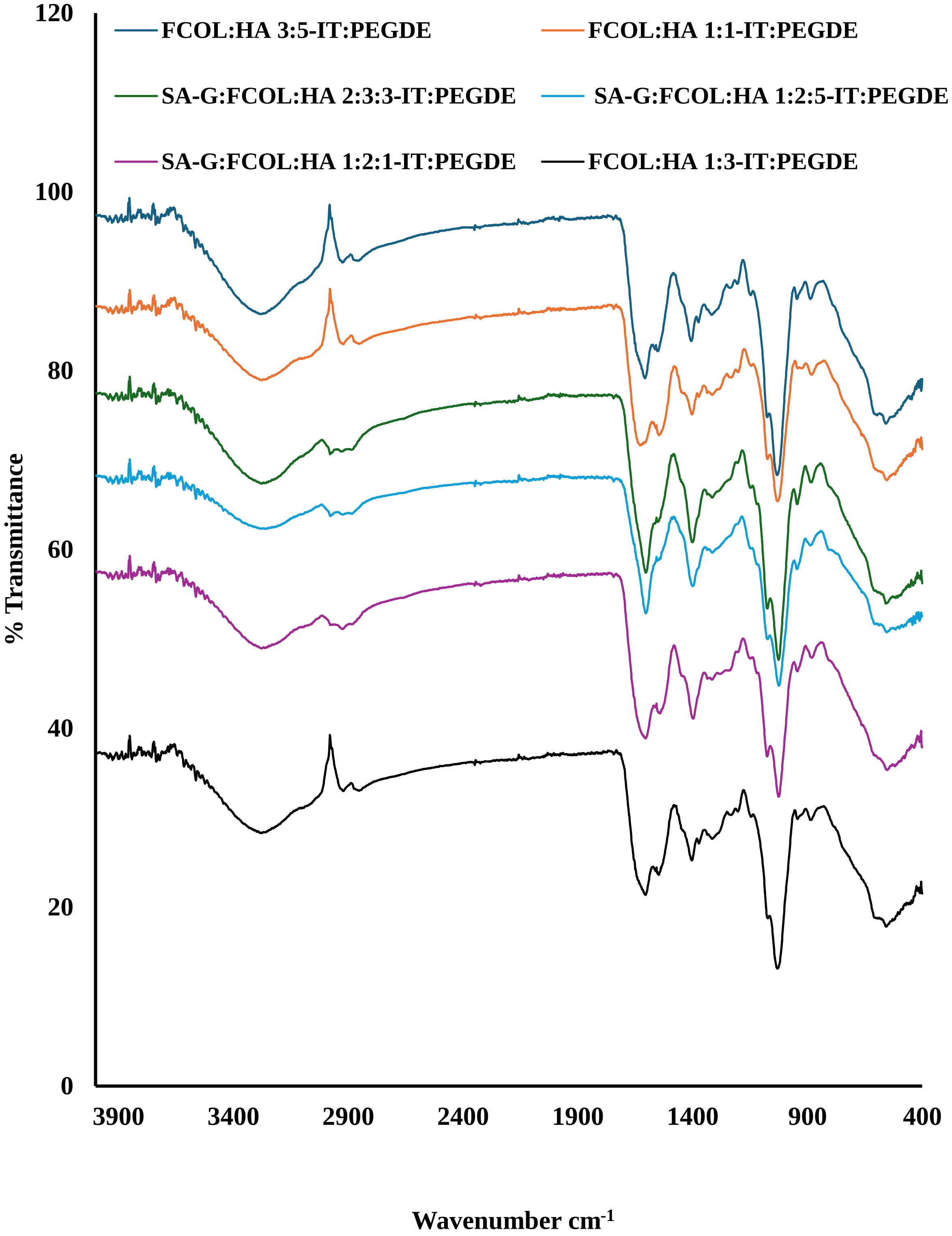

FCOL:HA 3:5-IT:PEGDE
FCOL:HA 1:1-IT:PEGDE
SA-G:FCOL:HA 2:3:3-IT:PEGDE
SA-G:FCOL:HA 1:2:5-IT:PEGDE
SA-G:FCOL:HA 1:2:1-IT:PEGDE
FCOL:HA 1:3-IT:PEGDE
% Transmittance
120
100
80
60
40
20
0
3900
3400
2900
2400
1900
1400
900
400
Wavenumber cm-1

i)

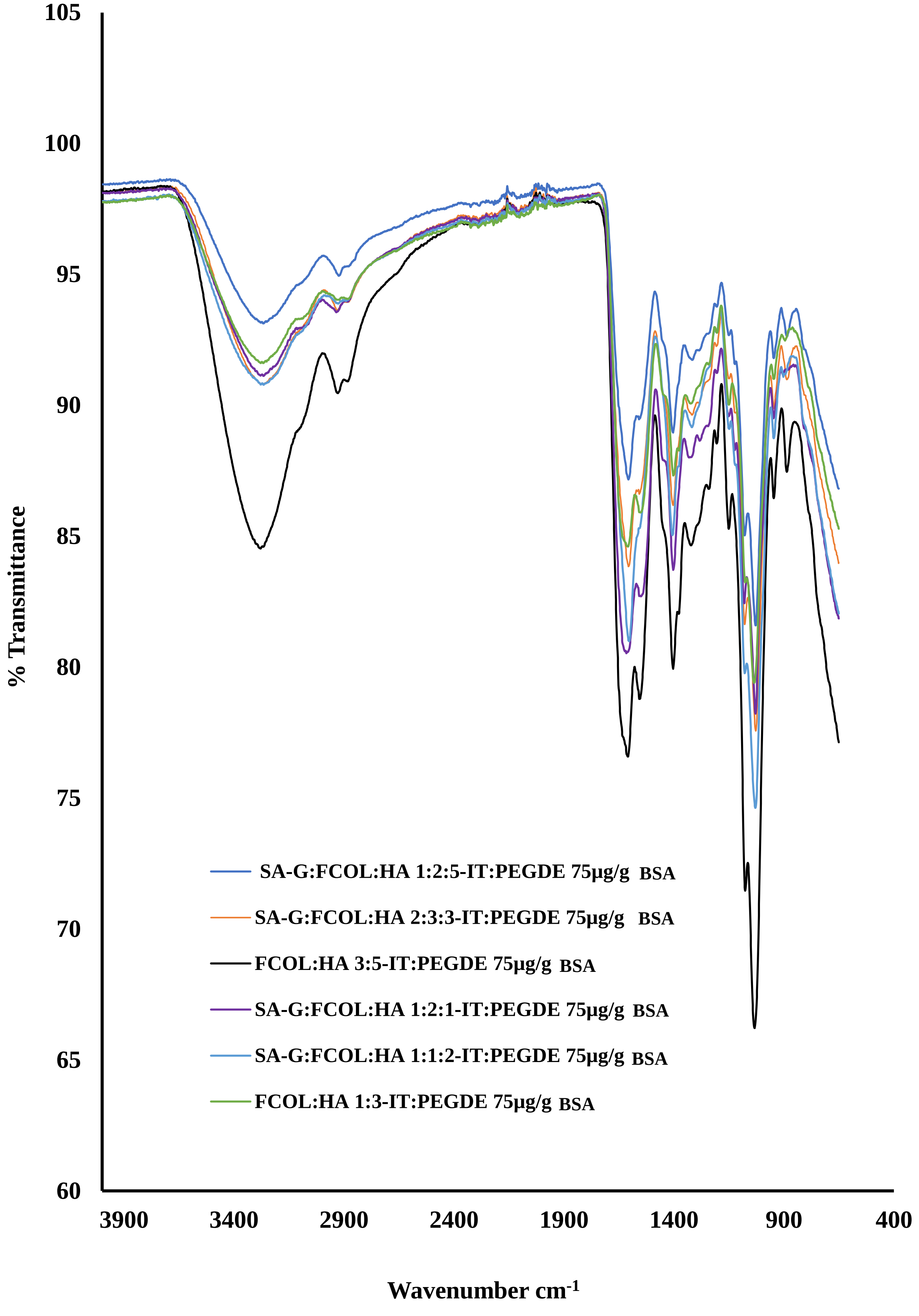

105
100
95
90
85
80
75
70
65
60
% Transmittance
3900
3400
2900
2400
1900
1400
900
400
Wavenumber cm-1
SA-G:FCOL:HA 1:2:5-IT:PEGDE 75µg/g BSA
SA-G:FCOL:HA 2:3:3-IT:PEGDE 75µg/g BSA
FCOL:HA 3:5-IT:PEGDE 75µg/g BSA
SA-G:FCOL:HA 1:2:1-IT:PEGDE 75µg/g BSA
SA-G:FCOL:HA 1:1:2-IT:PEGDE 75µg/g BSA
FCOL:HA 1:3-IT:PEGDE 75µg/g BSA

j)

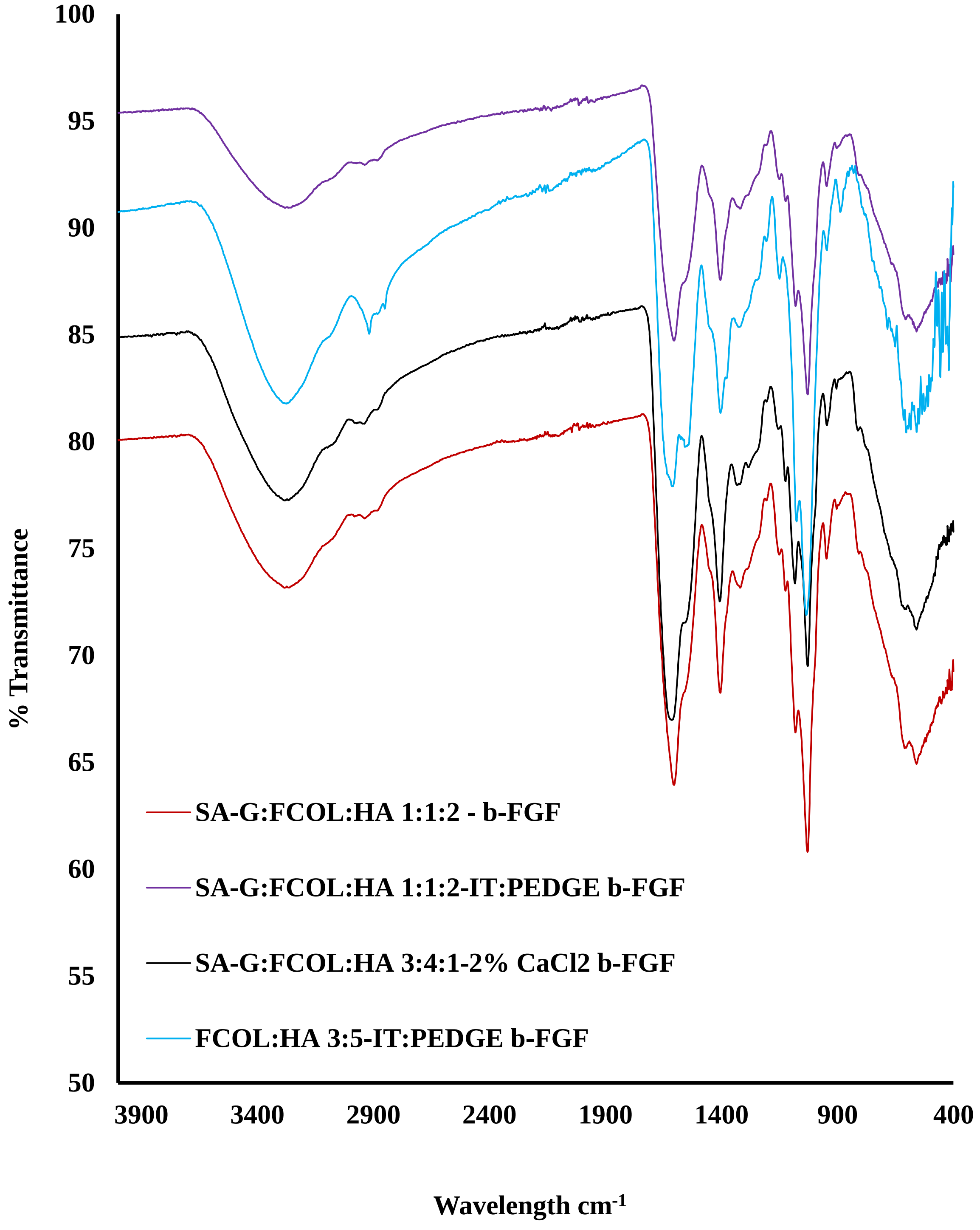

Figure S7 FTIR spectra of pure starting materials used to prepare the scaffolds. FTIR spectra for blank and BSA and b-FGF loaded crosslinked scaffolds comprising SA, FCOL and HA.

a)

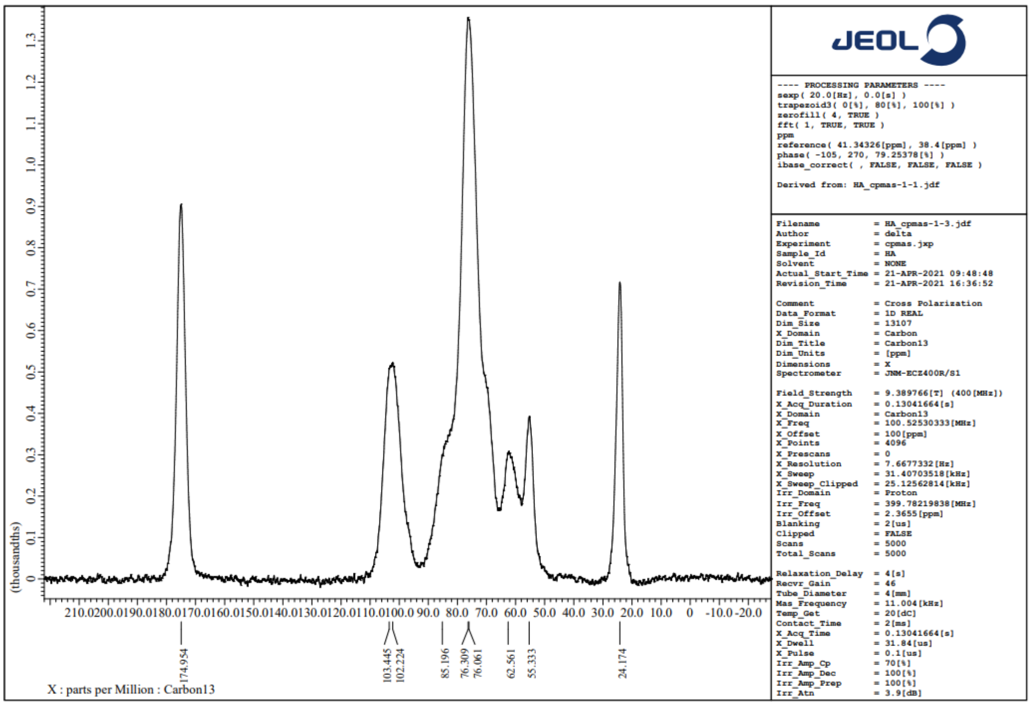

(thousandths)
X : parts per Million : Carbon13
174.954
103.445
102.224
85.196
76.309
76.061
62.561
55.333
24.174
---- PROCESSING PARAMETERS ----
sexp(20.0[Hz], 0.0[s])
trapezoid3(0[%], 80[%], 100[%])
zerofill(4, TRUE)
fft(1, TRUE, TRUE)
ppm
reference(41.34326[ppm], 38.4[ppm])
phase(-105, 270, 79.25378[%])
ibase_correct(, FALSE, FALSE, FALSE)
Derived from: HA_cpmas-1-1.jdf
Filename = HA_cpmas-1-3.jdf
Author = delta
Experiment = cpmas.jxp
Sample_Id = HA
Solvent = NONE
Actual_Start_Time = 21-APR-2021 09:48:48
Revision_Time = 21-APR-2021 16:36:52
Comment = Cross Polarization
Data_Format = 1D REAL
Dim_Size = 13107
X_Domain = Carbon
Dim_Title = Carbon13
Dim_Units = [ppm]
Dimensions = X
Spectrometer = JNM-ECZ400R/S1
Field_Strength = 9.389766[T] (400[MHz])
X_Acq_Duration = 0.13041664[s]
X_Domain = Carbon13
X_Freq = 100.52530333[MHz]
X_Offset = 100[ppm]
X_Points = 4096
X_Prescans = 0
X_Resolution = 7.6677332[Hz]
X_Sweep = 31.40703518[kHz]
X_Sweep_Clipped = 25.12562814[kHz]
Irr_Domain = Proton
Irr_Freq = 399.78219838[MHz]
Irr_Offset = 2.3655[ppm]
Blanking = 2[us]
Clipped = FALSE
Scans = 5000
Total_Scans = 5000
Relaxation_Delay = 4[s]
Recvr_Gain = 46
Tube_Diameter = 4[mm]
Mas_Frequency = 11.004[kHz]
Temp_Get = 20[dC]
Contact_Time = 2[ms]
X_Acq_Time = 0.13041664[s]
X_Dwell = 31.84[us]
X_Pulse = 0.1[us]
Irr_Amp_Cp = 70[%]
Irr_Amp_Dec = 100[%]
Irr_Amp_Prep = 100[%]
Irr_Atn = 3.9[dB]

b)

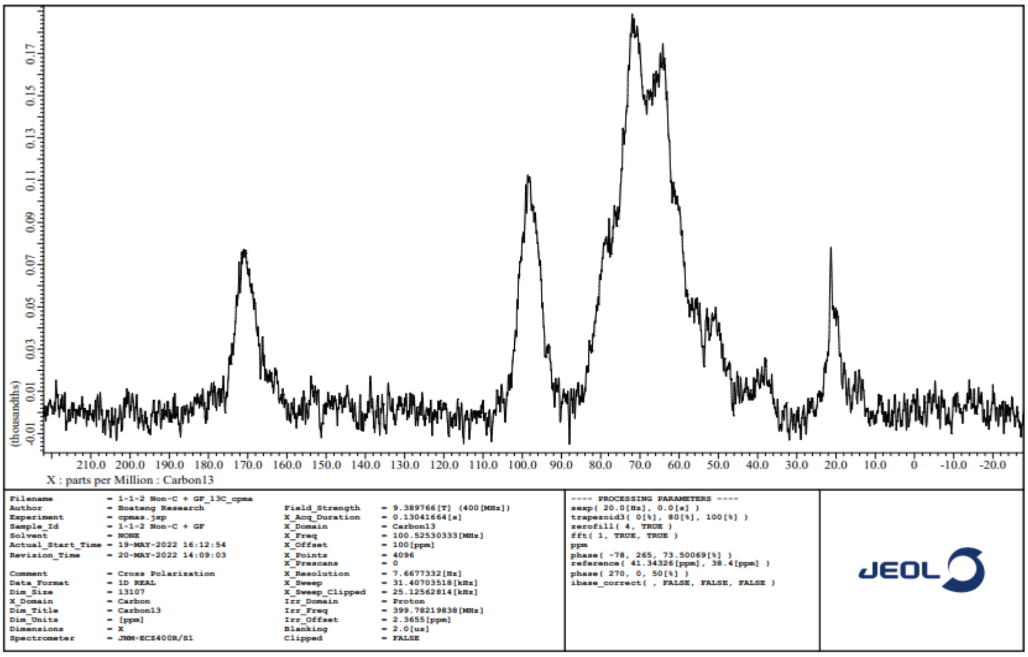

(thousandths)
X : parts per Million : Carbon13
Filename = 1-1-2 Non-C + GF_13C_cpma
Author = Boateng Research
Experiment = cpmas.jxp
Sample_Id = 1-1-2 Non-C + GF
Solvent = NONE
Actual_Start_Time = 19-MAY-2022 16:12:54
Revision_Time = 20-MAY-2022 14:09:03
Comment = Cross Polarization
Data_Format = 1D REAL
Dim_Size = 13107
X_Domain = Carbon
Dim_Title = Carbon13
Dim_Units = [ppm]
Dimensions = X
Spectrometer = JNM-ECZ400R/S1
Field_Strength = 9.389766[T] (400[MHz])
X_Acq_Duration = 0.13041664[s]
X_Domain = Carbon13
X_Freq = 100.52530333[MHz]
X_Offset = 100[ppm]
X_Points = 4096
X_Prescans = 0
X_Resolution = 7.6677332[Hz]
X_Sweep = 31.40703518[kHz]
X_Sweep_Clipped = 25.12562814[kHz]
Irr_Domain = Proton
Irr_Freq = 399.78219838[MHz]
Irr_Offset = 2.3655[ppm]
Blanking = 2.0[us]
Clipped = FALSE
---- PROCESSING PARAMETERS ----
sexp(20.0[Hz], 0.0[s])
trapezoid3(0[%], 80[%], 100[%])
zerofill(4, TRUE)
fft(1, TRUE, TRUE)
ppm
phase(-78, 265, 73.50069[%])
reference(41.34326[ppm], 38.4[ppm])
phase(270, 0, 50[%])
ibase_correct(, FALSE, FALSE, FALSE)

c)

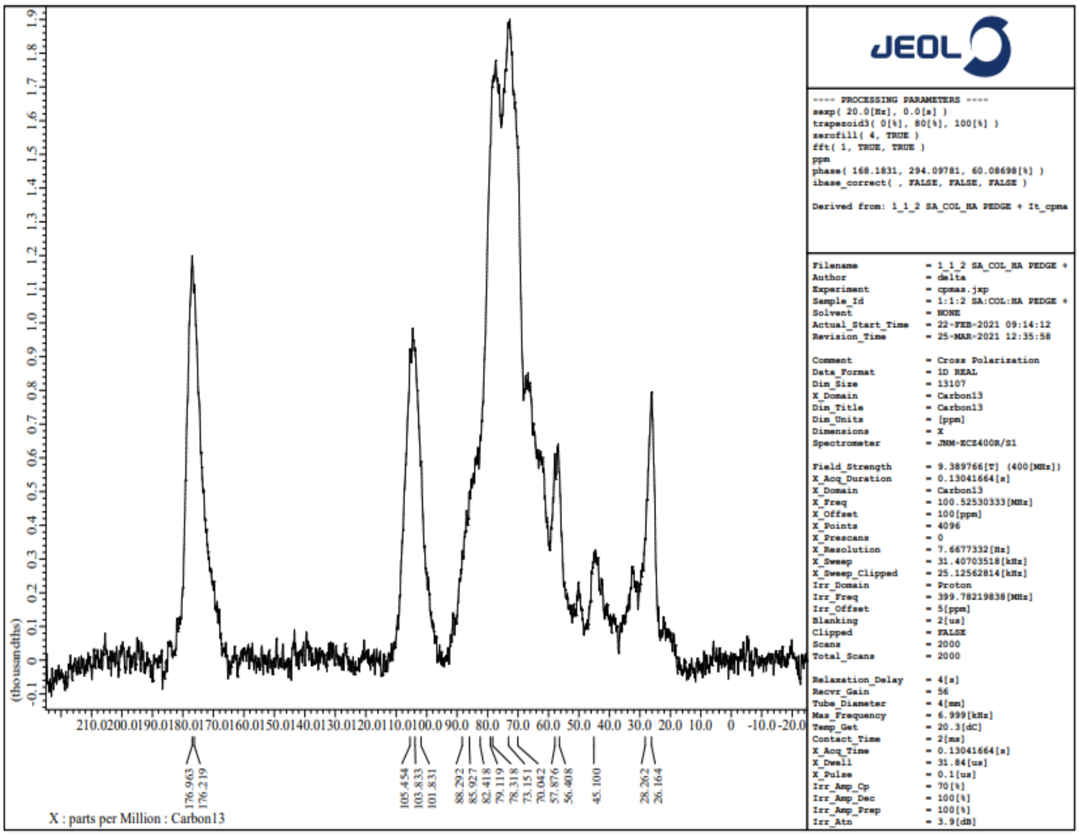

Figure S8 Representative NMR spectra for the (a) pure HA, (b) NC SA-G:FCOL:HA 1:1:2 b-FGF (c) IPC SA-G:FCOL:HA 1:1:2 b-FGF.

(a)

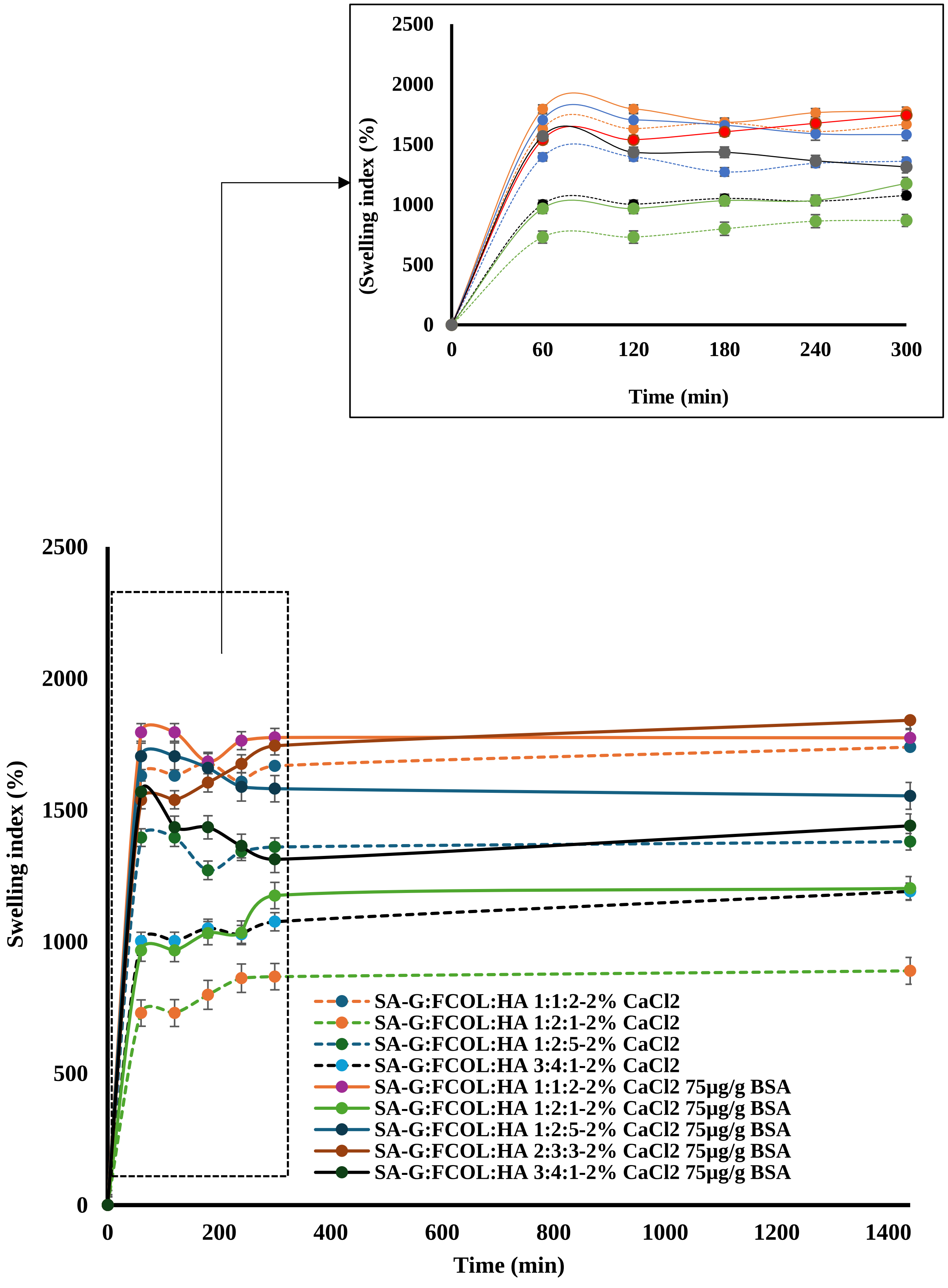
2500
2000
1500
1000
500
0
0
60
120
180
240
300
(Swelling index (%)
Time (min)
2500
2000
1500
1000
500
0
0
200
400
600
800
1000
1200
1400
Swelling index (%)
Time (min)
SA-G:FCOL:HA 1:1:2-2% CaCl2
SA-G:FCOL:HA 1:2:1-2% CaCl2
SA-G:FCOL:HA 1:2:5-2% CaCl2
SA-G:FCOL:HA 3:4:1-2% CaCl2
SA-G:FCOL:HA 1:1:2-2% CaCl2 75µg/g BSA
SA-G:FCOL:HA 1:2:1-2% CaCl2 75µg/g BSA
SA-G:FCOL:HA 1:2:5-2% CaCl2 75µg/g BSA
SA-G:FCOL:HA 2:3:3-2% CaCl2 75µg/g BSA
SA-G:FCOL:HA 3:4:1-2% CaCl2 75µg/g BSA

b)

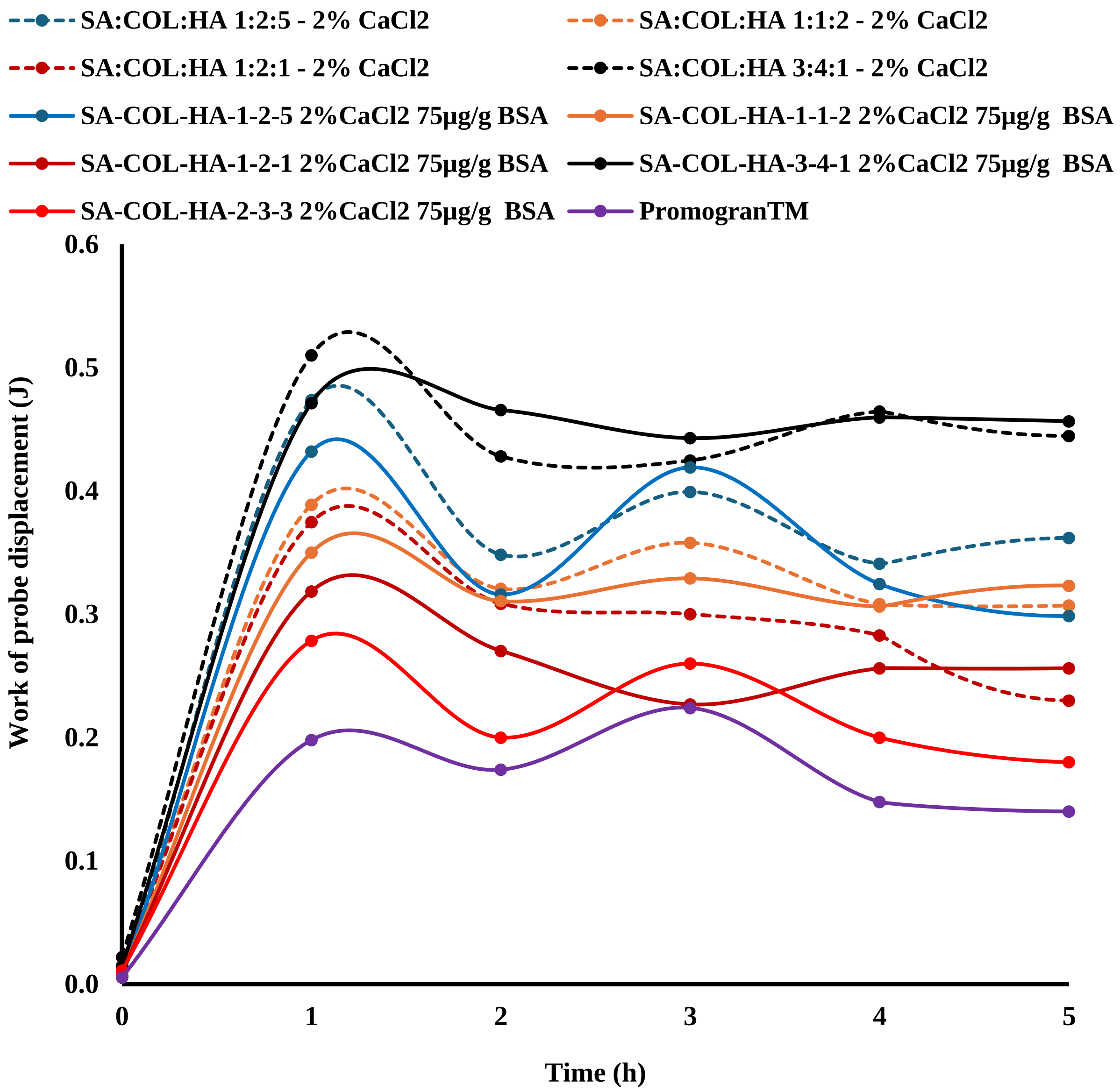

Figure S9i (a) the water uptake of CC scaffold dressings at different time points; (b) the total work of probe displacement at different time intervals for both blank and BSA loaded formulations

a)

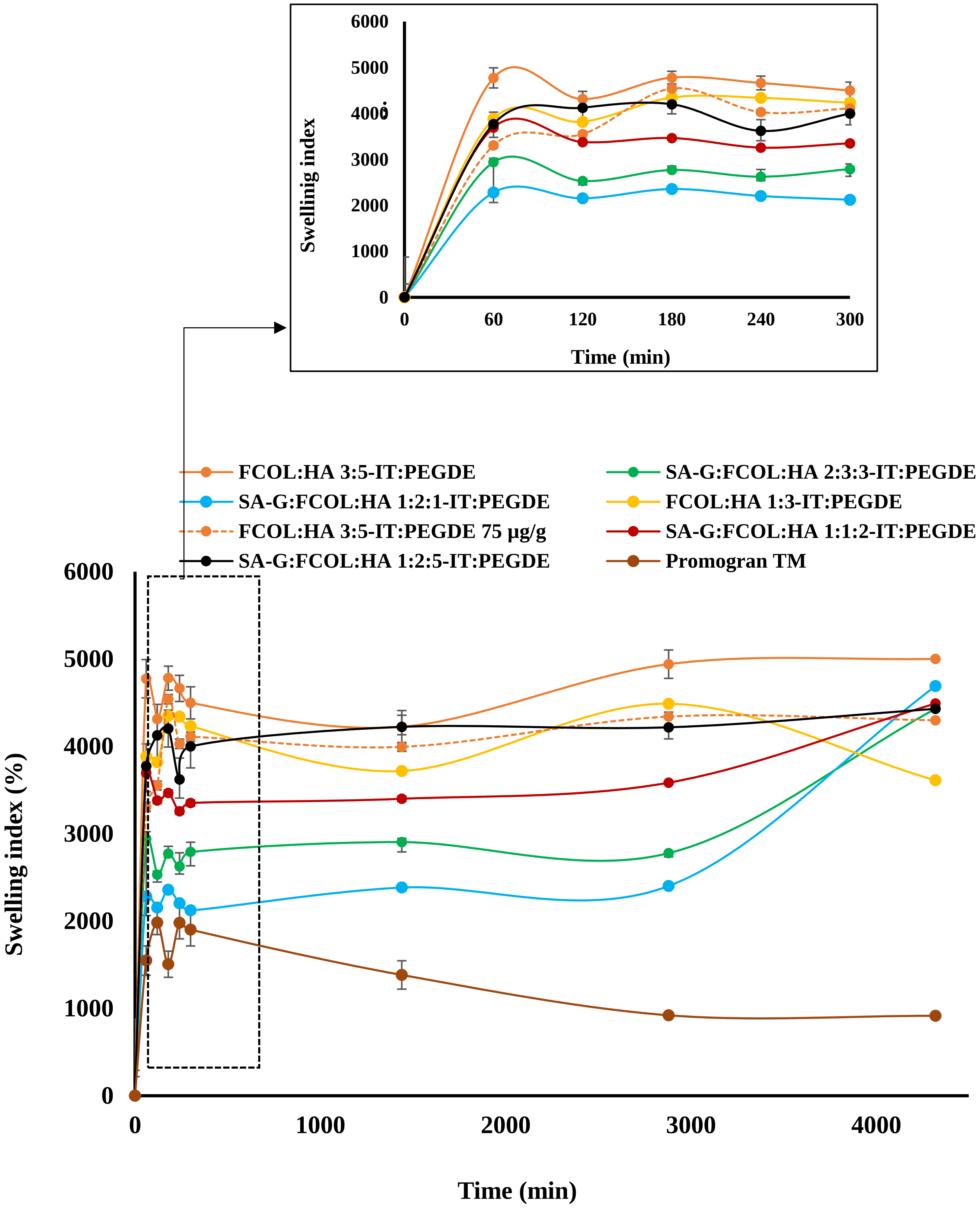

6000
5000
4000
3000
2000
1000
0
0
60
120
180
240
300
Swelling index
Time (min)
FCOL:HA 3:5-IT:PEGDE
SA-G:FCOL:HA 1:2:1-IT:PEGDE
FCOL:HA 3:5-IT:PEGDE 75 μg/g
SA-G:FCOL:HA 1:2:5-IT:PEGDE
SA-G:FCOL:HA 2:3:3-IT:PEGDE
FCOL:HA 1:3-IT:PEGDE
SA-G:FCOL:HA 1:1:2-IT:PEGDE
Promogran TM
6000
5000
4000
3000
2000
1000
0
0
1000
2000
3000
4000
Swelling index (%)
Time (min)

b)

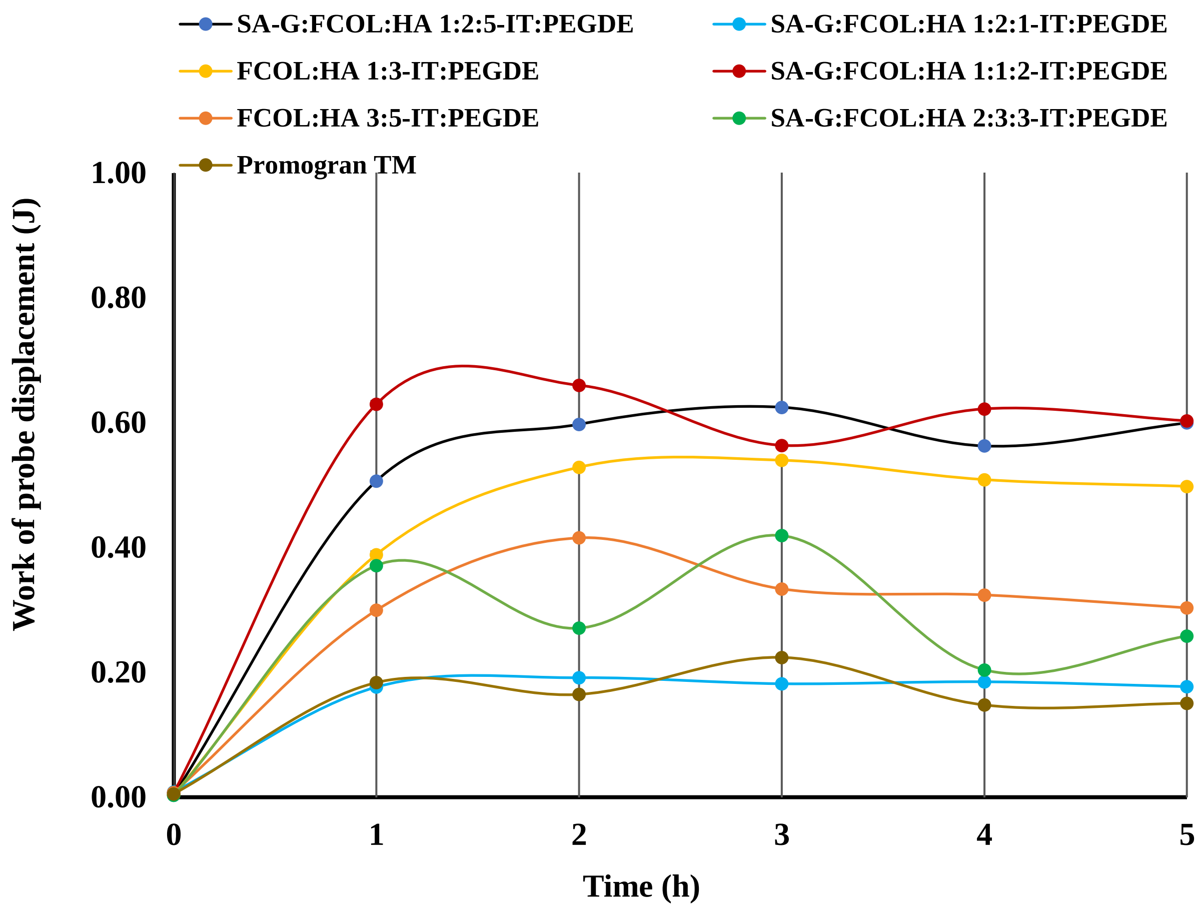

Figure S9ii (a) the water uptake of the IPC scaffolds at different time points; (b) the total work of probe displacement at different time intervals

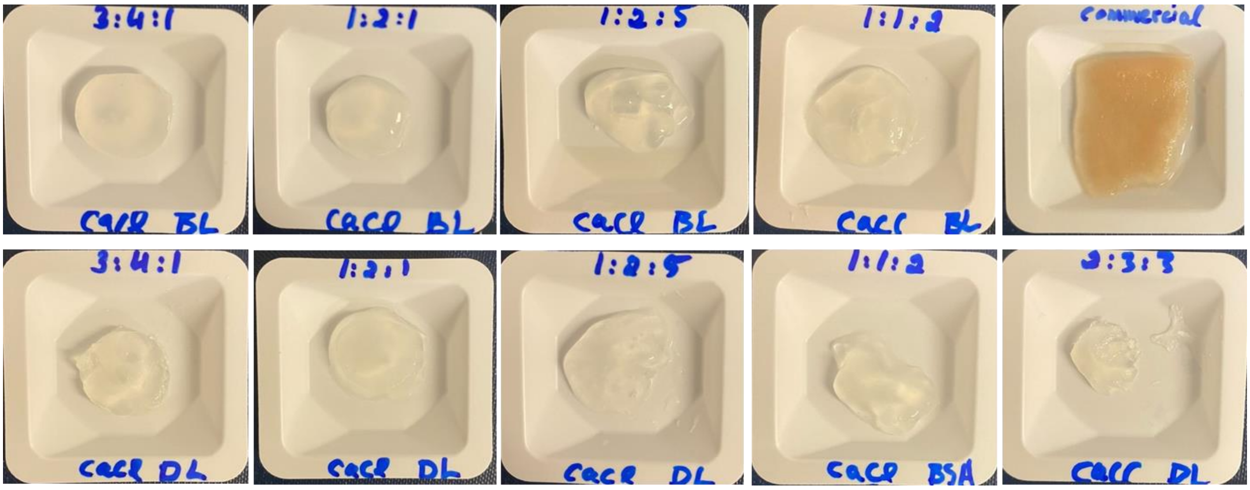

Figure S10a Morphological behavior of swelling and erosion of SA-G:COL:HA based scaffold dressings as blank (BL), drug loaded (DL) and commercial COL based wound dressing Promogran™ acquired after 72 h hydration in SWF pH 7.4

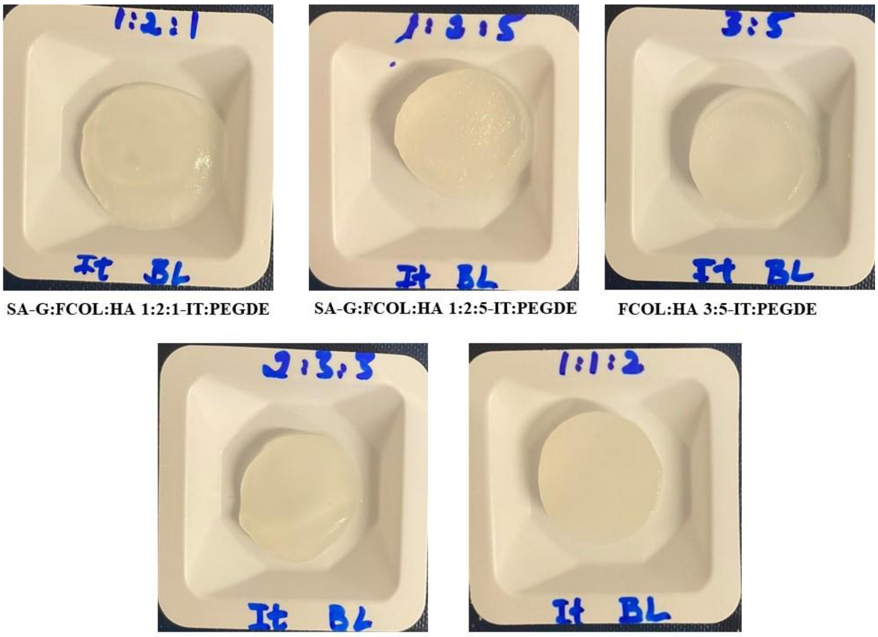

SA-G:FCOL:HA 1:2:1-IT:PEGDE SA-G:FCOL:HA 1:2:5-IT:PEGDE FCOL:HA 3:5-IT:PEGDE

SA-G:FCOL:HA 2:3:3-IT:PEGDE SA-G:FCOL:HA 1:1:2-IT:PEGDE

Figure S10b Morphological behaviour of swelling and erosion of SA-G:COL:HA based scaffolds as blank (BL), drug loaded (DL) acquired after 72 h hydration in SWF pH 7.

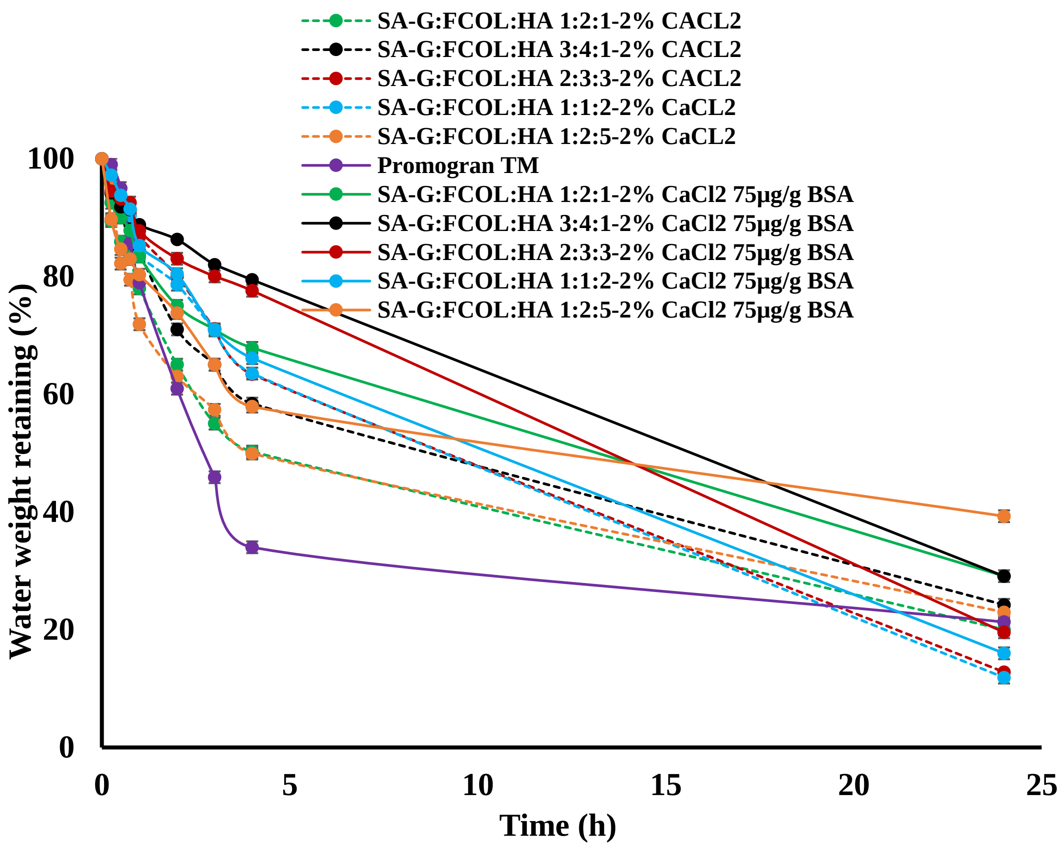

Figure 11a Evaporative water loss of both blank and BSA loaded CC scaffold dressings (*n* = 3) ± SD. The results did not show any significant difference between the blank and BSA loaded scaffold dressings

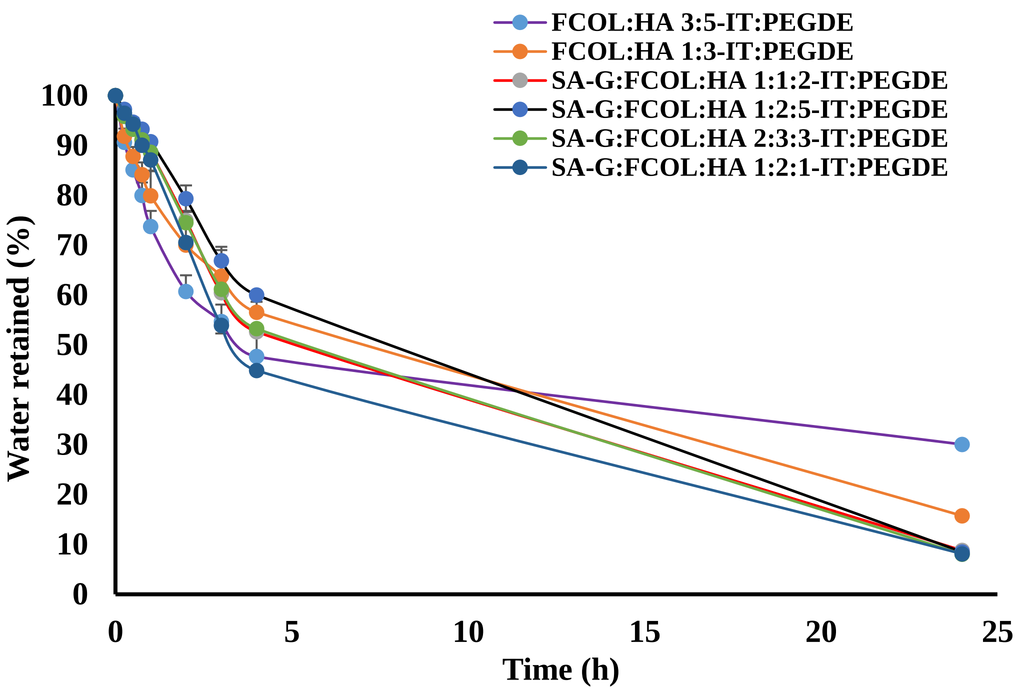

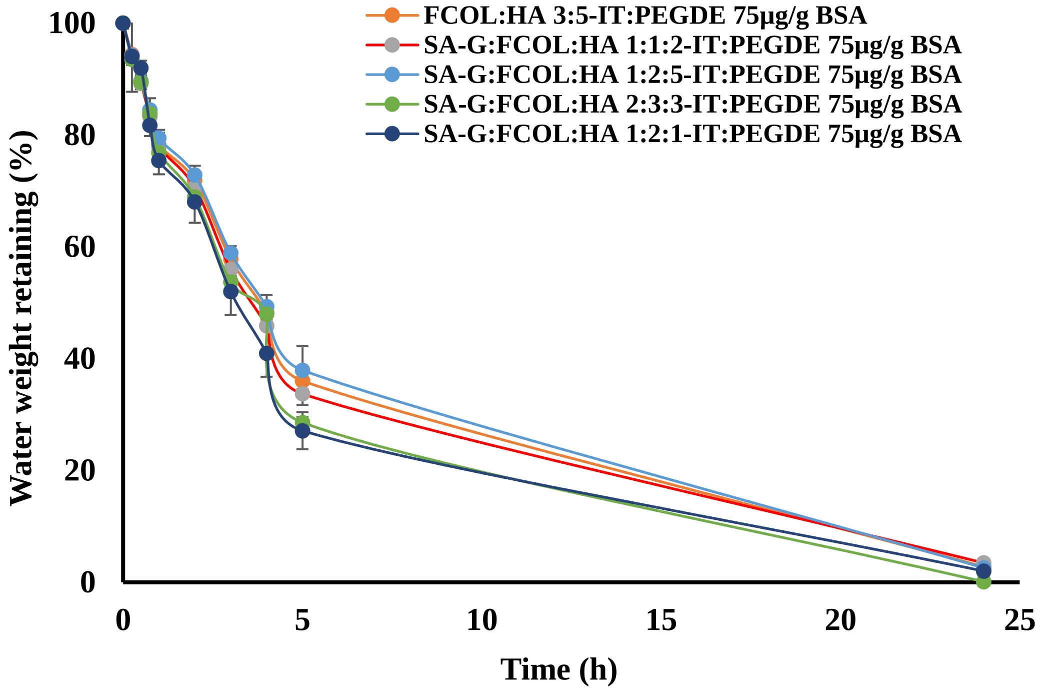

Figure S11b EWL profiles for blank and BSA loaded IPC scaffold dressings

(a)

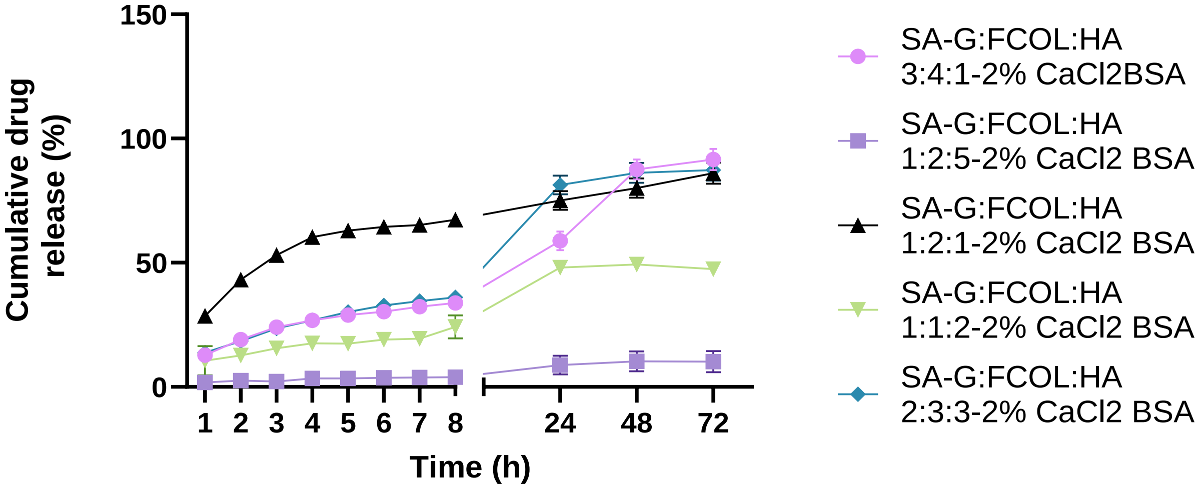

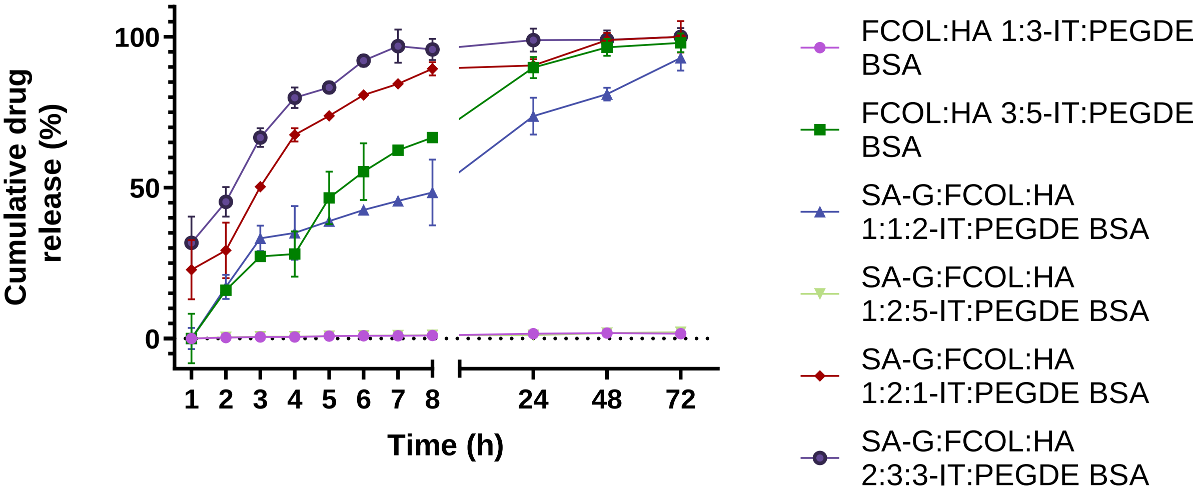

Figure S12 Shows percentage cumulative drug release profile of (a) 75µg/g BSA loaded SA-G:FCOL:HA CC scaffold dressings (*n* = 3 ± SD) and (b) *In vitro* drug dissolution profiles of BSA loaded IPC scaffold dressings. The inset shows the release profiles over the first 8 h, showing near linear release with time.

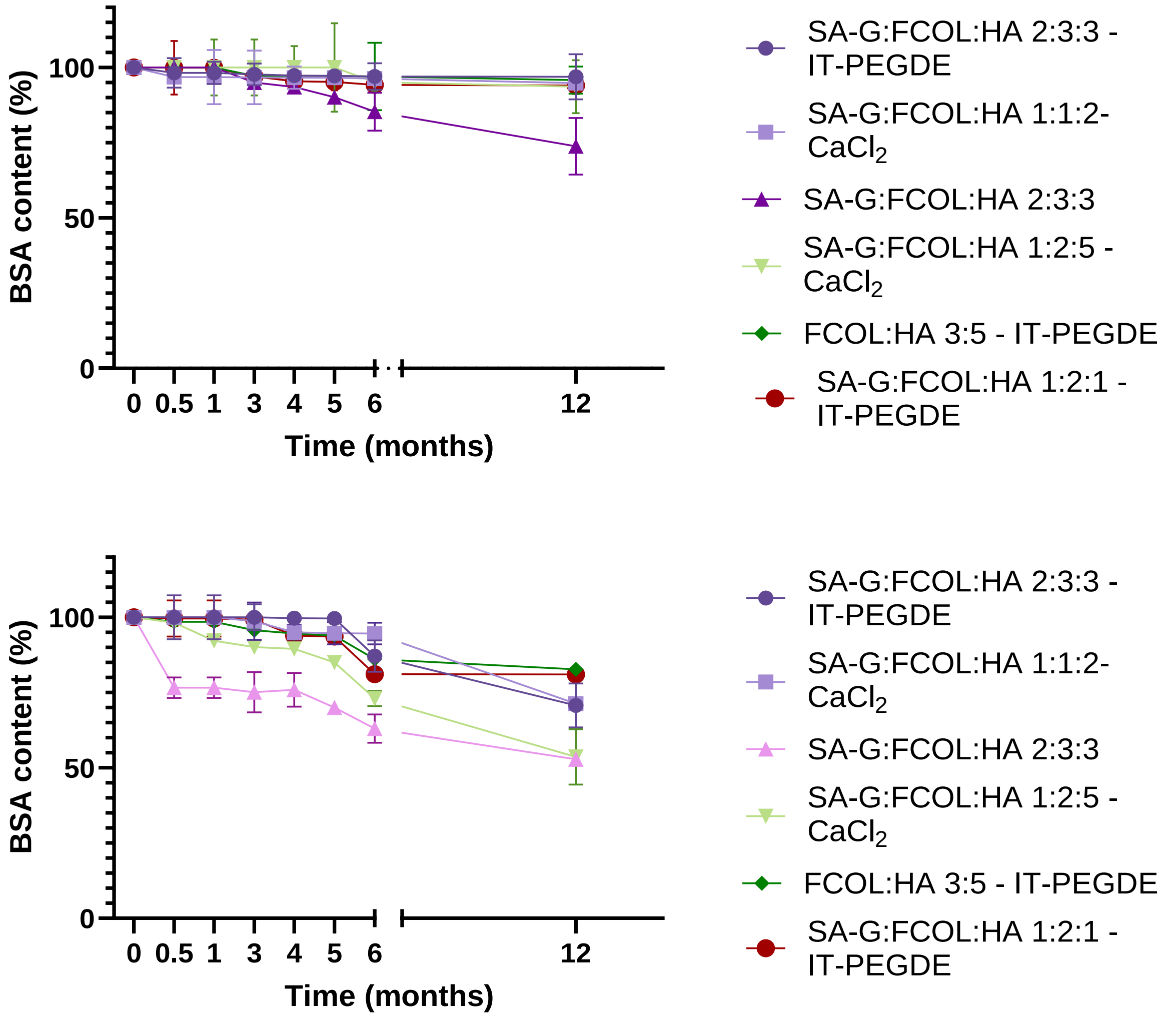

Figure S13 Stability curves obtained by SE -HPLC showing (a) BSA content after 12 months storage in the refrigerator (b) BSA content after 12 months storage under ICH conditions.

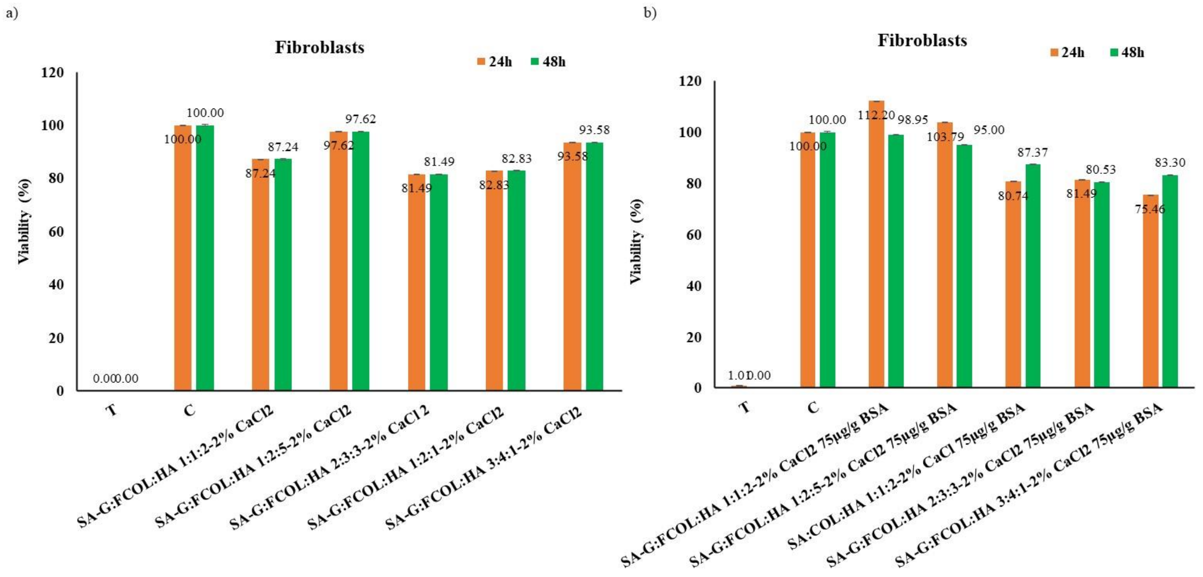

Figure S14a Cell viability of primary HDFs after exposure of a) blank SA-G:FCOL:HA-2% $CaCl_2$ b) BSA loaded SA-G:FCOL:HA-2% $CaCl_2$ for 24 and 48 h (mean ± SD n = 9)

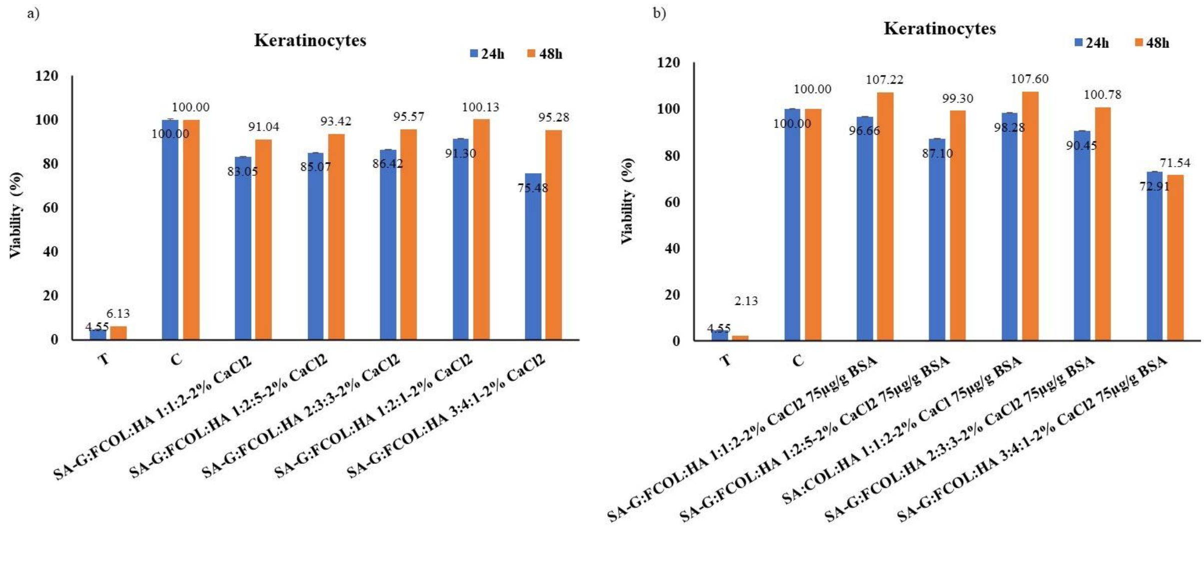

Figure S14b Cell viability of PEKs after exposure of a) blank SA-G:FCOL:HA-2% $CaCl_2$ b) BSA loaded SA-G:FCOL:HA-2% $CaCl_2$ for 24 and 48 h (mean ± SD n = 9).

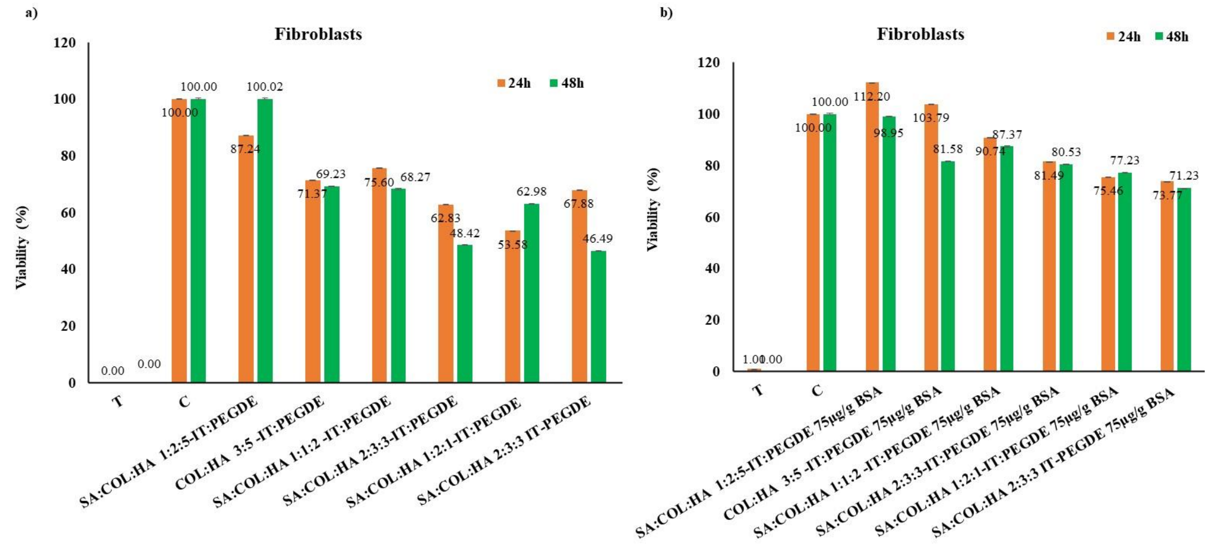

Figure S15a Cell viability of HDFs treated with the IPC scaffold dressings. The data are shown as the mean of the cell viability ± SD (n = 6). The statistical analysis shows a significant difference $p < 0.05$ between the NC and crosslinked formulations cells after 48 h

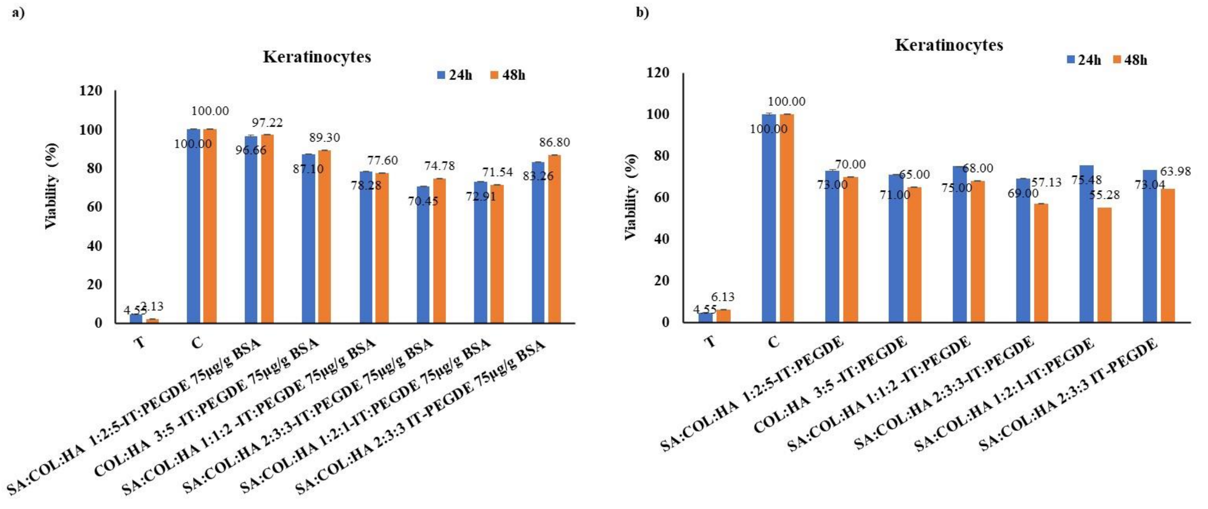

Figure S15b Cell viability of PEKs treated with the IPC scaffold dressings. The data are shown as the mean of the cell viability ± SD (n = 6). The statistical analysis shows a significant difference $p < 0.05$ between the NC and crosslinked formulations cells after 48 h

a)

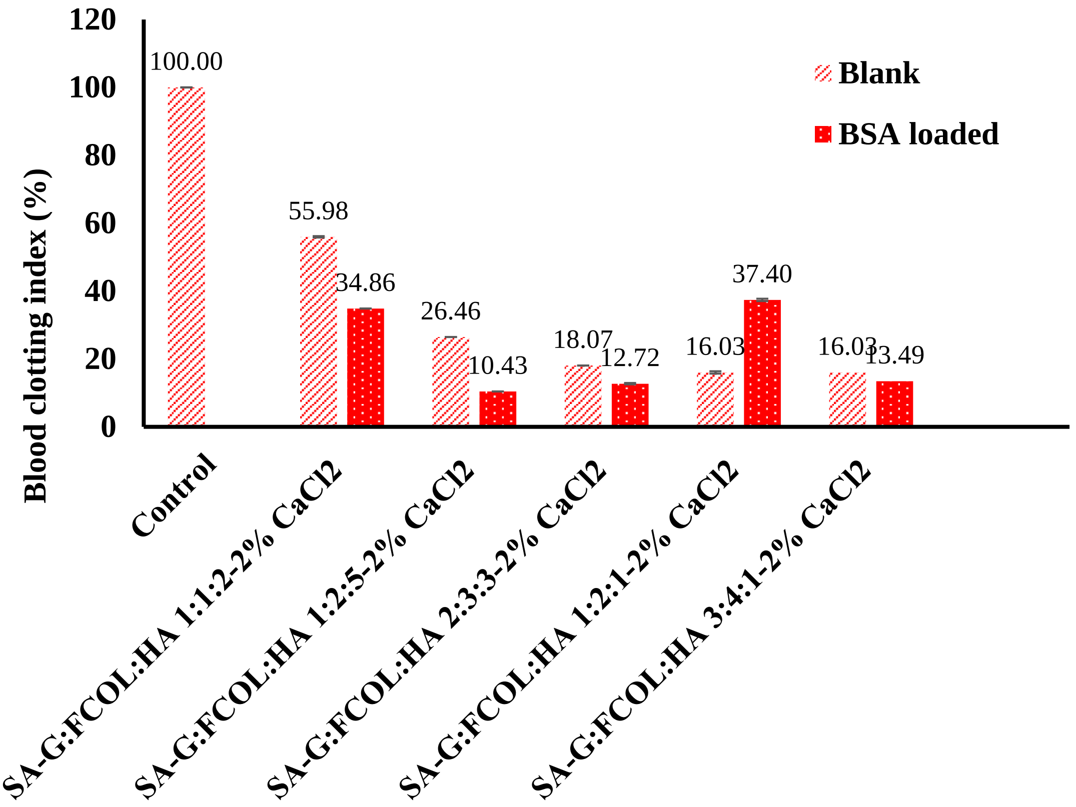

b)

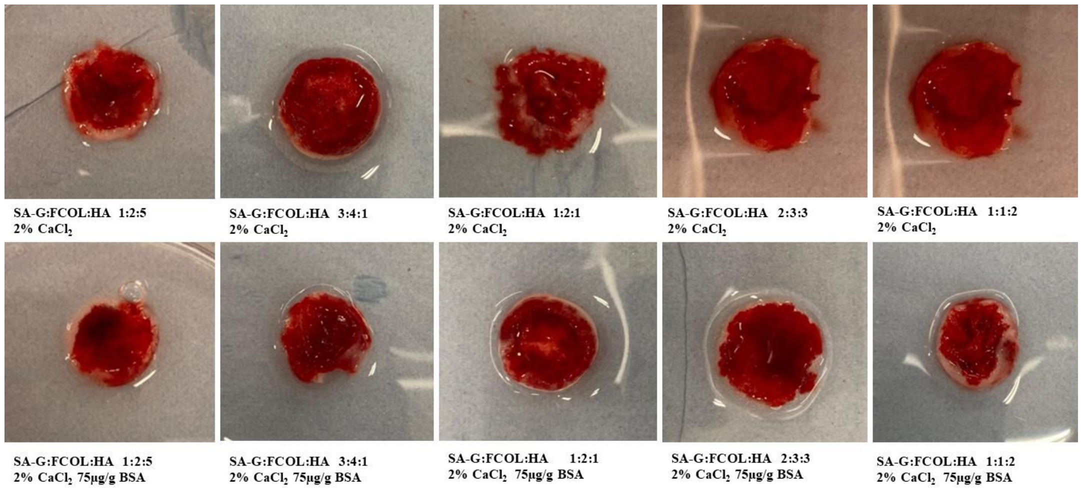

Figure S16 (a) the blood clotting index (BCI) of blank and BSA loaded scaffold dressings after 10 min contact with whole human blood. The difference in the BCI of control and the scaffold dressings was significant $p < 0.05$; (b) The digital images of $CaCl_2$ scaffold dressings after 10 min incubation. The experiment was performed as (n = 3). The visual observation showed denser clot formation. One way ANOVA statistical analysis showed a significant difference between the BCI of NC and the CC scaffold dressings $p < 0.05$

a)

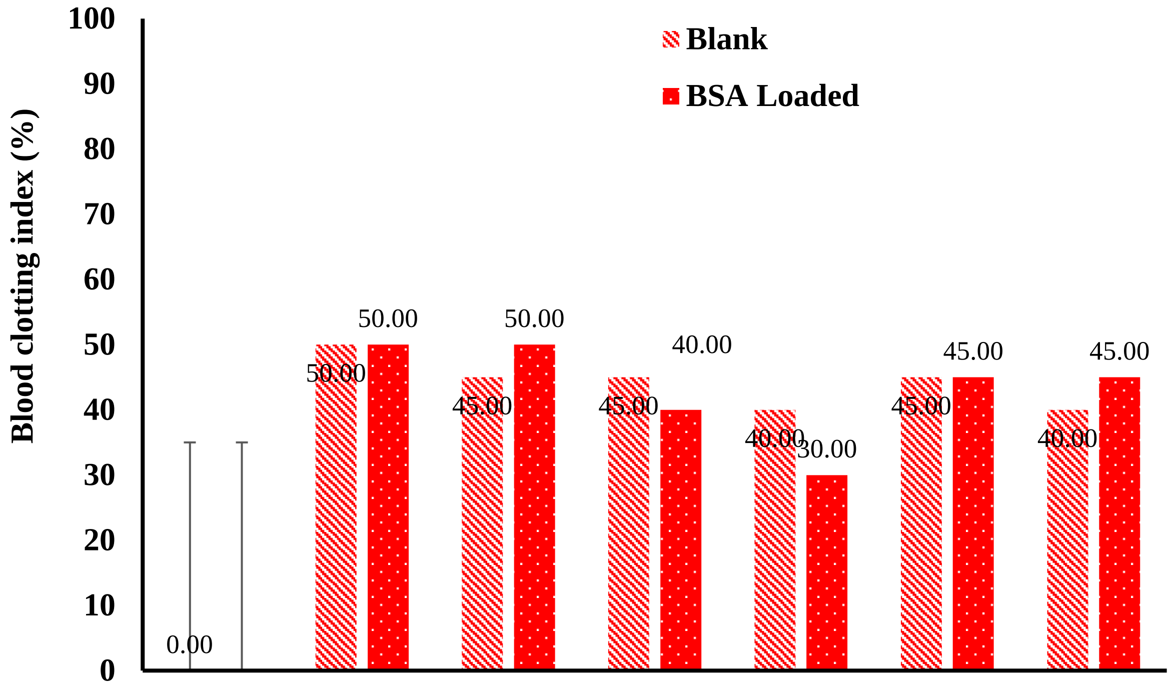

b)

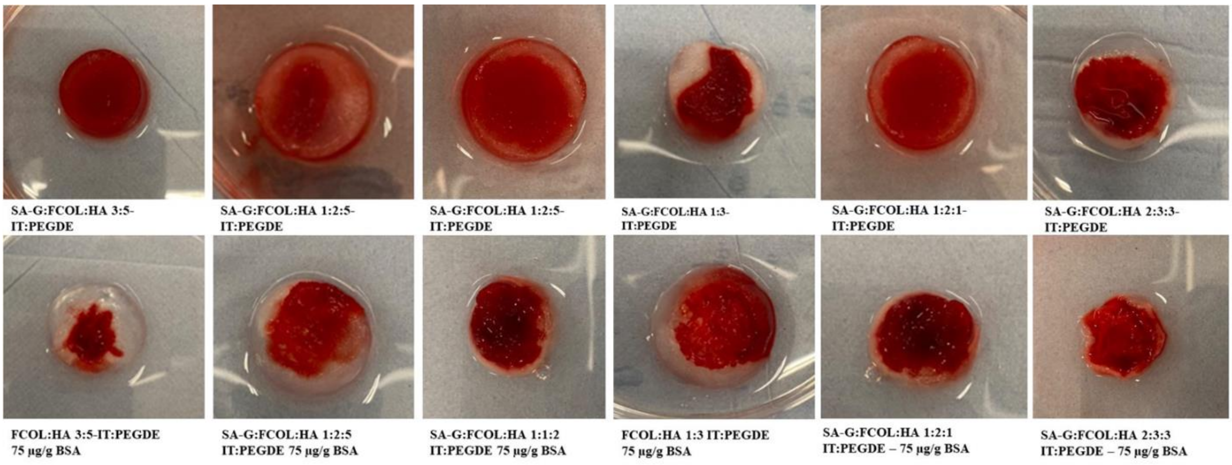

Figure S17 (a) The blood clotting index (BCI) for the blank and BSA loaded IPC scaffold dressings after being in contact with whole human blood, (b) the digital images of the scaffold dressings after 10 min incubation. The experiment was performed as ($n = 3$). One way ANOVA statistical analysis showed a significant difference between the control and the scaffold dressings $p < 0.05$

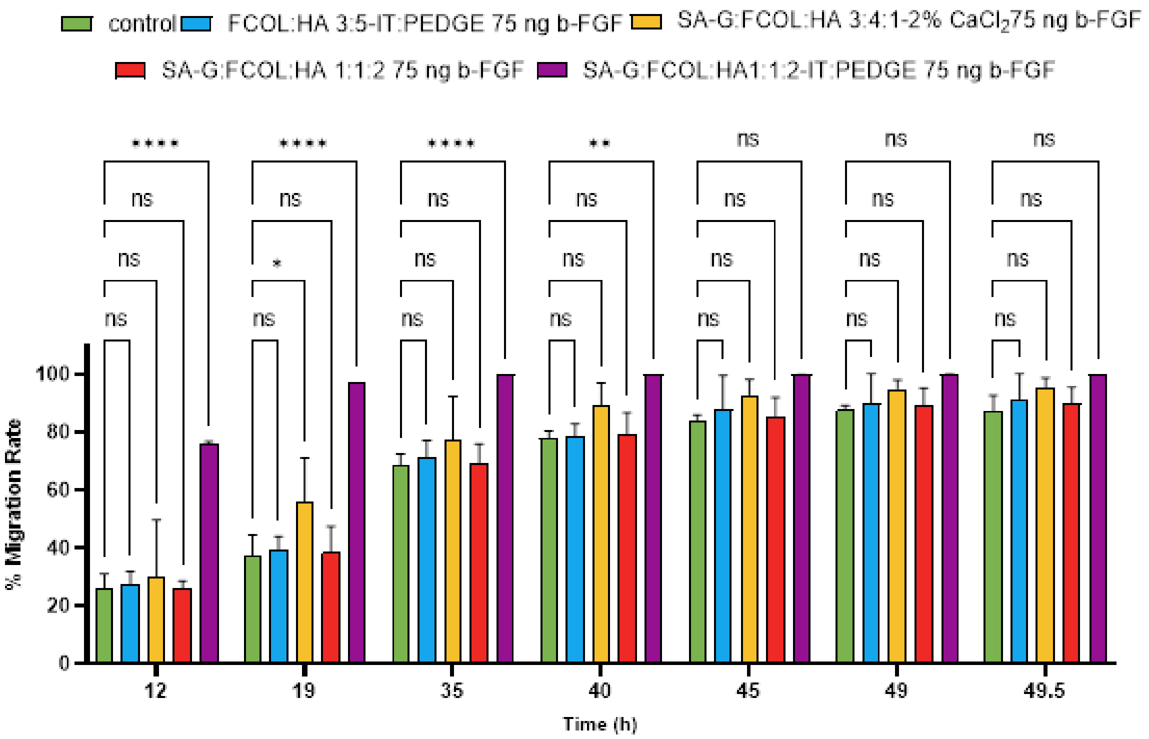

Figure S18a. The bar graph illustrates the comparison of migration rates across the different treatments at specified time points where the statistical significance is indicated by asterisks: **** p < 0.0001, ** p < 0.001, * p < 0.01, ns p > 0.05 (not significant)

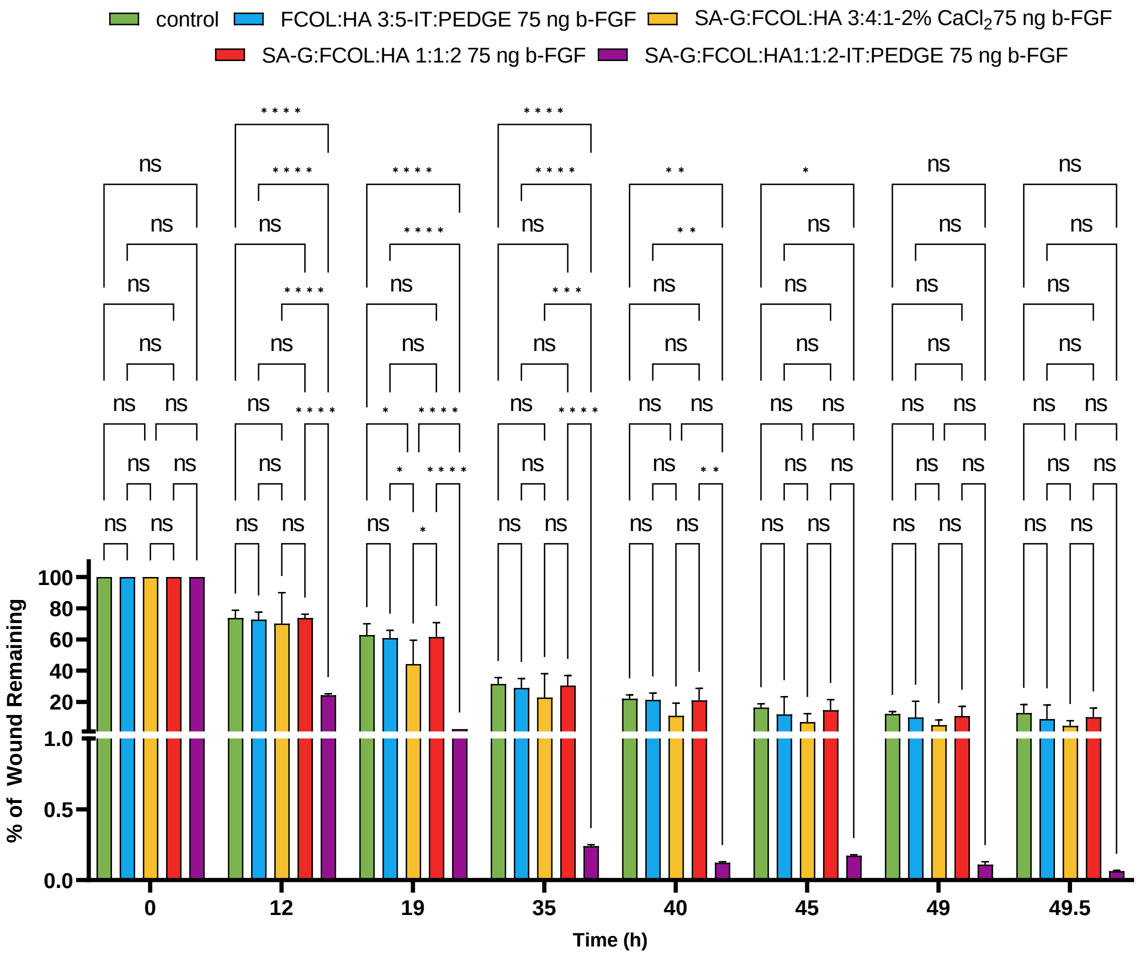

Figure S18b. The bar graph illustrates the comparison of wound closure across the different treatments at specified time points where the statistical significance is indicated by asterisks: **** $p < 0.0001$, ** $p < 0.001$, * $p < 0.01$, ns $p > 0.05$ (not significant)

Supplementary Tables

Table S1 Consistency and average work of cohesion of the (a) 2% CC and (b) IPC gels consisting of and different compositions of the polymers. All tests were conducted at room temperature and repeated 3 times (*n* = 3).

(a)

Sample	Firmness (N)	Consistency (N.Sec)	Cohesiveness (N)	Viscosity index (N.sec)
**SA-G:FCOL:HA 3:4:1-2% $CaCl_2$**	0.65 ± 0.01	1.74 ± 0.02	-0.66 ± 0.02	-0.45 ± 0.03
**SA-G:FCOL:HA 1:2:1-2% $CaCl_2$**	0.73 ± 0.02	2.00 ± 0.05	-0.08 ± 0.02	-0.50 ± 0.01
**SA-G:FCOL:HA 2:3:3-2% $CaCl_2$**	1.25 ± 0.03	3.38 ± 0.10	-1.36 ± 0.03	-0.98 ± 0.08
**SA-G:FCOL:HA 1:1:2-2% $CaCl_2$**	2.30 ± 0.01	5.42 ± 0.85	-1.49 ± 0.03	-1.85 ± 0.23
**SA-G:FCOL:HA 1:2:5-2% $CaCl_2$**	2.58 ± 0.03	6.58 ± 0.39	-2.39 ± 0.63	-1.97 ± 0.23
**SA-G:FCOL:HA 3:4:1-2% $CaCl_2$ 75µg/g BSA**	0.70 ± 0.03	1.94 ± 0.12	-0.78 ± 0.11	-0.61 ± 0.07
**SA-G:FCOL:HA 1:2:1-2% $CaCl_2$ 75µg/g BSA**	0.81 ± 0.02	2.20 ± 0.07	-1.00 ± 0.01	-0.55 ± 0.01
**SA-G:FCOL:HA 2:3:3-2% $CaCl_2$ 75µg/g BSA**	1.45 ± 0.00	3.50 ± 0.01	-1.35 ± 0.00	-1.50 ± 0.00
**SA-G:FCOL:HA 1:1:2-2% $CaCl_2$ 75µg/g BSA**	2.60 ± 0.02	5.63 ± 0.29	-1.70 ± 0.36	-1.96 ± 0.18
**SA-G:FCOL:HA 1:2:5-2% $CaCl_2$ 75µg/g BSA**	2.80 ± 0.01	6.72 ± 0.17	-2.50 ± 0.41	-2.05 ± 0.12

(b)

	**Firmness (N)**	**Consistency (N.sec)**	**Cohesiveness (N)**	**Viscosity index (N.sec)**
**FCOL:HA 1:3-IT:PEGDE**	1.99 ± 0.02	5.34 ± 0.17	-1.71 ± 0.12	-1.50 ± 0.18
**FCOL:HA 3:5-IT:PEGDE**	1.85 ± 0.03	4.87 ± 0.29	-1.95 ± 0.36	-1.36 ± 0.11
**SA-G:FCOL:HA 1:2:5-IT:PEGDE**	2.02 ± 0.01	5.34 ± 0.17	-1.83 ± 0.41	-1.71 ±0.01
**SA-G:FCOL:HA 1:1:2-IT:PEGDE**	1.31 ± 0.02	3.53 ± 0.12	-1.56 ± 0.11	-1.01 ± 0.07
**SA-G:FCOL:HA 2:3:3-IT:PEGDE**	0.74 ± 0.00	2.06 ± 0.07	-0.90 ± 0.01	-0.59 ± 0.01
**SA-G:FCOL:HA 1:2:1-IT:PEGDE**	0.38 ± 0.10	1.06 ± 0.01	-0.40 ± 0.01	-0.26 ± 0.02
**FCOL:HA 1:3-IT:PEGDE 75µg/g BSA**	1.85 ± 0.06	3.54 ± 0.17	-1.63 ± 0.02	-0.90 ± 0.23
**FCOL:HA 3:5-IT:PEGDE 75µg/g BSA**	1.64 ± 0.02	3.74 ± 0.50	-1.62 ± 0.37	-0.81 ± 0.23
**SA-G:FCOL:HA 1:2:5-IT:PEGDE 75µg/g BSA**	1.95 ± 0.01	3.64 ± 0.30	-1.79 ± 0.62	-0.96 ± 0.57
**SA-G:FCOL:HA 1:1:2-IT:PEGDE 75µg/g BSA**	1.26 ± 0.02	3.41 ± 0.10	-1.55 ± 0.02	-0.85 ± 0.11
**SA-G:FCOL:HA 2:3:3-IT:PEGDE 75µg/g BSA**	0.67 ± 0.02	1.86 ± 0.10	-0.78 ± 0.02	-0.49 ± 0.03
**SA-G:FCOL:HA 1:2:1-IT:PEGDE 75µg/g BSA**	0.37 ± 0.12	1.03 ± 0.07	-0.38 ± 0.03	-0.27 ± 0.02

Table S2a Exudate handling properties of blank and BSA loaded CC scaffold dressings ($n = 3 \pm SD$)

**Sample name**	**Porosity (%) ± SD**	**WVTR (g/m^2 day^{-1}) ± SD**	**AW (%) ± SD**	**EWC (%) ± SD**
**SA-G:FCOL:HA 3:4:1-2% $CaCl_2$**	75 ± 2	3684 ± 10	503 ± 76	83 ± 2
**SA-G:FCOL:HA 2:3:3-2% $CaCl_2$**	72 ± 9	3337 ± 2588	948 ± 63	90 ± 1
**SA-G:FCOL:HA 1:1:2 - 2% $CaCl_2$**	74 ± 7	4037 ± 1243	1046 ± 251	90 ± 2
**SA-G:FCOL:HA 1:2:5-2% $CaCl_2$**	75 ± 2	2933± 343	801 ± 124	89 ± 2
**SA-G:FCOL:HA 1:2:1-2% $CaCl_2$ 75 µg/g BSA**	73 ± 4	2797 ± 236	675 ± 219	86 ± 4
**SA-G:FCOL:HA 3:4:1-2% $CaCl_2$ 75 µg/g BSA**	74 ± 0	2590 ± 131	1129 ± 22	90 ± 0
**SA-G:FCOL:HA 2:3:3-2% $CaCl_2$ 75 µg/g BSA**	81 ± 7	4010 ± 2708	975 ± 167	91 ± 2
**SA-G:FCOL:HA 1:1:2-2% $CaCl_2$ 75 µg/g BSA**	76 ± 3	2552 ± 47	633 ± 94	86 ± 2
**SA-G:FCOL:HA 1:2:5-2% $CaCl_2$ 75 µg/g BSA**	79 ± 3	2478 ± 58	809 ± 171	89 ± 2
**SA-G:FCOL:HA 3:4:1-2% $CaCl_2$ 75 ng b-FGF**	90 ± 1	2898 ± 698	416 ± 57	95 ± 1
**SA-G:FCOL:HA 1:1:2 75ng b-FGF**	70 ± 10	4254 ± 50	921 ± 44	92 ± 2
**SA-G:FCOL:HA 1:1:2-IT:PEDGE 75 ng b-FGF**	82 ± 3	4181 ± 206	1190 ± 16	95 ± 1
**FCOL:HA 3:5-IT:PEDGE 75 ng b-FGF**	87 ± 1	1685 ± 26	1378 ± 99	93 ± 0
**Promogran™**	86 ± 4	49460 ± 208	132 ± 117	63 ± 19

Table S2b Exudate handling properties of IPC blank and BSA loaded scaffolds ($n = 3 \pm SD$)

**Sample name**	**Porosity (%) ± SD**	**WVTR (g/m^2day^{-1}) ± SD**	**AW (%) ± SD**	**EWC (%) ± SD**
**FCOL:HA 3:5-IT:PEGDE**	86 ± 20	2368 ± 59	416 ± 57	80 ± 2
**FCOL:HA 1:3-IT:PEGDE**	85 ± 40	2195 ± 28	921 ± 44	90 ± 1
**SA-G:FCOL:HA 1:2:1-IT:PEGDE**	80 ± 20	2340 ± 851	1190 ± 16	93 ± 1
**SA-G:FCOL:HA 2:3:3-IT:PEGDE**	81 ± 10	2354 ± 23	1378 ± 99	93 ± 0
**SA-G:FCOL:HA 1:1:2-IT:PEGDE**	77 ± 11	2335± 680	1372 ±1	93 ± 0
**SA-G:FCOL:HA 1:2:5-IT:PEGDE**	82 ± 10	2328 ± 10	1312 ± 80	93 ± 0
**FCOL:HA 3:5-IT:PEGDE 75µg/g BSA**	76 ± 9	2209 ± 59	1612 ± 57	80 ± 6
**FCOL:HA 1:3-IT:PEGDE 75µg/g BSA**	68 ± 9	2200 ± 28	1798 ± 27	95 ± 0
**SA-G:FCOL:HA 1:2:1-IT:PEGDE 75µg/g BSA**	75 ± 2	1755 ± 851	1301 ± 58	95 ± 1
**SA-G:FCOL:HA 2:3:3-IT:PEGDE 75µg/g BSA**	75 ± 7	1686 ± 25	1619 ± 22	95 ± 2
**SA-G:FCOL:HA 1:1:2-IT:PEGDE 75µg/g BSA**	80 ± 4	2172 ± 560	1576 ± 65	94 ± 1
**SA-G:FCOL:HA 1:2:5-IT:PEGDE 75µg/g BSA**	75 ± 2	1632 ± 14	1504 ±87	94 ± 2

Table S3 Shows the BSA release data fitted into different kinetic models.

Scaffold dressings formulations	Higuchi		Korsmeyer-Peppas			Zero order		First order		Hixson-Crowell	
	K_H	R^2	K_{K-P}	n	R^2	K_0	R^2	K_1	R^2	K_{H-C}	R^2
SA-G:FCOL:HA 2:3:3-2% $CaCl_2$ BSA	1.76	0.99	0.66	0.10	0.93	0.07	0.57	0.09	0.92	5.37	0.21
SA-G:FCOL:HA 1:1:2-2% $CaCl_2$ BSA	1.07	0.98	0.60	0.18	0.59	0.04	0.51	0.20	0.05	6.90	0.35
SA-G:FCOL:HA 1:2:5-2% $CaCl_2$ BSA	0.19	0.96	0.23	0.19	0.58	0.03	0.60	0.01	0.86	8.99	0.47
SA-G:FCOL:HA 1:2:1-2% $CaCl_2$ BSA	3.76	0.99	0.87	0.69	0.59	0.06	0.04	0.05	0.92	6.90	0.35
SA-G:FCOL:HA 3:4:1-2% $CaCl_2$ BSA	1.68	0.99	0.74	0.49	0.60	0.03	0.01	0.09	0.93	5.59	0.23
FCOL:HA 1:3- IT:PEGDE BSA	1.56	0.89	0.12	0.60	0.97	0.02	0.90	0.01	0.03	0.01	0.01
FCOL:HA 3:5- IT:PEGDE BSA	2.61	0.94	0.45	0.48	0.90	0.09	0.80	0.01	0.17	0.01	0.51
SA-G:FCOL:HA 1:1:2-IT:PEGDE BSA	1.32	0.80	0.37	0.48	0.91	0.02	0.60	0.01	0.01	0.01	0.51
SA-G:FCOL:HA 1:2:5-IT:PEGDE BSA	1.16	0.77	0.36	0.50	0.90	0.02	0.57	0.01	0.03	0.01	0.27
SA-G:FCOL:HA 1:2:1-IT:PEGDE BSA	1.37	0.73	0.08	0.64	0.83	0.02	0.33	0.00	0.13	4.88	0.20
SA-G:FCOL:HA 2:3:3-IT:PEGDE BSA	1.45	0.68	0.08	0.86	0.90	0.02	0.34	0.00	0.32	5.95	0.28

Supplementary References

M. Dovedytis, Z.J. Liu, and Bartlett, S. “Hyaluronic acid and its biomedical applications: A review. Engineered Regeneration” vol. 1, pp. 102–113. 2020, doi: 10.1016/j.engreg.2020.10.001.

H. Lenormand, and J.-C. Vincent, “pH effects on the hyaluronan hydrolysis catalyzed by hyaluronidase in the presence of proteins: Part II. The electrostatic hyaluronan – Protein complexes”, *Carbohydrate Polymers*, vol. 85, no 2, pp. 303–311. 2011, doi: 10.1016/j.carbpol.2011.02.007.

R. Li, Z. Wu, Y. Wangb, L. Ding, and Wang, Y. “Role of pH-induced structural change in protein aggregation in foam fractionation of bovine serum albumin”. *Biotechnology Reports* vol. 9, pp. 46–52. 2016, doi: 10.1016/j.btre.2016.01.002.

F. Tuğcu-Demiröz, “Vaginal delivery of benzydamine hydrochloride through liposomes dispersed in mucoadhesive gels”. Chemical and Pharmaceutical Bulletin, 2017.

K. Valachová and L. Šoltés, “Hyaluronan as a Prominent Biomolecule with Numerous Applications in Medicine.” *International Journal of Molecular Sciences*, vol. 22, no. 13, p. 7077, 2021, doi: 10.3390/ijms22137077.

S. Shanmuga Doss, N. P. Bhatt, and G. Jayaraman, “Improving the accuracy of hyaluronic acid molecular weight estimation by conventional size exclusion chromatography.” *Journal of Chromatography B*, vol. 1060, pp. 255-261, 2017, doi: 10.1016/j.jchromb.2017.06.006.
